# Proteogenomic insights into the biology and treatment of pan-melanoma

**DOI:** 10.1038/s41421-024-00688-7

**Published:** 2024-07-23

**Authors:** Hang Xiang, Rongkui Luo, Yunzhi Wang, Bing Yang, Sha Xu, Wen Huang, Shaoshuai Tang, Rundong Fang, Lingli Chen, Na Zhu, Zixiang Yu, Sujie Akesu, Chuanyuan Wei, Chen Xu, Yuhong Zhou, Jianying Gu, Jianyuan Zhao, Yingyong Hou, Chen Ding

**Affiliations:** 1 State Key Laboratory of Genetic Engineering, School of Life Sciences, Human Phenome Institute, Department of Plastic and Reconstructive Surgery, Zhongshan Hospital, Fudan University, Shanghai, China; 2 Department of Pathology, Zhongshan Hospital, Fudan University, Shanghai, China; 3 State Key Laboratory of Oncology in South China, Guangdong Provincial Clinical Research Center for Cancer, Sun Yat-sen University Cancer Center, Guangzhou, Guangdong China; 4 Department of Plastic and Reconstructive Surgery, Zhongshan Hospital, Fudan University, Shanghai, China; 5 Department of Medical Oncology, Zhongshan Hospital, Fudan University, Shanghai, China; 6 Department of Plastic and Reconstructive Surgery, Zhongshan Hospital (Xiamen), Fudan University, Shanghai, China; 7 Institute for Developmental and Regenerative Cardiovascular Medicine, MOE-Shanghai Key Laboratory of Children’s Environmental Health, Xinhua Hospital, Shanghai Jiao Tong University School of Medicine, Shanghai, China

**Keywords:** Melanoma, Proteomics

## Abstract

Melanoma is one of the most prevalent skin cancers, with high metastatic rates and poor prognosis. Understanding its molecular pathogenesis is crucial for improving its diagnosis and treatment. Integrated analysis of multi-omics data from 207 treatment-naïve melanomas (primary-cutaneous-melanomas (CM, *n* = 28), primary-acral-melanomas (AM, *n* = 81), primary-mucosal-melanomas (MM, *n* = 28), metastatic-melanomas (*n* = 27), and nevi (*n* = 43)) provides insights into melanoma biology. Multivariate analysis reveals that *PRKDC* amplification is a prognostic molecule for melanomas. Further proteogenomic analysis combined with functional experiments reveals that the *cis*-effect of *PRKDC* amplification may lead to tumor proliferation through the activation of DNA repair and folate metabolism pathways. Proteome-based stratification of primary melanomas defines three prognosis-related subtypes, namely, the ECM subtype, angiogenesis subtype (with a high metastasis rate), and cell proliferation subtype, which provides an essential framework for the utilization of specific targeted therapies for particular melanoma subtypes. The immune classification identifies three immune subtypes. Further analysis combined with an independent anti-PD-1 treatment cohort reveals that upregulation of the MAPK7-NFKB signaling pathway may facilitate T-cell recruitment and increase the sensitivity of patients to immunotherapy. In contrast, PRKDC may reduce the sensitivity of melanoma patients to immunotherapy by promoting DNA repair in melanoma cells. These results emphasize the clinical value of multi-omics data and have the potential to improve the understanding of melanoma treatment.

## Introduction

Melanoma is the most aggressive type of cancer and exhibits robust treatment resistance. According to global cancer statistics, the incidence of melanoma is 3/100,000. More than 90 thousand new cases in the USA and more than 30 thousand new cases in China are estimated each year^1–3^. The incidence of melanoma has risen rapidly over the past few decades. Although most melanoma patients are cured surgically (> 90%) if diagnosed early, ~15% of melanomas further metastasize, with a poor survival rate. Therapeutic advances, including the use of mitogen-activated protein kinase (MAPK) inhibitors and immunotherapy, are promising for improving the survival rate^4,5^.

Melanomas arise from pigment cells, namely, melanocytes, which are located in the basal layer of the epidermis. Melanoma is commonly observed on the skin and can be classified based on tumor location, nonhair-bearing skin (palms of the hand, soles of the feet) (acral melanomas; AMs) or nonextremities (cutaneous melanomas; CMs)^6^. Epidemiological studies have shown that CMs mainly occur in white populations with fair skin, whereas pigmented populations from Asia mainly develop AMs. Moreover, a significant proportion of AMs lack mutations in *BRAF*, *NRAS*, or *NF1*^7^; thus, AM patients could hardly benefit from BRAF and MEK inhibitors. Therefore, there is an urgent need to identify novel driving genomic variants in AMs.

Mucosal melanoma (MM) accounts for 0.8%–3.7% of melanomas in the Western population^8^ but accounts for 20%–30% of melanomas in the Chinese population. MM is typically detected at a more advanced stage, which poses treatment challenges compared to CMs^9^. Genomic studies have indicated that MMs exhibit a markedly different genomic landscape than CMs^10,11^. Despite the progress, this knowledge has not yet been translated into efficacious systemic therapies. The drugs that have been approved to treat advanced CMs work less well for most patients with MMs. Novel targets and treatment strategies for MM patients are clearly needed.

Previous genomic and transcriptomic studies have elucidated the molecular landscape of melanomas^7,12^. For instance, The Cancer Genome Atlas (TCGA) published a melanoma study involving 331 melanoma patients, describing the landscape of somatic alterations in CMs and identifying multiple significant driver genes, including *BRAF*, *NRAS*, *TP53*, *NF1*, and *CDKN2A*^12^. Subsequently, Hayward et al. conducted a whole-genome analysis on 183 melanoma samples and revealed diverse mutational features across melanoma subtypes^7^. Moreover, pathways such as the DNA damage response and cell proliferation pathways have been reported to be associated with the genome instability of melanoma, and mutations, including *ATM* and *ATR*, have been reported in some melanomas^13,14^. Despite progress, studies on such aspects have focused on a single data platform, and the mechanism underlying gene alterations that drive cancer phenotypes in patients with melanomas remains unknown.

Melanomas are characterized by high immunogenicity, and immune checkpoint blockade (ICB) has become the first-line treatment for melanoma. However, owing to the high genetic heterogeneity, the clinical efficacy of ICBs differs among patients. To develop more effective therapies, combinational strategies are being explored. For instance, Sullivan et al. reported that combination clinical trials of BRAF, MEK, and PD-1/PD-L1 antagonists suggested an overlapping benefit between BRAF-targeted approaches and immunotherapy^15^. However, a considerable number of patients are resistant to current combination treatments. Given the clinical momentum in combining targeted therapy and immunotherapy, it is important to identify novel druggable genomic alterations and determine their impact on the tumor immune microenvironment.

In this study, we conducted extensive genomic, transcriptomic, proteomic, and phosphoproteomic characterization of melanoma samples obtained from a large Chinese cohort of 207 cases, including 137 primary-melanoma cases (28 CM cases, 81 AM cases, 28 MM cases), 27 metastatic-melanoma cases, and 43 nevus cases. Multivariate analysis of age, sex, histological type, etc., proteogenomic and phosphoproteomic analysis, combined with functional experiments utilizing both primary tumor cells derived from patients and in vitro assays revealed that *PRKDC* amplification not only led to increased cognate protein expression, but also strongly associated with the activation of one-carbon metabolism and, in turn, might promote tumor cell proliferation and impact prognosis. Proteome-based stratification of melanomas results in three molecular subtypes, namely, S-I (featuring ECM), S-II (featuring angiogenesis), and S-III (featuring cell proliferation), which show a significant correlation with the clinical outcome. Immune clustering defined three immune clusters across the histological subtypes. Moreover, further analysis combined with an independent anti-PD-1 treatment cohort revealed that activation of the MAPK7-NFKB signaling pathway may facilitate T-cell recruitment to the tumor microenvironment and enhance the sensitivity of patients to immunotherapy; on the other hand, PRKDC may reduce the sensitivity of melanoma patients to immunotherapy by increasing DNA damage and enhancing tumor cell proliferation. Overall, our study provides insight into the potential mechanistic significance of melanoma tumorigenesis and serves as a resource to help decipher biological insight and address unmet clinical needs.

## Results

### Proteogenomic landscape of melanomas

**Fig. 1:**
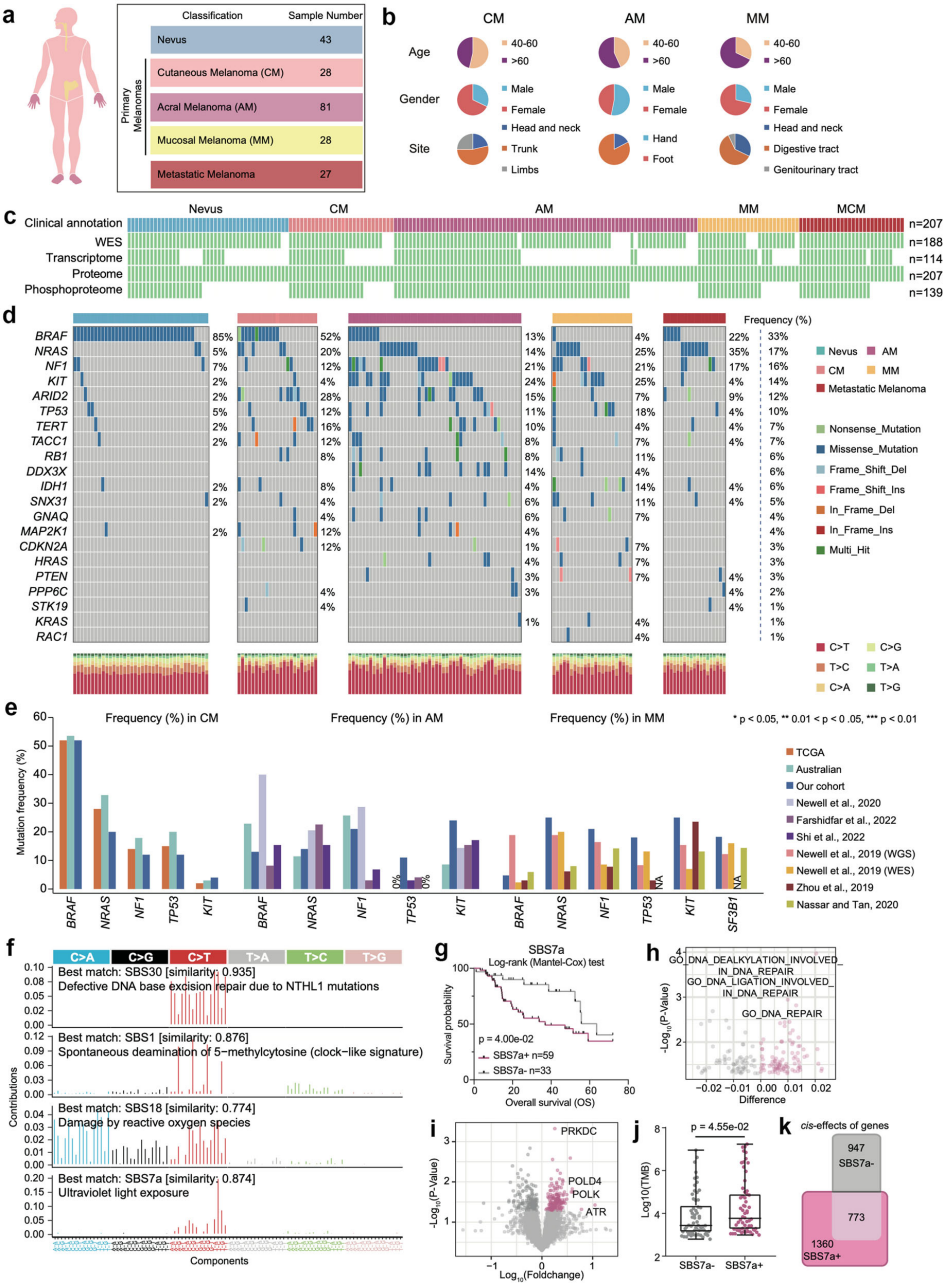
Multi-omics landscape of melanoma samples. **a** Summary of the data and metadata generated in this study. **b** The pie charts of key demographic and histologic features of melanoma patient samples characterized in this study. **c** Schematic of multi-omics analysis of melanomas. A total of 43 nevi, 28 cutaneous melanomas (CM), 81 acral melanomas (AM), 28 mucosal melanomas (MM) samples, and 27 metastatic cutaneous melanomas (MCM) obtained from a cohort of 198 patients are analyzed. All samples are prepared as formalin-fixed paraffin-embedded (FFPE) tissue slides. The white gaps in the schematic represent the missing data. Numbers on the right indicate the samples in each category. **d** Mutation colormap of hot spot mutations of melanomas in our cohort. **e** Bar plots illustrated mutational frequencies for genes with significant differences between the cohort investigated in our study and previously published melanoma studies. **f** The relative mutations mutation frequencies of 96 tri-nucleotide mutation patterns are plotted with SBS30, SBS1, SBS18, and SBS7a mutation patterns in our cohort (*n* = 124) using tool “signer”. **g** Kaplan–Meier curves for overall survival (OS) based on patients with (*n* = 59) and without (*n* = 33) SBS7a mutation signature (log-rank test) in our cohort. **h** The volcano plot showed the elevated protein pathways in patients with SBS7a mutation signature. **i** The volcano plot showed the elevated proteins in patients with SBS7a mutation signature. **j** The boxplot showed the comparison of the tumor mutation burden (TMB) between patients with and without SBS7a mutation signature (Wilcoxon test) in our cohort (*n* = 124). **k** Venn diagram depicted the cascading effects of CNAs in patients with and without SBS7a mutation signature.

To obtain a comprehensive molecular understanding of melanoma, we assembled formalin-fixed paraffin-embedded (FFPE) tissues derived from a cohort of 155 melanoma patients, with 137 primary melanomas (PMs) (CMs: 81 AMs; 28 CMs, 28 primary MMs), and 27 metastatic melanomas. Nine of the 27 metastatic melanomas were matched with their corresponding primary tumor samples. The tumor samples were evaluated by two skilled pathologists to ensure that tumor cells accounted for more than 80% of each tumor region (Materials and methods). Additionally, we incorporated 43 nevus tissues into our cohort as benign controls (Fig. 1a). The neoplastic cellularity (or tumor purity) ranged from 84% to 97% (median 93%) as judged by pathology review (Supplementary Table S1). Neoplastic cellularity was evaluated independently by whole-exome sequencing (WES) using the ABSOLUTE algorithm^16^ (Materials and methods), and ranged from 71% to 90% (median 84%) (Supplementary Table S1). Clinical data, including sex, age at diagnosis, tumor grade, tumor site, and survival, are summarized in Supplementary Table S1 and Fig. 1b. WES was performed for 188 samples (41 nevus samples, 124 PM samples, and 23 metastatic melanoma samples). Transcriptome analysis was performed for 114 samples (nevus samples, *n* = 20; PM samples, *n* = 75; metastatic melanoma samples, *n* = 19). Mass spectrometry (MS)-based proteomic analysis was conducted for all 207 samples (nevus samples, *n* = 43; PM samples, *n* = 137; metastatic melanoma samples). A phosphoproteomic analysis was conducted for 139 samples (nevus samples, *n* = 20; PM samples, *n* = 102; and metastatic melanoma samples, *n* = 17) using the Fe-NTA phosphopeptide enrichment strategy (Fig. 1c).

WES data helped achieve 110-fold mean target coverage, with 93.5% of the bases achieving at least 10-fold coverage. The overall proportions of SNVs were similar to those observed in the TCGA cohort^7,12^, with cytosine to thymine (C > T) transition observed as the most frequently occurring SNV (Fig. 1d). Comparative analysis across studies based on mutational frequencies derived from the TCGA cohort^12^, Australian cohort^7^, and six other studies^17–22^ confirmed the diverse mutational frequencies of hotspot genes among AMs, CMs, and MMs (Fig. 1e). For example, the mutation frequency of *BRAF* is more than 50% in CMs and less than 5% in MMs.

We identified four mutational signatures by “sigminer”^23^ in our melanoma patients, SBS30, SBS1, SBS18, and SBS7a, associated with patients age at diagnosis, UV damage, and DNA damage repair (Fig. 1f) (Materials and methods). To relate the associations of the SBS with clinical features, patients were dichotomized into SBS-positive (SBS^+^) and SBS-negative (SBS^–^) groups based on enrichment scores. Survival analysis revealed that only SBS7a was associated with OS (log-rank test, *p* = 0.04) (Fig. 1g). To confirm whether the same signatures and prognostic associations of SBS7a are robust to the choice of signature derivation tool, we further conducted a mutational signature analysis using three additional methods, “MutationalPatterns”^24^, “Maftools”^25^, and “SigProfilerExtractor”^26^. As a result, the mutational signatures calculated by the three approaches remained similar to the mutational signature characteristics calculated by “sigminer”. Specifically, SBS30, SBS1, SBS18, and SBS7a were also identified as the top 4 mutational signatures in our melanoma cohort (Supplementary Fig. S1a). Moreover, the enrichment scores of SBS30, SBS1, SBS18, and SBS7a in each sample, which were calculated by the four tools, remained similar, with an average Spearman correlation of *r* = 0.90 (*p* < 0.05) (Supplementary Fig. S1b).

Furthermore, all four approaches confirmed that patients who harbored elevated SBS7a enrichment scores had worse prognoses in the TCGA cohort (Fig. 1g and Supplementary Fig. S1c). We further utilized data from the TCGA cohort (*n* = 575) to validate the correlation between SBS7a and patients’ clinical outcomes. We also calculated the mutational signatures using the above four approaches based on WES data from the TCGA cohort. As a result, SBS7a was also the top mutational signature in the TCGA cohort (Supplementary Fig. S1d). Moreover, consistent with our findings, patients with higher SBS7a enrichment scores also showed shorter OS (Supplementary Fig. S1e).

Additionally, we did not observe any difference among the SBS7a enrichment scores of CMs, AMs, and MMs (Supplementary Fig. S2a), implying that this mutation signature is more universal than unique to a specific histological subtype. Further analysis revealed that patients in the SBS7a^+^ group had a greater mutational frequency of *NF1* and consequently lower protein expression of NF1 (Wilcoxon test, *p* < 0.05) (Supplementary Fig. S2b, c). In concordant with our findings, previous research conducted by Helena et al. reported that SBS7a is related to *NF1* mutations^27^. To explore the molecular features of the SBS7a^+^ group, we conducted a comparative analysis of pathway enrichment scores (GSVA scores based on proteomic data) (Materials and methods) and protein expression data between the SBS7a^+^ and SBS7a^–^ groups. As a result, the DNA repair pathway was significantly enriched in the SBS7a^+^ group, and proteins such as PRKDC, POLD4, POLK, and ATR were dominantly overrepresented in the SBS7a^+^ group (Fig. 1h, i). Consistently, the samples belonging to the SBS7a^+^ group showed significantly higher TMB (Wilcoxon test, *p* = 4.55e−02) (Fig. 1j). Intriguingly, correlation analysis among copy number alterations (CNAs), transcriptome and proteome data indicated that the samples belonging to the SBS7a^+^ group presented more *cis*-effect events (Spearman’s correlation, *p* < 0.05; Materials and methods) than did the samples belonging to the SBS7a^–^ group, suggesting that CNAs more profoundly impacted their cognate transcriptome and proteome in the SBS7a^+^ group (Fig. 1k).

Whole-cell extracts of HEK293T cells were used as quality control (QC) samples for MS. Analysis of this extract revealed the robustness and consistency of the mass spectrometer, which was evidenced by considering a high Spearman’s correlation coefficient (0.88–0.92) between the proteomes of the QC samples (Materials and methods) (Supplementary Fig. S2d). For proteomic analysis, 11,206 proteins were identified (1% false discovery rate (FDR) at the peptide and protein levels), with 7000 proteins per sample on average (Supplementary Fig. S2e). To analyze and compare the dynamic range of the melanoma and nevus proteomes, we used the protein abundance (Materials and methods). The melanoma and nevus proteomes are highly dynamic, spanning more than seven orders of magnitude (Supplementary Fig. S2f). Correlation analysis of paired transcriptomic and proteomic data revealed that 98.19% of the 4429 mRNA‒protein pairs detected in all the samples were positively correlated (Supplementary Fig. S2g) (Materials and methods), and 1.81% were negatively correlated. The median Spearman correlation coefficient between 4429 mRNA‒protein pairs was 0.4, which was similar to that reported in other studies^28,29^ (Supplementary Table S1). These strongly positively correlated mRNAs and proteins were enriched in the DNA replication, ECM‒receptor interaction, mismatch repair, and oxidative phosphorylation pathways (Supplementary Fig. S2g). A total of 25,318 phosphosites over 4922 phosphoproteins were used for phosphoproteomic analysis. In concordantly, 11,832 phosphosites were detected for each sample. The phosphorylation of some well-known cancer driver genes, including *RB1* at T373, *CDK1* at T161, and *MCM* at S27, has been identified exclusively in tumors. We further verified the enrichment of these proteins in melanoma tissues by immunohistochemistry (IHC) with phosphorylation antibodies (Supplementary Fig. S2h). In general, our study portrayed the systematic molecular features of melanomas at the multi-omics level (genomic, transcriptomic, proteomic, and phosphoproteomic levels).

### Integrative proteogenomic analyses reveal functional consequences of mutations and CNAs

**Fig. 2:**
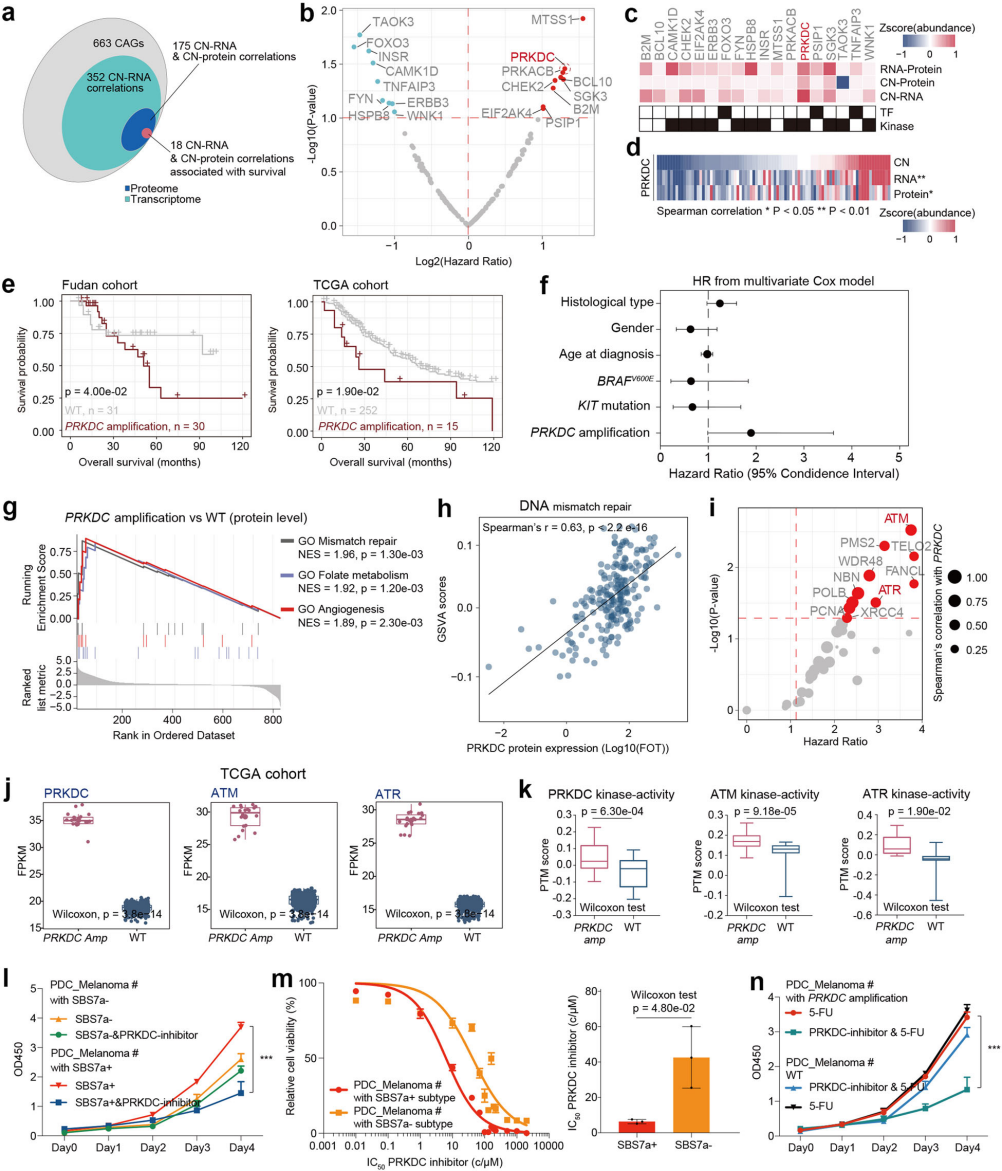
Integrative omics analyses of melanoma samples. **a** Venn diagram depicted the cascading effects of CNAs of cancer-associated genes (CAGs) in melanomas. The overlap between significant *cis* events across transcriptome and proteome are shown. **b** The volcano plot showed the copy number alteration predictive of OS in melanomas. **c** The heatmap represented the 18 survival associated CAGs which show significant correlation with copy numbers (CNs), mRNAs, or proteins. **d** The heatmap showed the correlation between CNs, mRNAs, and proteins of PRKDC. Samples are ranked from lowest (left) to highest (right) copy number values of *PRKDC*. **e** Kaplan–Meier curves for OS based on patients’ CNV status (log-rank test) in our cohort and TCGA cohort. **f** The forest plot showed the 95% CI of hazard ratio coefficients from Cox-regression for *PRKDC* amplification, and other covariates based on our cohort. **g** GSEA plots for DNA mismatch repair related pathways in *PRKDC* amplification vs WT comparisons. **h** Spearman-rank correlation of the PRKDC protein expression, and GSVA scores of DNA repair process. **i** The volcano plot showed the expression of proteins correlated with *PRKDC* copy numbers and predictive of OS in melanomas. **j** The comparison of the mRNA expression of ATM, and PRKDC between patients harboring *PRKDC* amplicons and WT samples in TCGA cohort (*n* = 267) (Wilcoxon rank test). **k** The comparison of the kinase activity of ATM, and PRKDC between patients harboring *PRKDC* amplicons and WT samples in our cohort (*n* = 96) (Wilcoxon rank test). **l** Dose–response curves of PRKDC inhibitor were determined on day 2 after inhibitors adding in PDCs from melanoma patients with or without SBS7a mutation signature. The data represent the mean values ± SD (*n* = 3) (left). The volcano plot showing the half-maximal inhibitory concentration (IC_50_) scores. The data represent the mean values ± SD (*n* = 3) (right). **m** Proliferation of the PDCs from melanoma patients with and without SBS7a mutation signature based on the use of PRKDC inhibitor or control (two-way ANOVA followed by Tukey’s multiple comparison test). The data are presented as mean ± SEM, *p < 0.05; **p < 0.01; ***p < 0.001. **n** Proliferation of the PDCs from melanoma patients with or without PRKDC amplification based on the use of PRKDC inhibitor and 5-fluorouracil (5-FU) or control (two-way ANOVA followed by Tukey’s multiple comparison test). The data are presented as mean ± SEM, *p < 0.05; **p < 0.01; ***p < 0.001.

Genes’ CNAs are strongly associated with clinical outcomes^30^. To identify functionally important genes within CNA regions in our melanoma cohort, we focused on 663 cancer-associated genes (CAGs) (Materials and methods). A total of 163 significantly positive correlations were observed for both RNA and proteins, with 18 CAGs, including 13 kinases (*PRKACB*, *TAOK3*, *PRKDC*, etc.), and 3 transcription factors (TFs) (*PSIP1*, *TNFAIP3*, and *FOXO3*), showing a strong association with patient survival (Fig. 2a–c and Supplementary Table S2). Among these 18 CAGs, *MTSS1* and *PRKDC* were the top 2 genes significantly associated with poor patient prognosis, and *PRKDC* encodes the kinase DNA-dependent protein kinase catalytic subunit (DNA-PKcs), which was significantly positively correlated with the expression of its cognate RNA and protein but not with that of MTSS1 in our cohort or TCGA cohort (Fig. 2d and Supplementary Fig. S3a). Further survival analysis based on both our cohort and the TCGA cohort^31^ indicated that patients who harbored *PRKDC* amplification had a worse prognosis (Fig. 2e). We also found that the amplification rates of *PRKDC* were significantly higher in AMs and MMs than in CMs (49% in AMs, 63% in MMs and 18% in CMs) and were confirmed to be associated with patient clinical outcomes in all three histological types of melanomas, consistent with previous studies^12,32,33^ (Supplementary Fig. S3b, c). We also evaluated the overlap between SBS7a^+^ patients and *PRKDC* amplification patients. Among 59 patients who harbored SBS7a^+^ mutational signatures, 33 had *PRKDC* amplifications. By conducting a prognostic evaluation, we found that patients who had both SBS7a^+^ mutational signatures and *PRKDC* amplification had a worse prognosis (Supplementary Fig. S3d). This result indicated that *PRKDC* amplification combined with SBS7a mutation signature might further enhance tumor malignancy. We further conducted multivariate Cox regression analysis of the baseline data of our cohort, including age, sex, clinical variables such as histological type, pathological subtype, tumor site, Clark level, ulcer, and our prognostically relevant findings, including the SBS7a^+^ mutational signature and *PRKDC* amplification status. As a result, *PRKDC* amplification was the most significant predictive factor for the prognosis of melanoma patients (Fig. 2f and Supplementary Table S2).

We then focused on the functional impact of the amplification of *PRKDC* on both its cognate protein and other proteins. Integrative analysis revealed that in addition to upregulating the expression of its cognate mRNA and protein (Spearman’s *r* > 0.3, *p* < 0.05), the amplification of *PRKDC* increased the expression of proteins involved in DNA repair, DNA mismatch repair, and the cellular response to DNA damage stimuli (Fig. 2g). Functionally, PRKDC is a core regulator of DNA double-strand break repair^34^ and has been reported to be a promising drug target for breast cancer^35^ and medulloblastoma^36^. Along with these studies, we found that the protein expression of PRKDC was significantly correlated with the GSVA score of the nonhomologous joining DNA repair process (Spearman’s *r* = 0.63, *p* < 0.0001), and the expression of proteins involved in DNA damage repair, including ATM, ATR, CUL4B, XRCC4, XRCC1, and XPA, was also highly correlated with the protein expression of PRKDC (Spearman’s *r* > 0.2, *p* < 0.05) (Fig. 2h, i). Survival analysis indicated that 12 DNA damage-related proteins, including 2 kinases (ATM and ATR), were associated with poor survival (hazard ratio > 1, *p* < 0.05) (Fig. 2i). Moreover, the mRNA expression (from the TCGA cohort) and inferred kinase activity (from our cohort) of ATM, ATR and PRKDC (Materials and methods) were significantly upregulated in patients harboring *PRKDC* amplification, suggesting that PRKDC might collaborate with ATM and ATR to upregulate DNA damage repair and drive poor prognosis (Fig. 2j, k). Collectively, our data suggested that the amplification of *PRKDC* was associated with the upregulation of DNA damage repair and might further impact patient prognosis through collaboration with ATM and ATR. We also further illustrated the potential role of MTSS1 in melanomas. We conducted correlation analysis and found that the protein expression of MTSS1 was positively correlated with the cortical actin cytoskeleton organization pathway (Supplementary Fig. S3e). The expression of proteins, including PARVA, ABL1, MINK1, and HCLS1, was positively correlated with that of MTSS1 (Supplementary Fig. S3f). Previous research has indicated that elevated actin cytoskeleton organization might lead to tumor cell migration and tumor metastasis^37^. Thus, our findings emphasized that MTSS1 might impact melanoma prognosis by regulating the actin cytoskeleton organization pathway.

Previous research has reported that PRKDC participated in nonhomologous end joining (NHEJ) of DNA double-strand breaks (DSBs)^38^, and its abnormal expression is associated with chemotherapy resistance^39,40^. To illustrate whether the elevated PRKDC protein expression impacts the response of melanomas to chemotherapy, we surveyed the sensitivities of twelve FDA-approved cancer drugs that functioned in blocking DNA synthesis, including 5-FU, Temozolomide (TMZ), Etoposide, Voxlaisib, cisplatin, Oxaliplatin, etc. using published cell line perturbation data from GDSC (https://www.cancerrxgene.org/). As a result, the sensitivity to 5-FU showed the most significant positive correlation with the protein expression of PRKDC (5-FU: Spearman’s *r* = 0.77, *p* = 0.026), suggesting that melanoma patients who harbor *PRKDC* amplicons might be more sensitive to 5-FU treatment (Supplementary Fig. S3g). These results indicated that enhanced PRKDC expression might inhibit the efficacy of 5-FU treatment. To further investigate whether 5-FU could more efficiently inhibit the proliferation of tumor cells with elevated PRDKC expression, we constructed a stable PRKDC-overexpressing HMCB cell line (PRKDC-OE-HMCB) using the pCDH-PRKDC-copGFP vector and knocked down *PRKDC* (PRKDC-KD-HMCB) utilizing pLKO.1-CMV-shPRKDC-copGFP. RT‒PCR analysis was utilized to verify the expression of PRKDC in the PRKDC-OE-HMCB and PRKDC-KD-HMCB strains. The results revealed significantly elevated expression of PRKDC in the PRKDC-OE-HMCB group and significantly decreased expression of PRKDC in the PRKDC-KD-HMCB group (Supplementary Fig. S3h). Furthermore, tumor cells (OE-Control-HMCB, PRKDC-OE-HMCB, sh-Control-HMCB, and PRKDC-KD-HMCB) were treated with 5-FU. As a result, compared to OE-Control-HMCB, PRKDC-OE-HMCB was more sensitive to 5-FU; in contrast, the sensitivity of PRKDC-KD-HMCB to 5-FU was significantly lower than that of sh-Control-HMCB (Supplementary Fig. S3i). These results confirmed our hypothesis that cells with elevated PRKDC expression are more sensitive to 5-FU, as indicated by a lower IC_50_ (Supplementary Fig. S3i). Based on these findings, we evaluated the proliferation rates of the OE-Control-HMCB- and PRKDC-OE-HMCB-treated cells treated with 5-FU or left untreated. The results revealed that in the 5-FU-untreated group, compared to the OE-Control-HMCB group, the PRKDC-OE-HMCB group exhibited significantly elevated cell proliferation rates (Supplementary Fig. S3j). On the contrary, in the 5-FU-treated group, compared to the OE-Control-HMCB group, the PRKDC-OE-HMCB group showed no significant increase in proliferation tendency (Supplementary Fig. S3k). Moreover, compared with those in the 5-FU-untreated PRKDC-OE-HMCB group, the proliferation rates in the PRKDC-OE-HMCB group treated with 5-FU were significantly lower (Supplementary Fig. S3j, k).

Notably, since PRKDC was also one of the most dominantly expressed proteins in the SBS7a^+^ group, we evaluated the clinical relevance of targeting PRKDC in SBS7a^+^ patients. Therefore, we collected primary tumor cell cultures (PDCs) from SBS7a^+^ and SBS7a^–^ patients (Melanoma #8, Melanoma #14: SBS7a^+^ patients; Melanoma #9, Melanoma #19: SBS7a^–^ patients) (Materials and methods) and evaluated the response of PDCs to PRKDC inhibitor (NU7441). As a result, PRKDC inhibitor significantly decreased the proliferation of PDCs collected from SBS7a^+^ patients but had no significant impact on the growth of PDCs collected from SBS7a^–^ patients (Fig. 2l).

Consistent with this finding, we observed that PDCs from SBS7a^+^ patients were also more sensitive to PRKDC inhibitor, with significantly lower IC_50_ values (median IC_50_: 6.287 μM in SBS7a^+^ vs 42.02 μM in SBS7a^–^) (Fig. 2m). Currently, although chemotherapy drugs including 5-fluorouracil (5-FU), its efficiency alone is poor, and it is accompanied by side effects. We then investigated whether PRKDC inhibitor could be served as a complement to 5-FU for the treatment of SBS7a^+^ patients. We compared the cell proliferation rates among PDCs collected from SBS7a^+^ and SBS7a^+^ patients treated with 5-FU alone or treated with both 5-FU and PRKDC inhibitor. As a result, the proliferation rates of PDCs from SBS7a^+^ patients were significantly lower after combination treatment with the PRKDC inhibitor and 5-FU than after treatment with 5-FU alone. However, for PDCs from SBS7a^–^ patients, the combination treatment strategy did not improve cell growth inhibition efficiency compared with 5-FU treatment alone (Fig. 2n). These results suggested that SBS7a^+^ patients might benefit from combination treatment with PRKDC inhibitor and 5-FU.

### Aberrant folate metabolism balance contributes to tumor development in primary melanomas

**Fig. 3:**
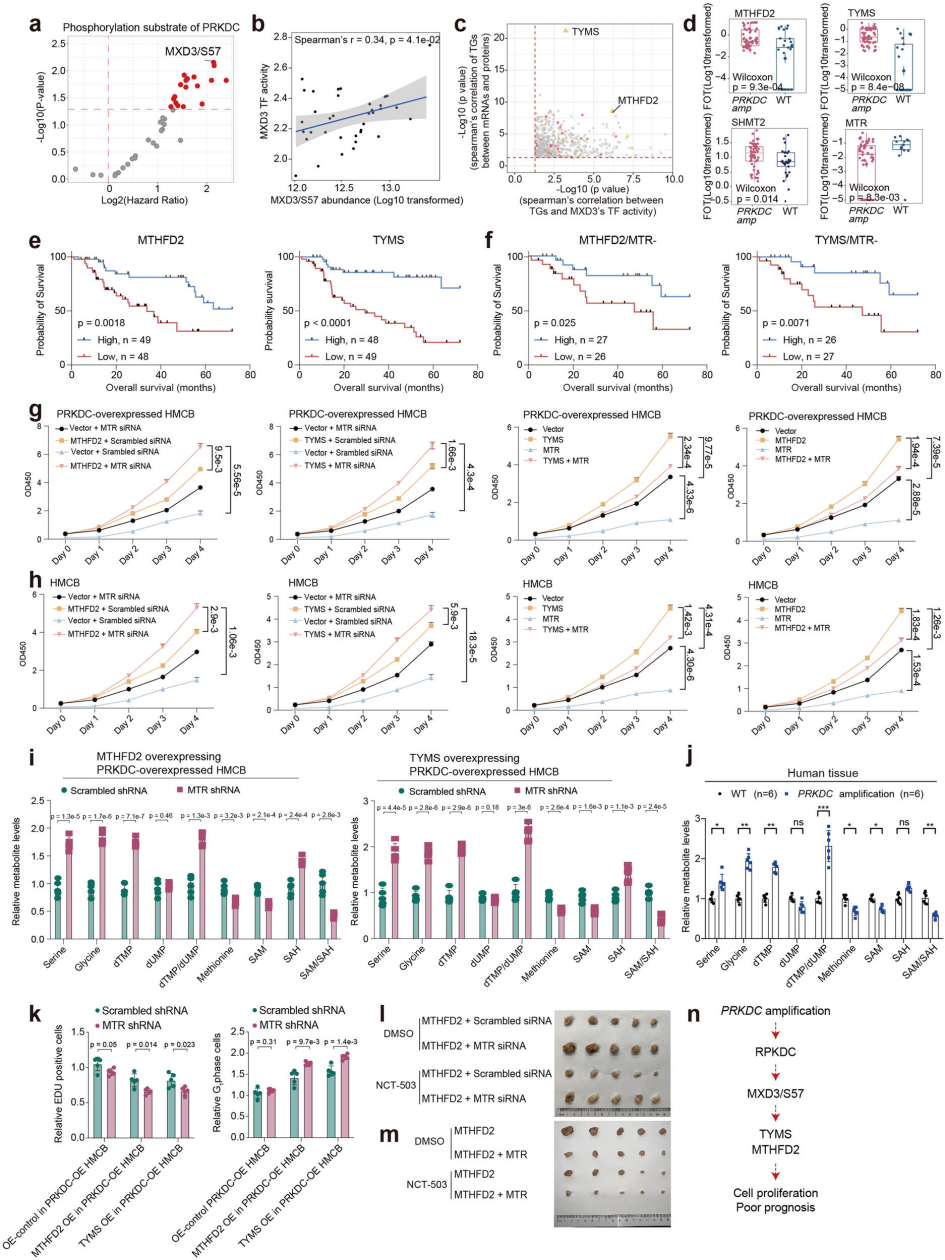
Aberrant folate metabolism balance contributes to the tumor development in melanomas. **a** The volcano plot showed the abundance of phosphorylation substrate of PRKDC predictive of OS in melanomas. **b** Spearman-rank correlation of the MXD3’s TF activity, and MXD3/S57’s abundance in melanomas. **c** Spearman-rank correlation of the MXD3’s TF activity, and MXD3’s TGs protein expression (*x*-axis); Spearman-rank correlation of the mRNA expression, and protein expression of MXD3’s TGs (*y*-axis). **d** The boxplot showed the expression of MTHFD2, TYMS, SHMT2, and MTR in patients harboring *PRKDC* amplicons and WT samples in our cohort (*n* = 124) (Wilcoxon rank test). **e** Kaplan–Meier curves of OS in relation to MTHFD2 and TYMS expression levels in PMs. **f** Kaplan–Meier curves of TYMS and MTHFD2 based on the MTR low expression. The log-rank test is performed for survival analysis. **g** Proliferation of the indicated HMCB cells when MTHFD2/TYMS or an empty vector was overexpressed based on the use of MTR knockdown or control (two-way ANOVA followed by Tukey’s multiple comparison test). The data are presented as mean ± SEM, *p < 0.05; **p < 0.01; ***p < 0.001. **h** Proliferation of HMCB cells after MTR overexpression based on MTHFD2/TYMS overexpression (two-way ANOVA followed by Tukey’s multiple comparisons test). The data are presented as mean ± SEM, *p < 0.05; **p < 0.01; ***p < 0.001. **i** Metabolism of indicated HMCB cells after MTR KO based on MTHFD2 or TYMS overexpression (*t*-test). Data are represented as mean ± SEM, *p < 0.05; **p < 0.01; ***p < 0.001. **j** Metabolism of in melanoma tissues from patients harboring *PRKDC* amplicons and WT samples in our cohort (two-way ANOVA followed by Tukey’s multiple comparisons test). Data are represented as mean ± SEM, *p < 0.05; **p < 0.01; ***p < 0.001. **k** DNA synthesis of indicated HMCB cells after MTR KO on MTHFD2 or TYMS overexpression (*t*-test) (left). Amount of G1 phage of indicated HMCB cells after MTR KO on MTHFD2 or TYMS overexpression (*t-*test) (right). Data are represented as mean ± SEM, *p < 0.05; **p < 0.01; ***p < 0.001. **l** Xenograft tumor images indicated that A375 cells were subcutaneously injected into nude mice based on MTR loss and MTHFD2 overexpression. **m** Xenograft tumor images indicated that A375 cells were subcutaneously injected into nude mice based on MTR overexpression and MTHFD2 overexpression. **n** Illustration of the activation of PRKDC–MXD3 signaling pathway combined with one-carbon unit enrichment led to tumor growth in melanomas.

PRKDC is an important DNA-PKs^38^. To identify prognosis-related substrates of PRKDC, we conducted survival analysis on the phospho-substrates that were positively correlated with the expression of PRKDC. As a result, the S57 phosphorylation site of MXD3 was subsequently screened out because it was the top-ranked phospho-substrate of PRKDC associated with OS (Fig. 3a). As a member of the MXD family, the TF MXD3 plays a key role in cell cycle progression and cell proliferation^41^. We then inferred the MXD3 TF activity based on the mRNA expression of its target genes (TGs) using GSVA algorithm (Materials and methods). As expected, the inferred TF activity of MXD3 was strongly correlated with the abundance of MXD3/S57 (Fig. 3b). To gain insight into the mechanism of how MXD3’s TF activity led to poor prognosis, we performed correlation analysis and observed that TYMS and MTHFD2 were the top two TGs of MXD3 whose mRNA expression was strongly associated with the MXD3’s TF activity and their cognate proteins’ expression (Fig. 3c). Consistently, the protein expression of MTHFD2 and TYMS showed elevated expression in samples that harbored *PRKDC* amplification (Fig. 3d).

We conducted a series of functional experiments to further investigate the associations among *PRKDC* amplification, folate metabolism, and patient prognosis. We first constructed a stable PRKDC-overexpressing HMCB cell line (PRKDC-OE-HMCB) using the pCDH-PRKDC-copGFP vector and knocked down *PRKDC* (PRKDC-KD-HMCB) utilizing pLKO.1-CMV-shPRKDC-copGFP (Supplementary Fig. S4a). The results confirmed the significantly elevated expression of PRKDC in the PRKDC-OE-HMCB group and the significantly decreased expression of PRKDC in the PRKDC-KD-HMCB group (Supplementary Fig. S4b). We then evaluated the phosphorylation of MXD3 in PRKDC-overexpressing and KD HMCB cell lines by phosphoproteomic analysis. The results confirmed that overexpression of PRKDC significantly increased the phosphorylation of MXD3 and that PRKDC knockdown decreased the phosphorylation of MXD3 (Supplementary Fig. S4c). These findings confirmed the regulatory effect of PRKDC on the phosphorylation of MXD3. To verify the role of MXD3 in regulating the downstream expression of TYMS and MTHFD2, we constructed a stable MXD3-overexpressing HMCB cell line and performed ChIP‒qPCR. As a result, the overexpression of MXD3 significantly elevated the transcription of its target genes TYMS and MTHFD2 (Supplementary Fig. S4d). These results confirmed the regulatory role of MXD3 in regulating the expression of TYMS and MTHFD2.

Functionally, both TYMS and MTHFD2 participate in the folate cycle, which is an important branch of folate metabolism that can provide one-carbon units for purine and thymidine synthesis^42,43^. To further illustrate the impact of the folate cycle on tumor development, we screened the expression of enzymes involved in folate metabolism. In addition to MTHFD2, TYMS, SHMT2, a key mitochondrial enzyme involved in serine catabolism that converts serine to glycine and a one-carbon unit^44^, exhibited significantly increased expression in patients with *PRKDC* amplification (Fig. 3d). The expression of MTR, which functions as a unique metabolic linker of the folate and methionine cycles in folate metabolism^45^, was significantly decreased in patients with *PRKDC* amplification (Wilcoxon test, *p* < 0.0001). These findings were further confirmed by western blotting assay (Supplementary Fig. S4e). We then profiled the proteomes of four types of melanoma cells with different PRKDC expression patterns, namely, Vector-OE-high (OE-Control-HMCB), PRKDC-OE-HMCB, Scramble-shRNA-HMCB (sh-Control-HMCB), and PRKDC-KD-HMCB. Comparative analyses were also conducted. Proteins enriched in the folate metabolism pathway, including key enzymes such as SHMT2, MTHFD2, and GART, were significantly elevated in the PRKDC-OE-HMCB group compared to the OE-Control-HMCB group, whereas these proteins were significantly downregulated in the PRKDC-KD-HMCB group compared to the sh-Control-HMCB group (Supplementary Fig. S4f). In contrast, the protein MTR, which functions as a unique metabolic linker of the folate and methionine cycles in folate metabolism^45^, showed decreased expression in the PRKDC-OE-HMCB group compared to the OE-Control-HMCB group, while it showed increased expression in the PRKDC-KD-HMCB group compared to the sh-Control-HMCB group (Supplementary Fig. S4g). In summary, these results confirmed that alterations in PRKDC expression impacted the expression of key enzymes involved in folate metabolism.

Since we found that *PRKDC* copy number alterations were associated with poor patient prognosis and confirmed that PRKDC expression alterations could impact the expression of proteins involved in folate metabolism, we next wanted to determine whether alterations in the expression of proteins involved in folate metabolism are responsible for the poor prognosis resulting from *PRKDC* copy number alterations. Consistent with our hypothesis, survival analysis indicated that MTHFD2 and TYMS were associated with poor prognosis (Fig. 3e). Although MTR did not exhibit a strong correlation with survival, it exerted a strong synergistic effect in combination with MTHFD2 and TYMS. MTHFD2/TYMS expression was associated with worse survival in the MTR low-expression group than in the MTR high-expression group (Fig. 3f).

To further illustrate how the elevated expression of MTHFD2 and TYMS and the downregulation of MTR are associated with patients’ unfavorable clinical outcomes, we constructed an MTHFD2-overexpressing plasmid, a TYMS-overexpressing plasmid, and an MTR overexpressed plasmid utilizing a pCDH-copGFP vector (pCDH-MTHFD2-copGFP, pCDH-TYMS-copGFP, pCDH-MTR-copGFP). Moreover, shRNAs for MTHFD2, TYMS, and MTR were also constructed using the pLKO.1-CMV-copGFP vector (pLKO.1-CMV-shMTHFD2-copGFP, pLKO.1-CMV-shTYMS-copGFP and pLKO.1-CMV-shMTR-copGFP). Then, the vectors were transfected into the HMCB and A375 cell lines. We utilized the CCK-8 assay to investigate how alterations in MTHFD2, TYMS, and MTR affect tumor cell growth. As a result, the overexpression of MTHFD2 or TYMS significantly enhanced the tumor cell proliferation rate (Fig. 3g and Supplementary Fig. S5a), whereas the tumor cell growth promoted by MTHFD2 or TYMS overexpression was decreased by the overexpression of MTR (Fig. 3g and Supplementary Fig. S5a). We further evaluated the impacts of MTHFD2, TYMS, and MTR on tumor cell proliferation under PRKDC overexpression conditions. For this purpose, we transfected the MTHFD2, TYMS, and MTR overexpression or knockdown vectors into the PRKDC-overexpressing HMCB cell line (PRKDC-OE-HMCB), respectively, and exanimated the tumor cell proliferation rates. The results revealed that compared to HMCB with MTHFD2-OE or TYMS-OE, PRKDC-OE cells that overexpressed with MTHFD2 or TYMS showed significantly elevated tumor cell proliferation (Fig. 3h and Supplementary Fig. S5b). These results indicated the controversial functions of MTR with both MTHFD2-OE and TYMS-OE in promoting tumor cell proliferation and emphasized that the impact of TYMS and MTHFD2 on enhancing tumor cell proliferation could be further improved in PRKDC-OE-HMCB.

The methionine cycle, which utilizes one-carbon units for methylation, competes with the folate cycle, which utilizes one-carbon units derived from serine for DNA synthesis. Therefore, we hypothesized that the loss of MTR might uncouple the link between the folate cycle and the methionine cycle and lead to one-carbon unit enrichment in the DNA synthesis pool. Consistent with this hypothesis, we found that knockdown of MTR in cells resulted in increased production of folate cycle metabolites such as serine, glycine, and dTMP and increased dTMP-to-dUMP ratios (Fig. 3i and Supplementary Fig. S5c). In contrast, the methionine cycle in MTR knockdown cells was impaired, and the production of methionine and SAM and the SAM/SAH ratio decreased (Fig. 3i and Supplementary Fig. S5c). This hypothesis was also verified by the increased serine, glycine, dTMP, and dTMP/dUMP ratios and decreased methionine and SAM levels and SAM/SAH ratios in the melanoma tissues (Fig. 3j). A subsequent stable isotope labeling approach further confirmed that knockdown of MTR enhanced the one-carbon unit flux of the folate cycle and inhibited the one-carbon unit flux of DNA methylation in the methionine cycle (Supplementary Fig. S5d). Moreover, in line with our assumption that the loss of MTR could lead to the enrichment of one-carbon units in DNA synthesis, we found that by knocking down MTR, the DNA synthesis rate was significantly increased, and the number of G1-phase cells was particularly significantly decreased in MTHFD2- or TYMS-overexpressing cells (Fig. 3k and Supplementary Fig. S5f). Since the metabolite serine is the one-carbon resource for the folate cycle^42,43^, we further hypothesized that blockade of serine metabolism may diminish the oncogenic effects of folate cycle metabolism. In line with this assumption, by using a xenograft tumor model, we found that the xenograft tumor growth induced by MTR loss and MTHFD2 overexpression could be abrogated by administering the serine metabolism inhibitor NCT503 (which targets the key serine metabolism enzyme PHGDH^46^) (Fig. 3l, m). Taken together, our data indicated that MTR loss combined with MTHFD2 upregulation led to one-carbon unit enrichment and contributed to PRKDC-MXD3/S57-induced melanoma tumor growth (Fig. 3n).

### Proteomic subtypes of melanomas

**Fig. 4:**
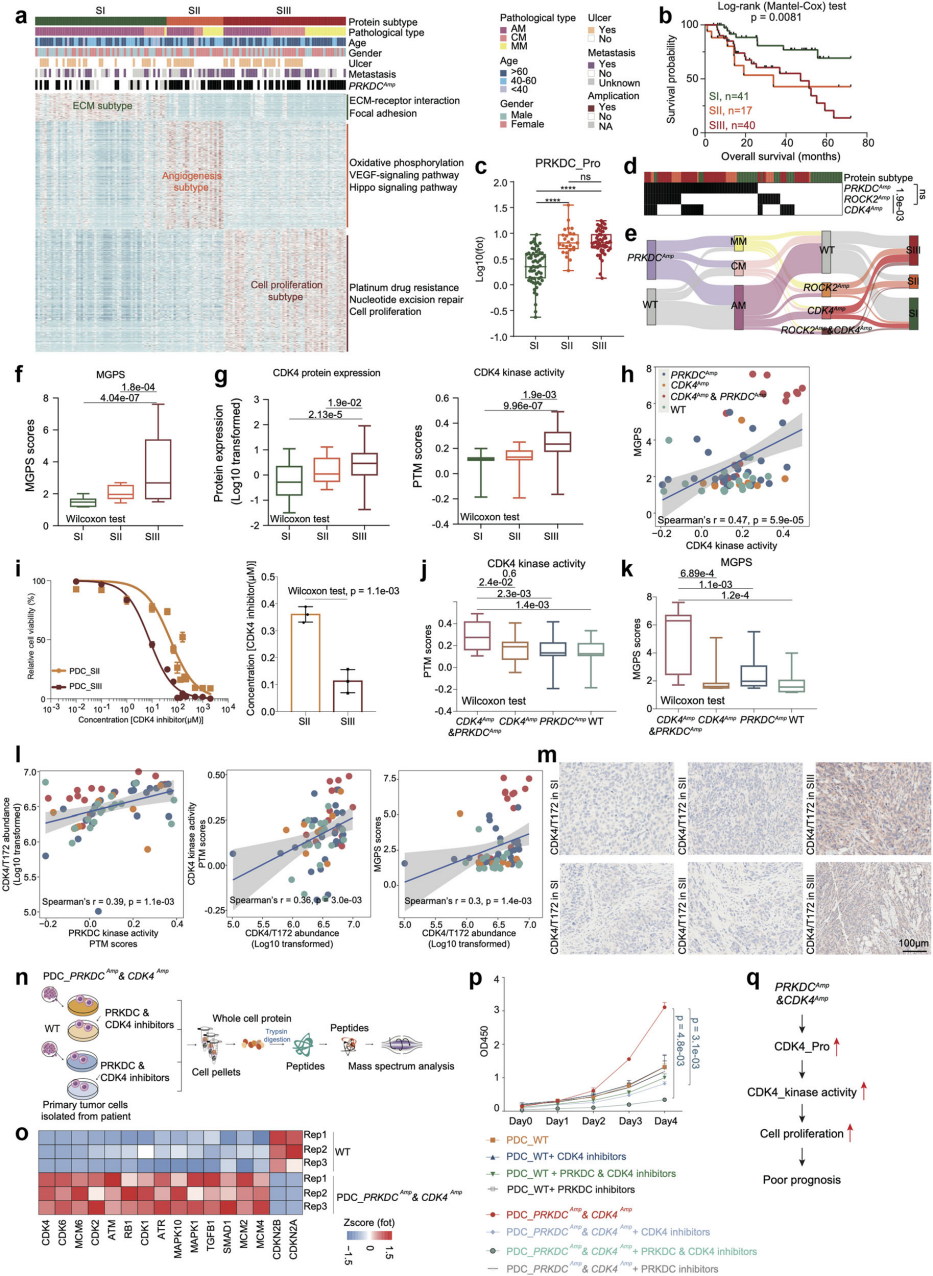
Proteomic subtypes of primary melanomas. **a** Heatmap illustrated clinical information, and frequency of *PRKDC* amplicons in 137 melanoma patients. The remaining section illustrates global proteomic features upregulated in the three proteomic subtypes. The pathways enriched by proteins elevated in corresponding subgroups are labeled on the right. **b** The association of three proteomic subtypes with clinical outcomes in melanoma patients (SI: *n* = 41; SII: *n* = 17; SIII: *n* = 40) (*p* value based on the log-rank test). **c** The boxplot showed the protein expression of PRKDC in the three proteomic subtypes (*n* = 137) (Wilcoxon rank test, ****p < 0.0001). **d** Heatmap illustrated the amplification frequency of *PRKDC*, *CDK4*, *ROCK2* in the three proteomic subtypes (Fisher’s exact test). **e** Sankey plot showed the amplification frequency of *PRKDC*, *CDK4*, and *ROCK2* in the three pathological subtypes and three proteomic subtypes of melanomas. **f** The boxplot showed the MGPS score in the three proteomic subtypes (*n* = 137) (Wilcoxon rank test). **g** The boxplot showed the protein expression and kinase activity of CDK4 in the three proteomic subtypes (*n* = 137) (Wilcoxon rank test). **h** Spearman-rank correlation of the CDK4’s kinase activity and MGPS score in melanomas (*n* = 96). **i** Dose–response curves of CDK4 inhibitor were determined on day 2 after inhibitors adding in PDCs from melanoma patients of SII and SIII proteomic subtypes. The data represent the mean values ± SD (*n* = 3) (left); IC_50_ values of CDK4 inhibitor were determined on day 2 after inhibitors adding. The data represent the mean values ± SD (*n* = 3) (right). **j** The boxplot showed the PTM score of CDK4 in patients harboring *CDK4* amplicons & *PRKDC* amplicons, or only *PRKDC* amplicons, or only *CDK4* amplicons and WT samples in our cohort (*n* = 96) (Wilcoxon rank test). **k** The boxplot showed the MGPS score in patients harboring *CDK4* amplicons & *PRKDC* amplicons, or only *PRKDC* amplicons, or only *CDK4* amplicons and WT samples in our cohort (*n* = 124) (Wilcoxon rank test). **l** Spearman-rank correlation of the PRKDC’s kinase activity and CDK4/T172’s abundance in melanomas (left); Spearman-rank correlation of the CDK4/T172’s abundance and CDK4’s kinase activity in melanomas (middle); Spearman-rank correlation of the CDK4/T172’s abundance and MGPS score in melanomas (right). **m** Immunohistochemistry of CDK4/T172 in SII and SIII proteomic subtype samples, scale bar = 100 μm. **n** The workflow showed the sample collection for mass spectrum analysis. **o** Heatmap illustrated the protein expression of CDK4, CDK6, et al. participating in cell cycle were upregulated in the PDCs from melanoma patients harboring *CDK4* amplicons & *PRKDC* amplicons. **p** Proliferation of the PDCs from melanoma patients with or without PRKDC amplification and CDK4 amplification based on the use of PRKDC inhibitor and CDK4 inhibitor, or only CDK4 inhibitor, or only PRKDC inhibitor, or control (two-way ANOVA followed by Tukey’s multiple comparison test). The data are presented as mean ± SEM. **q** Illustration of the activation of PRKDC–CDK4 signaling pathway combined with cell proliferation led to poor prognosis in melanomas.

Given the intertumoral heterogeneity, it is important to perform molecular subtyping. Since the proteomic data directly reflect cell functions, we performed consensus clustering^47^ based on protein expression ranks in the 137 melanomas; subsequently, we identified three subgroups (S-I, S-II, and S-III) (Fig. 4a and Supplementary Fig. S6a, b and Table S3) (Materials and methods). Remarkably, survival analysis revealed that proteomic subgroups significantly differed in terms of OS (log-rank test, *p* = 0.0081) (Fig. 4b). The evaluation of the clinical features of the proteomic subtypes revealed that S-I patients had a significantly longer OS, and the S-II subgroup exhibited a greater probability of metastasis than did subgroups S-I and S-III (27% in S-I, 66% in S-II, and 38% in S-III) (Supplementary Fig. S6c).

Subgroup-specific pathway enrichment analysis revealed different features among the three proteomic subgroups. Among the three subgroups, S-I was characterized by the regulation of ECM‒receptor interactions and focal adhesion (referred to as the ECM subtype) (Fisher’s exact test, *p* < 0.05). Proteins such as LAMA3, LAMB3, and COL3A1 were dominantly expressed in the S-I subtype. Moreover, S-II was featured with VEGF signaling pathway, epithelial cell signaling pathway and Hippo signaling pathway (referred to as the angiogenesis subtype). Consistently, proteins, including PAK1, RAC1, and PLCG1, were overrepresented in this subtype. Moreover, S-III was enriched in nucleotide excision repair and carbon metabolism (referred to as the cell proliferation subtype). The expression of key regulators of cell proliferation, such as CDK4, were increased in this subtype (Fig. 4a and Supplementary Table S3).

To evaluate the robustness of our proteomic subtyping, we further utilized the proteomic signatures of our proteomic subtyping and performed consensus clustering for the tumor samples from Kabbarah et al.’s cohort^48^ and the TCGA melanoma cohort^12^. The investigation resulted in the stratification of three subgroups in each validation cohort. Using subgroup-specific pathway enrichment analysis, we observed similarities in the molecular characteristics of subgroups (S-I: ECM; S-II: angiogenesis; and S-III: cell proliferation) between the validation cohort and our cohort (Supplementary Fig. S6d, e). Survival analysis based on the TCGA cohort also indicated that patients in the SII and SIII subgroups had worse prognoses than patients in the SI subgroup, which was consistent with our findings (Supplementary Fig. S6f).

Notably, since we observed that both the S-II and S-III subgroups were associated with poor prognosis, we evaluated the expression of PRKDC (an independent prognostic molecule of melanoma (Fig. 2e)) among the three proteomic subtypes. Both S-II and S-III showed increased expression of PRKDC compared to the S-I subtype (Wilcoxon test) (Fig. 4c). We then performed comparative analysis to illustrate the potential mechanism associated with the diverse molecular features of S-II and S-III. In addition to having elevated frequencies of *PRKDC* amplification, S-III also had increased frequencies of *CDK4* amplification (18% in S-I, 12% in S-II, and 37% in S-III) (Fig. 4d, e). Moreover, S-II had higher frequencies of *ROCK2* amplification (14% in S-I, 59% in S-II, and 31% in S-III), and *CDK4* amplification and *ROCK2* amplification were mutually exclusive (Fig. 4d, e). S-III also presented significantly higher multigene proliferation scores (MGPSs)^49^, indicating enhanced enrichment of cell proliferation at the proteomic level (Fig. 4f). Combined with proteomic data, we observed a *cis* effect of *CDK4* amplification on the upregulation of cognate protein expression (Fig. 4g). In addition, the kinase activity of CDK4 was also significantly greater in the S-III subtype and was positively correlated with the MGPS, suggesting that CDK4 might promote tumor cell proliferation in the S-II subtype through phosphorylation (Fig. 4g, h). To test the clinical relevance of targeting CDK4 for treating melanoma, we collected PDCs from patients who belong to S-II and S-III and treated them with a CDK4 inhibitor. The effects of the CDK4 inhibitor on cell viability were measured. PDCs from patients with S-III disease were more sensitive to the CDK4 inhibitor (Palbociclib) with significantly lower IC_50_ values (median IC_50_: 7.88 μM in PDC_S-II vs 57.07 μM in PDC_S-III) (Fig. 4i).

Intriguingly, compared to patients with only *CDK4* amplification, patients with both *CDK4* and *PRKDC* amplification had significantly greater CDK4 kinase activity and MGPS (Fig. 4j). Along with this finding, the MGPS was dominantly elevated in patients harboring both *CDK4* and *PRKDC* amplifications (Fig. 4k). We then hypothesized that PRKDC could phosphorylate CDK4 and enhance its kinase activity. Therefore, we surveyed the phospho-substrates of PRKDC and observed that the phosphorylation of CDK4 at T172 was positively correlated with PRKDC kinase activity, CDK4 kinase activity and MGPS (Fig. 4l). The elevated phosphorylation of CDK4/T172 in S-III was further confirmed by IHC staining (Fig. 4m).

To confirm the PRKDC-CDK4 cascade in promoting tumor cell proliferation, we collected PDCs from patients for further analysis (PDC_*PRKDC*^*Amp*^&*CDK4*^*Amp*^: patients belonging to S-III and harboring both *PRKDC* amplification and *CDK4* amplification; PDC_WT: patients without *PRKDC* amplification and *CDK4* amplification). We performed comparative analysis between the proteomes of PDC_*PRKDC*^*Amp*^&*CDK4*^*Amp*^ and PRDC_WT. As a result, the levels of proteins enriched in cell proliferation pathways, such as CDK4, CDK6 and MCM6, were significantly higher in PDC_*PRKDC*^*Amp*^&*CDK4*^*Amp*^ (Fig. 4n, o). We also evaluated cell proliferation rates under different treatment conditions (PDC_*PRKDC*^*Amp*^&*CDK4*^*Amp*^ treated with both PRKDC inhibitor (Nedisertib) and CDK4 inhibitor (Palbociclib), with single CDK4 inhibitor, with single PRKDC inhibitor, or without inhibitor; PDC_WT treated with both PRKDC and CDK4 inhibitors, with single CDK4 inhibitor, with single PRKDC inhibitor, or without inhibitor). The combined use of PRKDC and CDK4 inhibitors most significantly decreased the proliferation of PDC_*PRKDC*^*Amp*^&*CDK4*^*Amp*^ cells, demonstrating that PRKDC could enhance the ability of CDK4 to promote tumor cell proliferation in the S-III subtype (Fig. 4p). Together, our data revealed that the cell proliferation feature of S-III was driven by *CDK4* amplification and could be further enhanced by PRKDC inhibitor. The combined use of both CDK4 and PRKDC inhibitors could clinically benefit patients in S-III (Fig. 4q).

### ROCK2 amplification promotes the metastasis of primary melanomas

**Fig. 5:**
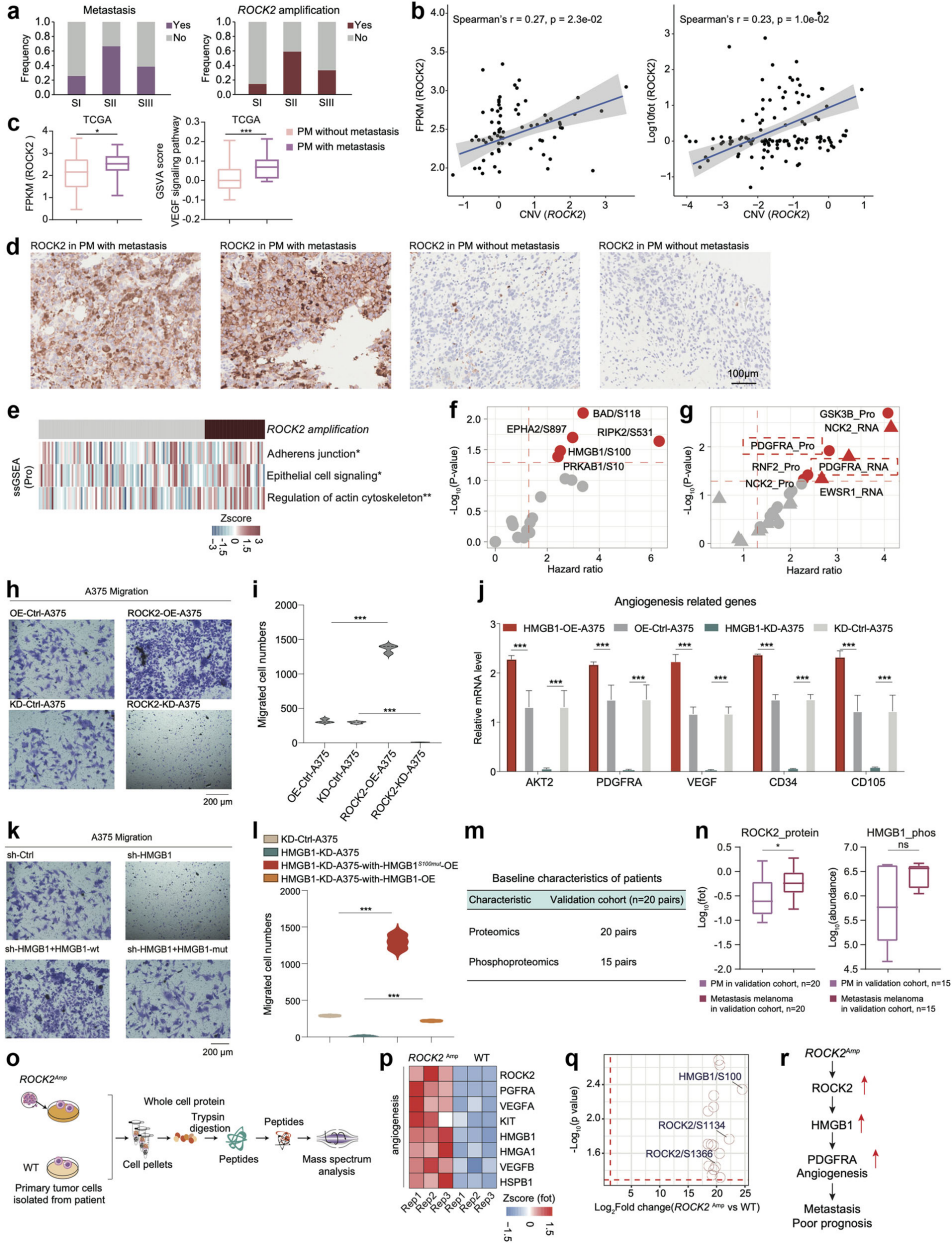
*ROCK2* amplification promoting the metastasis in melanomas. **a** The histogram showed the frequency of metastasis (*n* = 79) and *ROCK2* amplification (*n* = 124). **b** Spearman-rank correlation of the *ROCK2*’s copy number and *ROCK2*’s mRNA expression in melanomas (*n* = 73) (left); Spearman-rank correlation of the *ROCK2*’s copy number and *ROCK2*’s protein expression in melanomas (*n* = 124) (right). **c** The boxplot showed the mRNA expression of ROCK2 and the GSVA score of VEGF signaling pathway in primary melanomas with or without metastasis in TCGA cohort (*n* = 267). **d** Immunohistochemistry of ROCK2 in primary melanomas with or without metastasis, scale bar = 100 μm. **e** The heatmap depicted the pathways significantly elevated in samples harboring *ROCK2* amplificon (*p < 0.05, **p < 0.01, ***p < 0.001, Wilcoxon rank test). **f** The volcano plot showed the abundance of the phosphosites predictive of OS in melanomas. **g** The volcano plot showed the expression of HMGB1’s TGs predictive of OS in melanomas. **h** The effects of ROCK2 on the migration of A375 cells were confirmed by transwell. **i** The violin plots (right panel) indicated counts of migrated A375 cells under different treatments. **j** The boxplot indicated the expression level angiogenesis-related genes across OE-Control-A375, OE-HMGB1-A375, KD-Control-A375, and HMGB1-KD-A375. **k** The effects of HMGB1 on the migration of A375 cells were confirmed by transwell. **l** The violin plots (right panel) indicated counts of migrated A375 cells under different treatments. **m** The table showed the baseline characteristics of patients in the validation cohort1. **n** The boxplot showed the protein expression of ROCK2 and the phosphorylate (*n* = 20) ion abundance of HMGB1 in paired primary melanomas and paired metastasis melanomas in validation corhot1 (*p < 0.05, ****p < 0.0001, Wilcoxon rank test). **o** The workflow showed the sample collection for mass spectrum analysis. **p** Heatmap illustrated the protein expression of ROCK2, VEGFRA, HMGB1, et al. participating in angiogenesis were upregulated in the PDCs from melanoma patients harboring *ROCK2* amplicons. **q** The volcano plot showed the significantly upregulated phosphorylation in the melanoma patients harboring *ROCK2* amplicons. **r** The systematic diagram summarized cascading regulatory role of ROCK2 on angiogenesis, and promoting melanoma metastasis through HMGB1.

Notably, although both the S-II subgroup and the S-III subgroup were associated with poor prognosis, the S-II subgroup contained higher proportion of metastatic patients (Fig. 5a). To further illustrate the possible cellular processes associated with the metastatic features of the S-II subgroup, we first compared the genomic alterations among the three proteomic subgroups and identified *ROCK2* as the only CAG that showed a significantly greater amplification frequency, mRNA expression and protein expression in the S-II subgroup (Spearman’s *r* (CNV vs RNA) > 0.3, *p* < 0.05; Spearman’s *r* (CNV vs protein) > 0.3, *p* < 0.05; Wilcoxon rank test (RNA/protein: S-I vs. S-II/S-I vs. S-III), *p* < 0.05) (Fig. 5b). Functionally, ROCK2 is a kinase participating in angiogenesis^50^ and the epithelial cell signaling pathway^51^ and is associated with tumor cell metastasis^52^. In concordant with our findings, the mRNA expression of ROCK2 and the GSVA score were increased in primary melanomas with metastasis in TCGA cohort^53^ (Fig. 5c). The elevated expression of ROCK2 in primary melanomas with metastasis was further confirmed by IHC staining, using anti-ROCK2 antibody (Materials and methods) (Fig. 5d). In line with these studies, we found pathways, such as regulation of cytoskeleton, epithelial cell signaling pathway, and adherens junction, were significantly elevated in patients harboring *ROCK2* amplicon (Wilcoxon test, *p* < 0.05) (Fig. 5e). Moreover, combined with the phosphoproteomic data, we observed that the phosphosites BAD/S118, RIPK2/S531, EPHA2/S897, HMGB1/S100, PRKAB1/S10, etc., enriched in tumor angiogenesis were significantly positively associated with ROCK2 (Spearman’s *r* > 0.2, *p* < 0.05), confirming the regulatory role of ROCK2 in angiogenesis (Supplementary Fig. S7a). We then performed survival analysis and found that the phosphorylation of HMGB1 at S118 was among the top-ranked ROCK2-correlated phosphosites negatively associated with patient prognosis (hazard ratio > 1, *p* < 0.05) (Fig. 5f). HMGB1 as a TF, has been reported to promote angiogenesis, and VEGF signaling pathway^54,55^. To illustrate the downstream processes driven by HMGB1, we surveyed the expression of HMGB1’s TGs at both the mRNA and protein levels^56^ and found that TGs that were significantly linked to the abundance of HMGB1/S118 were enriched in angiogenesis (Supplementary Fig. S7b). Importantly, PDGFRA, known as the core regulator of angiogenesis, was the only TG that showed a negative correlation with OS at both the mRNA and protein levels, suggesting that HMGB1 might drive patients’ poor prognosis by promoting tumor angiogenesis (Fig. 5g).

To validate the cascade from ROCK2 to HMGB1, we constructed a stable ROCK2-overexpressing A375 cell line (ROCK2-OE-A375) using the pCDH-ROCK2-copGFP vector and knocked down *ROCK2* (ROCK2-KD-A375) utilizing pLKO.1-CMV-shROCK2-copGFP. RT‒PCR analysis was utilized to verify the expression of ROCK2 in ROCK2-OE-A375 and ROCK2-KD-A375 cells. The results confirmed the significantly elevated expression of ROCK2 in ROCK2-OE-A375 cells and the significantly decreased expression of ROCK2 in ROCK2-KD-A375 cells (Supplementary Fig. S7c). We then evaluated the cell migration ability using the Transwell assay. Compared with control cells, the ROCK2-OE-A375 cell line exhibited increased cell migration, whereas the ROCK2-KD-A375 cell line exhibited decreased cell migration (Fig. 5h, i). Based on our integrative analysis, we hypothesized that the kinase ROCK2 might increase cell migration by phosphorylating the TF HMGB1, which could then activate angiogenesis through transcriptional regulation. We then evaluated the phosphorylation of HMGB1 at Ser100 in A375 cell lines with different ROCK2 expression patterns (OE-Control-A375, OE-ROCK2-A375, KD-Control-A375, and ROCK2-KD-A375). The phosphorylation of HMGB1 was significantly elevated in OE-ROCK2-A375 cells than in OE-Control-A375 cells and significantly decreased in ROCK2-KD-A375 cells than in sh-Control-A375 cells (Supplementary Fig. S7d).

Based on these findings, we investigated the impact of HMGB1 on the downstream angiogenesis process. We constructed a stable HMGB1-overexpressing A375 cell line (HMGB1-OE-A375) using the pCDH-HMGB1-copGFP vector and knocked down HMGB1 (HMGB1-KD-A375) utilizing pLKO.1-CMV-HMGB1-copGFP. We then conducted RT‒PCR to evaluate the expression of angiogenesis-related proteins across cells (HMGB1-OE-A375, HMGB1-KD-A375, OE-Control-A375, and KD-Control-A375). The results revealed that the expression of genes involved in angiogenesis was greater in HMGB1-OE-A375 than in OE-Control-A375. However, compared to those in KD-Control-A375 cells, the expression of angiogenesis process-related genes decreased in HMGB1-KD-A375 cells (Fig. 5j). These results confirmed the regulatory role of HMGB1 in promoting angiogenesis. To further validate the role of HMGB1, especially phosphorylated HMGB1, in promoting tumor cell migration, we constructed an HMGB1-OE vector and an HMGB1-S100-mutation-OE vector and transfected them into the HMGB1-KD-A375 stable cell line. We then evaluated the cell migration ability by Transwell assays. Compared to KD-Control-A375, HMGB1-KD-A375 significantly decreased tumor cell migration. The decrease in cell migration was only reversed by transfection with the HMGB1-OE vector, while it remained unchanged by transfection with the HMGB1-S100-mutation-OE vector (Fig. 5k, l). These results demonstrated that the phosphorylation of HMGB1 at S100 plays a crucial role in promoting tumor cell migration.

To further confirm that the ROCK2 (kinase)-HMGB1 TF-PDGFRA TG cascade promotes tumor metastasis, we constructed an independent validation cohort including 20 melanoma patients and collected matched primary and metastatic melanoma tumor samples for proteomic and phosphoproteomic analysis (Fig. 5m). We compared the expression of ROCK2 and the phosphorylation of HMGB1 between matched primary and metastatic melanoma tumor samples. As a result, the protein expression of ROCK2 and the phosphorylation of HMGB1 were elevated in metastatic samples (Fig. 5n).

Moreover, to validate the potential causal link of ROCK2 in promoting tumor metastasis through the phosphorylation of HMGB1, we also collected PDCs from patients (PDC_*ROCK2*^*Amp*^: patients harboring *ROCK2* amplification, PDC_WT: patients without *ROCK2* amplification) and further conducted proteomic and phosphoproteomic analysis (Fig. 5o). As a result, the comparative analysis between PDC_*ROCK2*^*Amp*^ and PDC_WT revealed that the levels of proteins involved in angiogenesis, including ROCK2, PDGFRA, VEGFA, and HMGB1, were significantly higher in PDC_*ROCK2*^*Amp*^. Additionally, at the phosphoproteome level, the phosphorylation of HMGB1 at S100 was the most significantly elevated phospho-substrate of ROCK2 (Fig. 5p, q). Collectively, our data illustrated that the elevated amplification of *ROCK2* in the S-II subgroup could be responsible for the elevated angiogenesis and might serve as a possible predictive marker for melanoma metastasis (Fig. 5r). In general, proteomic-centered multi-omics analysis helped to elucidate the distinctive molecular mechanism that led to poor prognosis in S-II and S-III patients. Specifically, *PRKDC* amplification coupled with *CDK4* amplification increased cell proliferation in S-II, whereas *ROCK2* amplification elevated the increased angiogenesis and promoted melanoma metastasis in S-III.

### Immune subgroups with distinct biological and clinical features

**Fig. 6:**
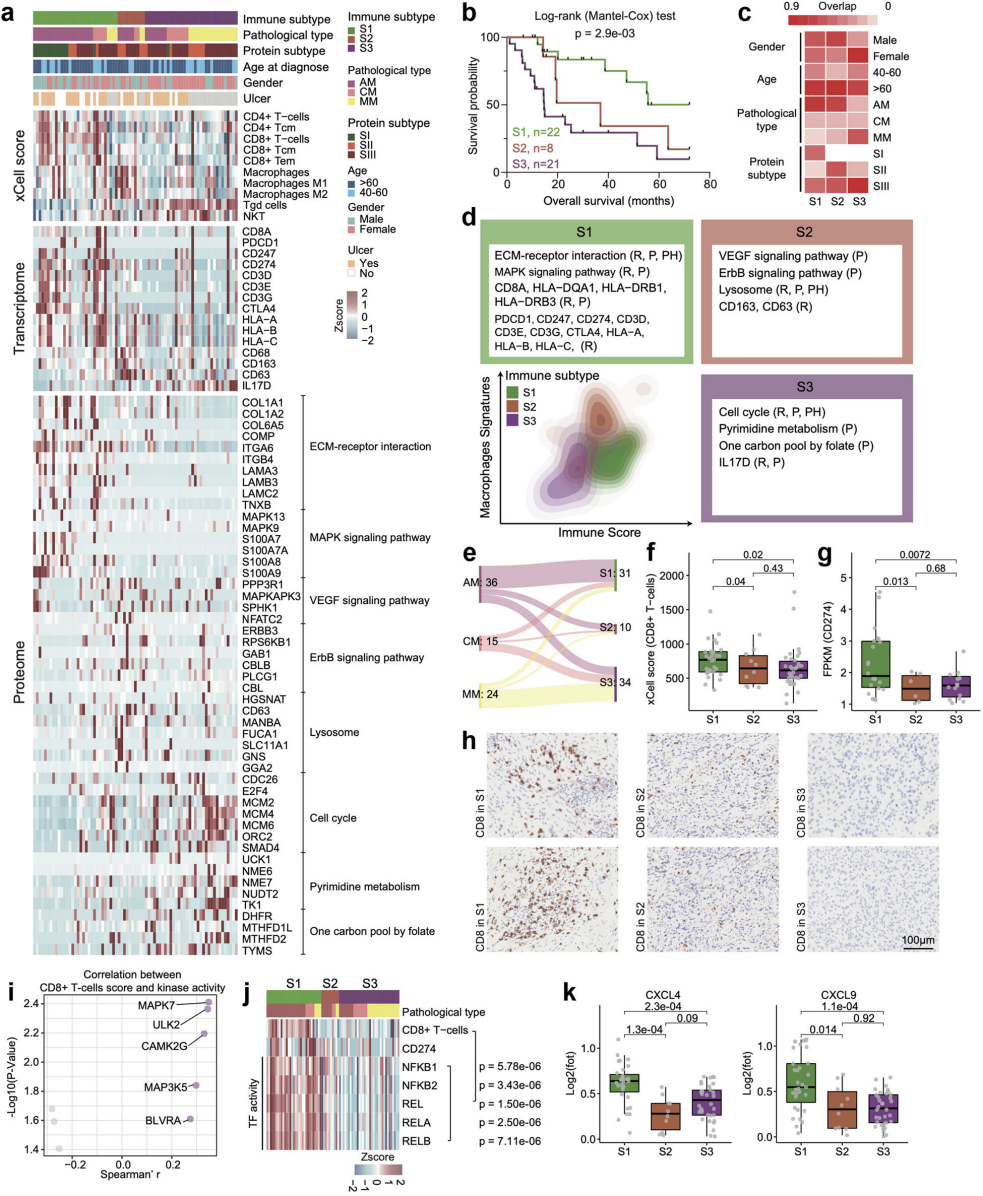
Immune landscape in melanomas. **a** Heatmap illustrated cell type compositions and activities of selected individual mRNAs/proteins and pathways across three immune clusters in 75 melanoma patients. The heatmap in the first section illustrated the immune signatures based on analysis using xCell. The heatmap in the second section illustrated the RNA and protein abundance of key immune-related markers. The remaining section indicated the expression patterns of proteins which showed significantly upregulated in the three immune subgroups, respectively. **b** The association of three immune groups with clinical outcomes in melanoma patients (S1: *n* = 22; S2: *n* = 8; S3: *n* = 21) (*p* value based on the log-rank test). **c** Heatmap showed the comparison between immune clusters (columns) with sex, age, proteomic subtypes, and different histological types. **d** Contour plot of two-dimensional density based on macrophage (*y*-axis) and immune scores (*x*-axis) for different immune groups. For each immune group, key upregulated pathways and molecules were reported based on RNA-seq (R), global proteomics (P), and phosphoproteomics data (Ph) in the annotation boxes. **e** Sankey plot showed the comparison between immune clusters (columns) with different histological types. **f** The boxplot showed the xCell score of CD8^+^ T cells in the three immune clusters (*n* = 75) (Wilcoxon rank test). **g** The boxplot showed the mRNA expression of CD274 in the three immune clusters (*n* = 75) (Wilcoxon rank test). **h** IHC of CD8 in the three immune clusters, scale bar = 100 μm. **i** The volcano plot showed the kinases whose kinase activity was significantly correlated with the xCell score of CD8^+^ T cells. **j** The heatmap showed the TF activity of NFκB family were positively correlated with the xCell score of CD8^+^ T cells. **k** The boxplot showed the protein expression of CXCL4 and CXCL9 in the three immune clusters (*n* = 75).

Although immunotherapy has been used in the field of melanoma treatment, its efficacy varies among patients. To better understand the features of immune infiltration in melanomas, we performed xCell analysis based on RNA-seq data to infer the relative abundance of different cell types in the tumor microenvironment. Consensus clustering based on inferred cell proportions helped identify the three sets of tumors with distinct immune signatures (S1‒S3) (Fig. 6a and Supplementary Fig. S8a, b and Table S4) (Materials and methods). Survival analysis indicated that the immune subgroups significantly differed in terms of OS (log-rank test, *p* = 0.0029), suggesting that different types of immune cell infiltration can lead to diverse prognostic outcomes (Fig. 6b). In addition, we observed that ~50% of AM was distributed in S1 subtype, while the majority of MM was distributed in S3 subtype, and the proportion of female patients in S3 subtype was significantly higher than the other two subtypes (Fig. 6c). We also looked over the frequency of *PRKDC* amplification and the expression of PRKDC across immune subtypes. As a result, we found that the frequency of *PRKDC* amplification was significantly higher in S2 and S3 than S1 (S1: 56%, S2: 88%, S3: 74%), as well as the protein expression of PRKDC (Supplementary Fig. S8c).

Among the three subgroups, S1 was characterized by CD4^+^ T cells and CD8^+^ T cells (referred to as the T-cell subtype) (Fisher’s exact test, *p* < 0.05), and the mRNAs of CD8A, PDCD1, CD247, CD274, and CD3D were dominantly expressed in the S1 subtype. Pathways such as the ECM−receptor interaction and MAPK signaling pathways were dominantly expressed in the S1 subtype (Fig. 6a, d). Correlation analysis showed the PRKDC expression was not only negatively correlated with MAPK7 expression but also negatively correlated with the xCell score of CD8^+^ T cells and the CD274 expression (Supplementary Fig. S8d). Meanwhile, S2 was featured with macrophage signatures (referred to as the TAM subtype), and mRNAs including CD68, CD163 and CD63 were overrepresented in this subtype. Proteins participating in VEGF signaling pathway, ErbB signaling pathway, and lysosome were overrepresented in this subtype (Fig. 6a, d). Meanwhile, the polarization score of macrophages suggested that more M1 macrophages polarized toward M2 macrophages in S2 subtype (ANOVA test, *p* < 1.0e−04) (Supplementary Fig. S8e). Moreover, S3 was enriched with γδ T cell and NKT cell signatures and the mRNA expression of IL-17 were increased in this subtype (referred to as the IL-17 secretion subtype). Pathways such as cell cycle, pyrimidine metabolism and one carbon pool by folate were dominantly expressed in S3 subtype (Fig. 6a, d). Meanwhile, the cytokines/chemokines such as CCL2, CCL14, CCL15 and CCL22, that associated with γδ T cell recruitment were elevated in S3 subgroup (Supplementary Fig. S8f). IHC staining using CD8, CD163 and IL17 further confirmed enrichment of CD8 in the S1 subtype, macrophage in S2 subtype and γδ T cells in S3 subtype, respectively (Fig. 6h and Supplementary Fig. S8g, h).

Importantly, compared to S2 and S3, the S1 subtype included more AM patients (AMs in S1:22, AMs in S2:6, and AMs in S3:8), implying that a considerable number of AM patients might exhibit elevated immune cell infiltration (Fig. 6e). Additionally, since we observed elevated PD-L1 expression and high enrichment of CD8^+^ T cells in the S1 subtype (Wilcoxon test, *p* < 0.05) (Fig. 6f, g), we hypothesized that patients in the S1 subtype might have higher sensitivity to immunotherapy than patients in the other subtypes. To elucidate the possible mechanism underlying this phenomenon, we compared the molecular features of the three immune subtypes and found that the MAPK signaling pathway and NFκB signaling pathway were positively correlated with the CD8^+^ T cell signature score. Along with this observation, by comparing the kinase activity among the three immune subgroups, we found that the kinase activity of MAPK7 (ERK5) was the top ranked kinase associated with the CD8^+^ T cell signature score (Spearman’s *r* > 0.2, *p* < 0.05) (Fig. 6i). Previous studies by us and other groups have indicated that MAPK7 can activate NFκB1/2 via phosphorylation. Since NFκB1/2 are TFs, they can further increase the expression of their TGs, such as PD-L1, and other cytokines/chemokines, such as CXCL4, CXCL5, and CXCL9^57^. We then evaluated the TF activity of NFκB2 and found that the TF activity of NFκB1/2 was also significantly positively correlated with the enrichment scores of CD8^+^ T cells (Fig. 6j). Along with this finding, cytokines/chemokines that participate in T-cell recruitment, such as CXCL4 and CXCL9, were elevated in the S1 subgroup (Fig. 6k). In summary, these results suggested that the elevated kinase activity of MAPK7 might enhance the TF activity of NFκB2 and in turn increase cytokine expression and recruitment of CD8^+^ T cells. Thus, patients in S1 might benefit from immunotherapy.

### The refined subtype including the information of both the immune and proteomic subtype and correlated with OS

In the previous analysis, we found there seemed to be a strong correlation between immune subtype and protein subtype (Fig. 6c), and both of them had a strong association with OS (Figs. 4b and 6b). We further employed hierarchical clustering based on proteomic and immune subtyping signatures among 3 histological subtypes of melanomas to integrate the proteomic and immune subtypes into a refined subtype. R (version 4.2.0) and the R package “factoextra” (version 1.0.7) were utilized for data process. As a result, we identified five subgroups (HC1, HC2, HC3, HC4, and HC5). Remarkably, survival analysis revealed that hierarchical clusters significantly differed in terms of OS (log-rank test, *p* = 0.01) (Supplementary Fig. S9a). The evaluation of the clinical features of proteomic subtypes revealed that HC1 and HC4 patients had a significantly longer OS, and the HC3 subgroup exhibited a higher probability of mucosal melanoma than did the other subgroups (0% in HC1, 40% in HC2, 75% in HC3, 31% in HC4, and 40% in HC5) (Supplementary Fig. S9a, b). We further compared the frequencies of *PRKDC* amplification, *CDK4* amplification, *ROCK2* amplification, protein expression, and xCell immune signatures among the five subtypes. HC1 was characterized by lower frequencies of *PRKDC* amplification, *CDK4* amplification, and *ROCK2* amplification; lower protein expression of PRKDC, CDK4, and ROCK2; and higher CD8^+^ T-cell signature than the other subtypes, similar to the features of the SI proteomic subtype (ECM subtype) and S1 immune subtype (T-cell subtype) in our previous analysis (Supplementary Fig. S9b–d). HC2 patients with high frequencies of *PRKDC* amplification, *CDK4* amplification, and *ROCK2* amplification; high protein expression of PRKDC, CDK4, and ROCK2; and high macrophage signatures had a worse prognosis, similar to patients with the characteristics of the SII proteomic subtype (angiogenesis subtype) and S2 immune subtype (TAM subtype) (Supplementary Fig. S9b–d). HC3 patients also exhibited high frequencies of *PRKDC* amplification and *ROCK2* amplification, high protein expression of PRKDC and ROCK2, and a poor prognosis; moreover, the Tgd cell signature was enriched in HC3 patients and concordant with the signatures of the SII proteomic subtype (angiogenesis subtype) and S3 immune subtype (Tgd cell subtype) (Supplementary Fig. S9b–d). We observed that both HC4 and HC5 had high frequencies of *PRKDC* amplification, *CDK4* amplification, and high protein expression of PRKDC and CDK4, similar to the SIII proteomic subtype (cell proliferation subtype) (Supplementary Fig. S9b–d), while the prognosis and immune features of HC4 and HC5 were quite different. HC4 was enriched with the CD8^+^ T-cell signature and had a better OS, similar to the S1 immune subtype (T-cell subtype). Moreover, HC5 was enriched in the Tgd cell signature and had a worse OS, similar to the S3 immune subtype (Tgd cell subtype). Interestingly, these refined subtypes implied that the same proteomic subtypes in our study possessed different immune groups (HC1: SI-S1; HC2: SII-S2; HC3: SII-S3; HC4: SIII-S1; HC5: SIII-S3).

We further explored the mechanism associated with differences in immune features between HC4 and HC5 patients. By comparing the immune features between the two subgroups, we found that the xCell score of CD8^+^ T cells and the expression of the immune checkpoint molecule CD274 in HC4 patients were obviously greater than those in HC5 patients (Supplementary Fig. S9e). It has been reported that the expression of CD274, commonly referred to as PD-L1, is significantly correlated with the infiltration of CD8^+^ T cells and could help predict survival and therapeutic responses^58^. Moreover, the xCell score of Tgd cells and the expression of the key molecule IL17D in HC5 cells were obviously greater than those in HC4 cells (Supplementary Fig. S9e). A protumor role for IL-17-producing Tgd cells was reported in human cancer, and the extent of IL-17-producing Tgd cell infiltration positively correlated with the clinical stage of the disease^59^. We then conducted comparative analysis and Gene Ontology (GO) enrichment analysis between HC4 and HC5 and found that the biological features of HC4 were related to antigen processing and presentation, cell adhesion molecules, and the T-cell receptor signaling pathway (Supplementary Fig. S9f). Proteins participating in metabolic pathways, the mTOR signaling pathway, and pyrimidine metabolism were enriched in HC5 (Supplementary Fig. S9f). This result showed that although HC4 (SIII-S1) and HC5 (SIII-S3) exhibited genomic and proteomic similarities, the differences between immune features might contribute to differences in clinical outcomes.

Comparative analysis of the phosphoproteome between HC4 and HC5 revealed that kinases, such as MAPK7, MAP2K2, and MAP3K1, which are enriched in the MAPK signaling pathway, were elevated in HC4; kinases, such as AKT3, PIK3C2A, and PRKDC, which are involved in the cell cycle, were elevated in HC5 (Supplementary Fig. S9g). Intriguingly, there were no differences in the frequency of *PRKDC* amplification or the protein expression of PRKDC between HC4 and HC5, while the kinase activity of PRKDC was elevated in HC5. Furthermore, survival analysis revealed that increased kinase activity of AKT3 was associated with poor OS (Supplementary Fig. S9h). We investigated the relationship between MAPK7 and CD8^+^ T cells in our previous manuscript; therefore, we further explored the role of AKT3 in the immune microenvironment in the revision. Correlation analysis revealed that the kinase activity of AKT3 was positively correlated with the xCell score of Tgd cells and the ssGSEA score of the cell cycle (Supplementary Fig. S9i). AKT contributes not only to the regulation of Tgd cell development but also to the functional regulation of these cells^60^. Donghai et al.’s study indicated that proteins participating in the cell cycle, such as CDC5A, CDCA8, TK1, and TYMS, which were elevated in HC5 (Supplementary Fig. S9j), could be correlated with the contribution of Tgd cells to immune checkpoint resistance^61^.

To summarize, we performed clustering analysis of the proteome and immune microenvironment. Proteomic clustering revealed key kinases and biological pathways involved in distinguishing patients with melanoma. Through immune clustering, we revealed that the heterogeneity of the TME in patients with melanoma and immune features were correlated with clinical outcomes. Integration of proteomic and immune subtypes could provide refined melanoma subgroups and reveal their specific characteristics.

### Identification of protein markers related to the response to immunotherapy in the melanomas

**Fig. 7:**
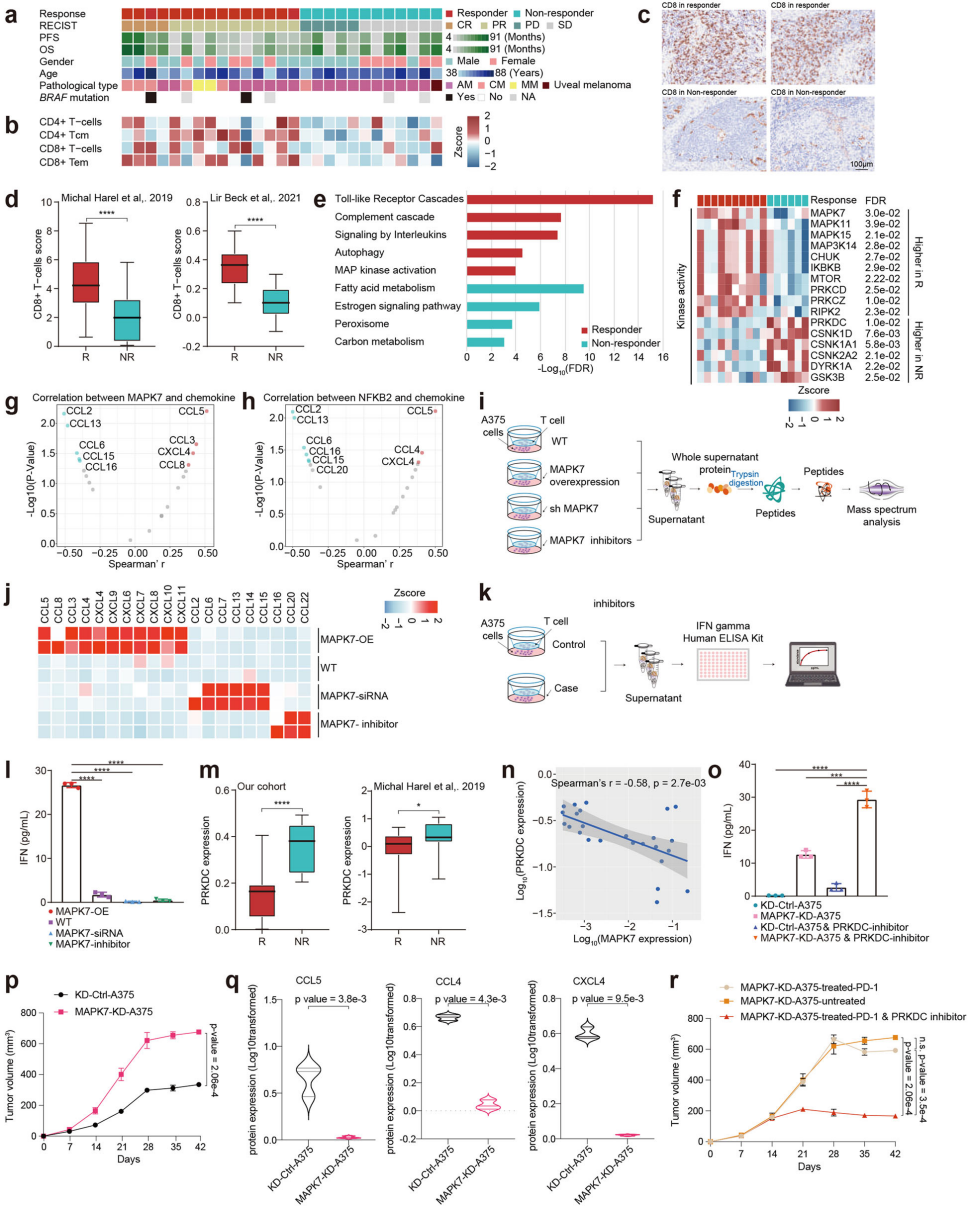
Identification of protein markers of response to immunotherapy in the melanomas. **a** The heatmap showed clinical information of the anti-PD-1 cohort (*n* = 27). **b** The heatmap illustrated the immune signatures based on analysis using xCell. **c** Immunohistochemistry of CD8 in the responders and non-responders, scale bar = 100 μm. **d** The boxplot showed the xCell score of CD8^+^ T-cells in the responders and non-responders in Harel et al.’ cohort^62^ (*n* = 116) and Beck et al.’ cohort^63^ (*n* = 185) (Wilcoxon rank test). **e** The histogram showed the biological pathways upregulated in responders and non-responders. **f** The heatmap showed the kinases whose kinase activity upregulated in responders and non-responders (*n* = 16). **g** The volcano plot showed the chemokines whose protein expression was significantly correlated with the kinase activity of MAPK7. **h** The volcano plot showed the chemokines whose protein expression was significantly correlated with the protein expression of NFKB2. **i** The workflow showed the sample collection for MS analysis. **j** Heatmap illustrated the protein expression of chemokines in the A375 cells under different treatment conditions. **k** The workflow showed the sample collection for enzyme-linked immunospot (ELISPOT) assay. **l** The histogram showed the concentration of IFN in the supernatant of A375 cells cultures with T cells under different treatment conditions, including MAPK7-overexpression, WT, MAPK7-siRNA, and MAPK7-inhibitor. The data represent the mean values ± SD (*n* = 3). **m** The boxplot showed the protein expression of PRKDC in the responders and non-responders in our cohort (*n* = 27) and Harel et al.’ cohort^62^ (*n* = 116) (Wilcoxon rank test). **n** Spearman-rank correlation of the MAPK7’s protein expression and PRKDC’s protein expression in melanomas. **o** The histogram showed the concentration of IFN in the supernatant of A375 cells cultures with T cells under different treatment conditions (KD-Ctrl-A375 cells, MAPK7-KD-A375 cells, KD-Ctrl-A375 cells treated with PRKDC inhibitor, MAPK7-KD-A375 cells treated with PRKDC inhibitor (NU7441)). The data represented the mean values ± SD (*n* = 3). **p** Tumor growth curves (*n* = 3 replicates per group) (mean ± SEM). **q** Boxplots indicated the protein expression of CCL5, CCL4, and CXCL4 between KD-MAPK7-A375 tumors and KD-Ctrl-A375 tumors. **r** Tumor growth curves (*n* = 3 replicates per group) (mean ± SEM).

Based on the findings above, to further illustrate the potential association between MAPK7 and efficiency of immunotherapy in melanomas, we constructed an independent validation cohort, containing 27 stage IV melanoma patients with anti-PD1 treatment (18 AMs, 6 CMs, 2 MMs). Patients were grouped based on their response to the treatment, with 15 responders (including partial and complete response; *n* = 15) and 12 non-responders (including stable disease and progressive disease; *n* = 12) (Fig. 7a). Tumor samples before treatment were collected, and we performed both proteomic and phosphoproteomic analysis.

We first applied cell deconvolute analysis and compared immune cell enrichment between responders and non-responders. As a result, CD4^+^ T-cells and CD8^+^ T-cells signatures were enriched in responders (Fig. 7b). IHC staining utilizing anti-CD8 antibody also confirmed the enrichment of CD8 in responders (Fig. 7c). In concordant with our finding, the enrichment of CD8^+^ T-cells in responders were also validated in two previous studies by Harel^62^ and Beck^63^ (Fig. 7d). Furthermore, we performed comparative proteomic analysis between responders and non-responders, as a result, in concordant with the molecular features of immune subgroup of S1, the pathways that enriched by the proteins that elevated in responders were mainly MAPK signaling pathway and immune related pathway, whereas non-responders were dominated by metabolic proteins (Fig. 7e). We also compared the kinase activity between responders and non-responders. As a result, MAP kinases, MAPK7 (ERK5), MAPK15 (ERK7), MAPK11, and MAP3K14, were highly expressed in responders, whereas DNA repair-related kinases, PRKDC (DNAPK), CSNK1A1 (CK1A), CSNK2A2 (CK2A2), and DYRK1A were highly expressed in non-responders (Fig. 7f).

Importantly, to verify whether activation of MAPK7-NFκB2 cascading contributed to the increased expression of cytokines that participating in recruiting T cells, we performed correlation analysis, and observed cytokines/chemokines such as CCL5, CXCL4 and CCL4 were positively correlated with the kinase activity of MAPK7 and protein expression of NFκB2 (Fig. 7g, h). In concordantly, we constructed MAPK7-overexpressing, MAPK7 knock-down A375 cells, treated A375 cells with MAPK7 inhibitor (XMD8-92) and utilized wild type A375 cells as control. We co-cultivated those cells with CD8 T cells and collected supernatant for proteomic analysis (Fig. 7i). As a result, cytokines participating in recruiting T cells, such as CCL4, CCL8, and CXCL4, were significantly upregulated in cultivate supernatant of MAPK7-overexpressing A375 cells (Fig. 7j). On the contrary, the cytokines/chemokines that participated in recruiting IL-17 T cells including CCL2/6/7/13/14 etc. were significantly upregulated in cultivate supernatant of MAPK7 knock-down A375 cells (Fig. 7j). Meanwhile, the IFNγ ELISA assay showed that the CD8^+^ T cells co-cultivated with MAPK7-overexpressing A375 cells were activated most significantly (Fig. 7k, l). These results demonstrated the impact of MAPK7 in recruiting and activating CD8^+^ T cells and facilitated the immunotherapy.

To identify potential druggable targets that could promote the efficacy of anti-PD-1 immunotherapy, we focused on kinases whose expression was elevated in the non-responder group. PRKDC was the most significantly elevated kinase in the non-responders. This phenomenon was further confirmed in Michal Harel’s cohort^62^ (Fig. 7m). Accordingly, the protein expression of PRKDC was negatively correlated with the protein expression of MAPK7 (Fig. 7n). To evaluate the potential of targeting PRKDC to enhance the efficacy of immunotherapy, we cocultivated T cells with A375 cells under various treatment conditions (MAPK7-knockdown A375 cells, A375 cells treated with a PRKDC inhibitor, MAPK7-knockdown A375 cells treated with a PRKDC inhibitor and MAPK7-overexpressing A375 cells treated with a PRKDC inhibitor). We then performed an IFNγ ELISA and found that MAPK7-overexpressing A375 cells and the PRKDC inhibitor most significantly activated CD8^+^ T cells (Fig. 7o). These results emphasized that decreased expression of PRKDC could significantly activate CD8^+^ T cells.

Based on these findings, we further constructed a xenograft melanoma mouse model using the MAPK7-KD-A375 stable cell line and used the KD-Control-A375 cell line as a control. The xenograft tumors were continuously measured for three weeks and then treated with a PD-1 inhibitor or a combination of PRKDC (NU7441) and a PD-1 inhibitor or left untreated. During the three weeks of treatment, the tumor sizes were continuously measured. Compared to mice transplanted with KD-Control-A375 cells, mice transplanted with MAPK7-KD-A375 cells were not sensitive to PD-1 treatment (Fig. 7p), indicating that decreased MAPK7 expression significantly reduced patients’ response to PD-1 treatment. Consistent with this finding, we observed that compared to those in mice transplanted with KD-Control-A375 cells, the expression of proteins associated with T-cell recruitment, such as CCL5 and CCL3, was significantly lower in mice transplanted with MAPK7-KD-A375 cells (Fig. 7q). Moreover, for mice transplanted with MAPK7-KD-A375 cells, compared to PD-1 treatment alone, the combination of PRKDC and a PD-1 inhibitor significantly reduced tumor growth (Fig. 7r), confirming the potential of using a PRKDC inhibitor combined with an anti-PD-1 antibody for melanoma treatment in the future.

In summary, our study revealed the proteogenomic landscape of melanoma. We identified SBS7a as the major mutational signature of melanomas associated with elevated DNA damage repair. Further analysis revealed that amplification of the DNA damage repair-related kinase *PRKDC* was a potential prognostic molecule for melanoma. Integrative analysis combined with functional experiments illustrated that *PRKDC* amplification might lead to tumor proliferation by activating the DNA repair process and folate metabolism pathway. Proteome-based stratification of PMs (CMs, AMs, and MMs) revealed three prognosis-related subtypes, which could complement histological subtypes and provide an essential framework for the utilization of specific targeted therapies for particular melanoma subtypes. Immune clustering identified three immune subtypes with distinctive immune cell types. Further analysis revealed that increased MAPK7-NFκB signaling accounts for the increase in T-cell infiltration in patients with melanoma. Additionally, using an independent anti-PD-1 treatment cohort, we confirmed the importance of the MAPK7-NFκB signaling pathway in determining the efficacy of immunotherapy and suggested that the combination of anti-PD-1 and PRKDC inhibitors might improve patient outcomes (Supplementary Fig. S10a).

## Discussion

Epidemiological studies have shown that CMs mainly occur in white populations with fair skin (> 95%), whereas pigmented populations from Asia mainly develop AMs (~50%) and MMs (20%–30%)^6,8^. Therefore, our cohort was composed of 28 CMs (20.4%), 81 AMs (59.2%), and 28 MMs (20.4%), while most previous studies focused on cutaneous melanomas, and a few studies reported small-scale cohorts of patients with acral melanomas or mucosal melanomas. The similarity of genomic alterations in our cohort and Western melanoma cohorts indicated that the difference between the Chinese melanoma cohort and Western melanoma cohort was the incidence of different histological types rather than the difference in molecular expression in one histological type between different populations. In our study, the integration of multi-omics and clinical information on melanomas could be beneficial for understanding how genetic variation affects molecules and identifying the potential underlying mechanism involved. Moreover, our proteogenomic study included three main pathological melanoma types (AMs, MMs, and CMs), and a considerable number of AMs might be conducive to enhance the awareness of melanomas.

Recent genome sequencing studies have revealed landscapes of somatic mutations in various normal tissues, enhancing our knowledge of mutagenesis in somatic cells^64–66^. Our study showed that *BRAF* mutations can occur widely in comparatively benign tissues^67^, emphasizing the complexity of melanoma tumorigenesis and malignant melanoma. Moreover, our analyses identified novel genomic variations that augmented our understanding of melanoma risk and provided new insights into melanoma etiology. Although UV damage is known to be a risk factor for melanoma^68,69^, the impact of UV damage on prognosis and downstream biological pathways has not yet been elucidated. In our research, we found that the mutational signature SBS7a (UV damage related) was significantly associated with patients’ clinical outcomes. Further integrative analysis revealed that patients harboring SBS7a had significantly elevated expression of DNA damage repair-related proteins, such as PRKDC, ATR, and POLD4, suggesting that elevated DNA damage repair contributes to poor prognosis in patients with melanoma.

The *PRKDC* gene encodes DNA-dependent protein kinase (DNA-PK), which plays a pivotal role in DNA double-strand break repair^38^. Previous studies have reported mutational alterations and abnormal expression of *PRKDC* in various cancer types^70–72^, including gliomas, colorectal carcinoma (RCC), and nasopharyngeal carcinoma. Specifically, McKean-Cowdin et al.^72^ proposed that mutations in DDR genes were associated with an increased risk of glioblastoma multiforme (GBM), and a variant of *PRKDC* increased the risk of glioma by 44%. In addition, Zheng et al.^73^ reported that in RCC, the overexpression of DNA-PKcs was significantly associated with enhanced tumor cell proliferation. In our study, we found that *PRKDC* amplification was a prognostic marker for melanoma. Taking advantage of our multi-omics studies, we further elucidated the mechanism by which *PRKDC* might promote tumor cell proliferation through its *cis*-effect and collaborate with ATM and ATR.

Moreover, in recent years, the therapeutic role of PRKDC has been reported. Sun et al.^40^ reported a significant correlation between PRKDC expression and the response to chemotherapy in patients with breast cancer. In our research, we investigated possible therapeutic strategies for patients harboring *PRKDC* amplifications and confirmed that enhanced PRKDC expression might inhibit the efficacy of 5-FU treatment. Since the current treatment with chemotherapy has been shown to have a very minor effect on melanoma, our results suggest a potential option to enhance the therapeutic efficacy for treating melanoma in the future.

The folate cycle^74^ within folate metabolism promotes cell growth and proliferation. In this case, one-carbon units were used for purine and thymidine synthesis^43^. In contrast, the methionine cycle within folate metabolism plays a role in inhibiting cancer cell growth and proliferation because one-carbon units are used for DNA or protein methylation, which leads to gene expression silencing and chromosome condensation. The MTR is the unique linker of the two coupled cycles^75^. The one-carbon unit flux determines the balance between the promotion or inhibition of growth. This study is the first to demonstrate the benefits of uncoupling the folate cycle and the methionine cycle on cancer cell growth and proliferation. Uncoupling these two cycles enhanced the oncogenic effects of one-carbon metabolism because most methyl units derived from serine were used in purine and pyrimidine synthesis. Additionally, MTR deficiency results in the blockade of the synthesis of methionine from the remethylation of homocysteine, thereby leading to decreased cellular methionine and SAM concentrations^76^. In certain patients who presented with a loss of MTR, BHMT expression increased to compensate for MTR loss. BHMT catalyzes the remethylation of homocysteine and results in the generation of methionine using betaine as a methyl donor^77^. In our previous study, we also observed decreased MTR activity induced by genetic variants that contributed to the development of prostate cancer and congenital heart disease, which are common diseases related to the dysregulation of folate metabolism^43^. These phenomena indicate that uncoupling the folate and methionine cycles in one-carbon metabolism contributes to the development of a wide array of folate metabolism dysfunction-related diseases.

Although histological diagnosis remains the cornerstone of the classification of tumors into therapeutic categories, it is now well recognized that molecular subgroups within histologically similar tumors can be identified based on transcriptomics and genomics^78,79^. Using proteomic data, we clustered the PM into three subgroups, which exhibited remarkable diversity in molecular signatures and patient survival. Although the amplification frequency and protein expression of *PRKDC* did not differ between subgroup II and subgroup III, as characterized by diverse proteomic features across the three proteomic subgroups, we further indicated that the elevated frequency of *ROCK2* amplification contributed to the dominant angiogenesis feature of subgroup II, which might partly explain the greater proportion of metastatic patients in subgroup II. In subgroup III, *CDK4* amplification and *PRKDC* amplification jointly promoted increased CDK4 protein expression and kinase activity in melanoma patients, accelerating the proliferation rate of melanoma and leading to poor prognosis.

Melanoma is among the most sensitive malignancies to immune modulation. Although multiple trials conducted over decades with vaccines, cytokines and cell therapies have demonstrated meaningful responses in a small subset of patients with melanomas, approximately half of all melanoma patients treated with immune checkpoint inhibitors (ICIs) exhibit resistance or recurrence^80^. Currently, no highly accurate predictive biomarkers exist, and there are limitations. According to our data, by conducting immune-based subtyping of CM, AM, and MM samples via deconvolution of cell composition, we identified three immune subtypes: the T-cell infiltration type, TAM infiltration type, and IL-17 secretion type. Combined with the results of proteomic and phosphoproteomic data analysis of the anti-PD-1 treatment cohort, we found that the upregulation of MAPK7 (ERK5) kinase activity and NFκB transcription activity may lead to more T cells being recruited into the tumor microenvironment, thereby increasing the sensitivity of patients to immunotherapy. Previous studies by us and others have proven the relationship between the MAPK7-NFκB signaling pathway and PD-L1 expression^57,81^. Here, we observed that the activated kinase activity of MAPK7 might lead to the recruitment of CD8^+^ T cells to the tumor microenvironment and might contribute to favorable outcomes in patients receiving anti-PD-1 therapy. We then constructed an independent anti-PD-1 validation cohort and evaluated correlations among MAPK7, elevated CD8^+^ T-cell recruitment and favorable clinical outcomes for patients receiving anti-PD-1 therapy. Moreover, we found that PRKDC might reduce the sensitivity of melanoma patients to immunotherapy by promoting DNA repair and cell proliferation. In clinical cohorts of patients with NSCLC and melanoma, Tan et al. reported that for patients treated with ICIs, those with *PRKDC* mutations were confirmed to have a better prognosis^38,82^. We evaluated the potential therapeutic potential of targeting PRKDC to enhance the efficacy of immunotherapy and confirmed the potential therapeutic options involving the use of PRKDC inhibitors combined with anti-PD-1 therapy for melanoma treatment in the future. These insights might help extend new treatments that could be effective for one type of tumor and for histologically disparate tumors that share the same immunological features.

In summary, our study presented a comprehensive proteomic landscape of melanomas, including acral, cutaneous, and mucosal types. Our data provide a resource to illustrate the functional mechanism of driver genomic alterations that impact survival, treatment and other clinical factors affecting patient outcome and quality of life.

## Materials and methods

### Clinical sample collection

Archival FFPE tissues obtained from 197 participants without prior treatment were selected for the conduction of the present study, including primary CMs (*n* = 28), primary AM (*n* = 81), primary MM (*n* = 28), metastatic melanomas (*n* = 27), and nevi (*n* = 43). Nevi samples were obtained from 43 patients who underwent dermal nevus surgery in 2019. All patients were diagnosed with melanomas from 2006 to 2018 at Zhongshan Hospital and received no prior anticancer treatments regardless of histologic grade or surgical stage. Melanomas were graded and staged using the 8th edition of the American Joint Committee on Cancer (AJCC)^83^. Each tissue specimen endured cold ischemia for average 30 min or less prior to being paraffin-embedded with 3.7% neutral buffered formaldehyde solution fixation for 6–48 h. Many pre-analytical variables have been evaluated and no detrimental effect was found so far on protein abundance and quality due to storage times of FFPE specimens for ten or more years^84,85^. Selection of FFPE block from each patient was predicated on the ability to obtain sufficient and high-quality proteins, nucleic acids, and mRNAs for analysis. One exact FFPE block from each patient was chosen for detection of multi-mics data. Each case was reviewed by two board-certified pathologists to confirm the assigned pathology. The present study was conducted in compliance with the ethical standards of the Helsinki Declaration II and was approved by the Institution Review Board of Fudan University Zhongshan Hospital (B2019-200R). Written informed consent was obtained from each patient before the performance of any study-specific investigation.

### Clinical data annotation

Clinical data, including sex, age, tumor grade, Breslow depth, Clark level, ulcer, tumor location, and OS time, were obtained from Zhongshan Hospital and have been summarized in Supplementary Table S1. The characteristics of our melanoma cohort reflect the general incidence of melanomas, including patient age distribution (20–97 years, with a median of 61).

### Sample preparation

FFPE specimens were prepared and provided by Zhongshan hospital. One 4 μM thick slide from each FFPE block was sectioned for hematoxylin and eosin (H&E) staining. For proteogenomic sample preparation, 10 μM thick slides were sectioned, deparaffinized with xylene, and washed with gradient ethanol. Specimens were selected according to H&E staining and scraped. All materials were aliquoted and stored at −80 °C until further processing. Each sample was assigned a new research ID and the patient’s name or medical record number used during hospitalization was de-identified.

For the WES analysis, a total quantity of 0.6 µg genomic DNA per sample was used as the input material for DNA preparation, and final products were quantified using an Agilent high sensitivity DNA assay (Agilent) on an Agilent Bioanalyzer 2100 system (Agilent Technologies, CA, USA). For library preparation of RNA sequencing, a total amount of 500 ng RNA per sample was used as the input material for the RNA sample preparations. Sequencing libraries were generated using Ribo-off^®^ rRNA Depletion Kit (H/M/R) (Vazyme #N406) and VAHTS® Universal V6 RNA-seq Library Prep Kit for Illumina (#N401-NR604). A total quantity of 1 µg peptides per sample was used as the input material for LC-MS/MS analysis, and LC-MS/MS were performed on Easy-nLC liquid chromatography system (Thermo Scientific) coupled to an Orbitrap Fusion Lumos Tribrid platform with FAIMS (Thermo Fisher Scientific). For the phosphoproteome analysis, a total of 500 µg peptides were then enriched with High-Select™ Fe-NTA Phosphopeptide Enrichment Kit (Thermo Scientific cat. A32992), following the manufacturer’s recommendations, and LC-MS/MS were performed on Easy-nLC liquid chromatography system (Thermo Scientific) coupled to an Orbitrap Fusion Lumos Tribrid platform with FAIMS (Thermo Fisher Scientific).

### Tumor cellularity and immune cell infiltration

The histology of the melanoma tumor tissues was examined using H&E-stained slides by two expert pathologists blinded to the proteomic subtypes. The tumor cases were graded using the AJCC staging system and staged using guidelines prescribed by the 8th edition. Information regarding tumor histological subtype, grade, and tumor purity was provided. Acceptable melanoma tumor tissue segments were determined by pathologists based on the percentage of viable tumor nuclei (> 80%) and necrosis (< 20%). Tumor purity was further independently evaluated in SCNA data on the 164 cancers (samples for WES analysis), using the ABSOLUTE algorithm^86^. Accordingly, the tumor purity validated by ABSOLUTE ranged from 80% to 90% (median 85%), which are in concordat with histologic assessed tumor purity.

### DNA extraction and quantification

For the WES analysis, DNA from 188 melanoma tissues were extracted according to the manufacturer’s instructions (QIAamp DNA Mini Kit; QIAGEN, Hilden, Germany). The isolated DNA quality and contamination were verified using the following methods: (1) DNA degradation and contamination were monitored on 1% agarose gels and (2) DNA concentration was measured via Qubit^®^ DNA Assay Kit in Qubit^®^ 2.0 Fluorometer (Invitrogen, CA, USA).

### Library preparation

A total quantity of 0.6 µg genomic DNA per sample was used as the input material for DNA preparation. Sequencing libraries were generated using Agilent SureSelect Human All Exon Kit (Agilent Technologies, CA, USA) following the manufacturer’s recommendations; further, index codes were added to each sample. Briefly, fragmentation was carried out by a hydrodynamic shearing system (Covaris, Massachusetts, USA) to generate 180–280 bp-fragments. Remaining overhangs were converted into blunt ends via exonuclease/polymerase activity. Adapter oligonucleotides were ligated after adenylation of the 3′-ends of the DNA fragments. DNA fragments with ligated adapter molecules on both ends were selectively enriched via a polymerase chain reaction (PCR). Thereafter, libraries were hybridized with the liquid phase of biotin-labeled probes, and magnetic beads with streptomycin were used to capture the exons of genes. Captured libraries were enriched in another PCR reaction to add index tags to prepare them for sequencing. Finally, the products were purified using AMPure XP system (Beckman Coulter, Beverly, USA) and quantified using an Agilent high sensitivity DNA assay (Agilent) on an Agilent Bioanalyzer 2100 system (Agilent Technologies, CA, USA).

### Clustering and sequencing

Clustering of the index-coded samples was performed on a cBot Cluster Generation System using a HiSeq PE Cluster Kit (Illumina) according to the manufacturer’s instructions. After cluster generation, the DNA libraries were sequenced on an Illumina NovaSeq platform and 150 bp paired-end reads were generated.

### WES quality control

The original fluorescence image files obtained from Novaseq platform are transformed to short reads (Raw data) by base calling and these short reads are recorded in FASTQ format, which contains sequence information and corresponding sequencing quality information. Sequence artifacts, including reads containing adapter contamination, low-quality nucleotides and unrecognizable nucleotide^87^, undoubtedly set the barrier for the subsequent reliable bioinformatics analysis. Hence quality control is an essential step and applied to guarantee the meaningful downstream analysis.

The steps of data processing were as follows:

1. Discard the paired reads if either one read contains adapter contamination (> 10 nucleotides aligned to the adapter, allowing ≤ 10% minimal matches).
2. Discard the paired reads if more than 10% of bases are uncertain in either one read.
3. Discard the paired reads if the proportion of low quality (Phred quality < 5) bases is over 50% in either one read.

All the downstream bioinformatics analyses were based on the high-quality clean data, which were retained after these steps. At the same time, QC statistics including total reads number, raw data, raw depth, sequencing error rate, percentage of reads with Q30 (the percent of bases with phred-scaled quality scores greater than 30) and GC content distribution were calculated and summarized. WES was conducted with mean coverage depths of 108× for tumor samples and 118× for adjacent non-tumor brain samples, which is consistent with the recommendations for WES^88^.

### Reads mapping and genomic variant calling

Valid sequencing data was mapped to the reference human genome (UCSC hg19) by Burrows-Wheeler Aligner (BWA) software to get the original mapping results stored in BAM format^89,90^. If one or one paired read(s) were mapped to multiple positions, the strategy adopted by BWA was to choose the most likely placement. If two or more most likely placements presented, BWA picked one randomly. Then, SAMtools^91^ and Picard (http://broadinstitute.github.io/picard/) were used to sort BAM files and do duplicate marking, local realignment, and base quality recalibration to generate final BAM file for computation of the sequence coverage and depth. ANNOVAR (version 2017 Jul 17, http://annovar.openbioinformatics.org/en/latest/)^92^ was performed to annotate the Variant Call Format file obtained in the previous step.

Filter conditions were set to identify the candidate genetic alterations as follows:

1. Remove mutations with coverage less than 10×;
2. Remove variant sites in dbSNP and with mutant allele frequency (MAF) > 0.001 in the 1000 Genomes databases (1000 Genomes Project Consortium) and the Novo-Zhonghua (in-house unrelated healthy individual database), but include sites with MAF ≥ 0.001 and < 0.1 with COSMIC evidence (http://cancer.sanger.ac.uk/cosmic)^93–95^;
3. Variations in the exosmic or splicing (10 bp upstream and downstream of splicing sites);
4. Remove synonymous mutations;
5. Retain the nonsynonymous SNVs if the functional predictions by PolyPhen-2, SIFT, MutationTaster and CADD all show the SNV is not benign^96–99^;

Retain genes identified by Cancer Gene Census (CGC, http://www.sanger.ac.uk/science/data/ cancer-gene-census).

### Somatic variant calling

For the 207 melanoma tumor samples without matched tumor-adjacent tissues as control, we referred to pervious published papers^100,101^ and developed a variant selection pipeline to detect somatic variant calling:

1. Known constitutional polymorphisms using known human variation databases, 1000 Genomes databases (1000 Genomes Project Consortium) and the Novo-Zhonghua (in-house unrelated healthy individual database)^94^;
2. Known somatic variation in melanoma and other common malignancies as reported in COSMIC V90^102^;
3. The presence of the same sequence changes in exome or whole genome sequencing data derived from 188 constitutional DNA samples analyzed in CGP (CGP normal panel); specifically, where the same base change was observed in at least two constitutional sample at allele fractions greater than 10% and the variant has not previously been confirmed as somatic in COSMIC or in two or more samples at < 10%;
4. Sequence context 5′ and 3′ to the reported sequence change highlighting s of homopolymer sequence that are prone to PCR slippage and artefacts altering the last base of the homopolymer or inserting the same base as the homopolymer at +1, +2 of the tracks and often present in unidirectional reads and < 10% variant allele burden;
5. Variant specific metrics to include protein annotation, sequence depth and % of reads reporting the variant allele.

### Exome-based somatic copy number alteration (SCNA) analysis

SCNA analysis was performed by following somatic copy-number variation (CNV) calling pipeline in GATK’s (GATK v 4.1.2.0) Best Practice. The results of this pipeline, segment files of every 1000, were put in GISTIC2 version 2.0^103^ to identify significantly amplified or deleted s across all samples, which could be accumulated driving s. To exclude false positives as much as possible, relatively stringent cutoff thresholds were used with parameters: -ta 0.5 -tb 0.5 -brlen 0.5 -conf 0.75. Other parameters were the same as the default values. Based on the published literature^88^, a log_2_ ratio cut-off of ± 0.3 was used to define CNV amplification and deletion.

### CNA-driven *cis* and *trans* effects

SCNAs affecting protein and phosphoprotein abundance in either “*cis*” (within the same aberrant locus) or “*trans*” (remote locus) mode were visualized using “multiOmicsViz” R package^104^. Spearman’s correlation coefficients and associated multiple-test adjusted *p* values were calculated for all CNA-protein pairs and CNA-phosphoprotein pairs, which resulted in CNA-protein pairs for 1631 genes and CNA-phosphoprotein pairs for 323 genes.

### Defining cancer-associated genes

Cancer-associated genes (CAGs) were compiled from genes defined by Bailey et al.^105^ and cancer-associated genes listed in Mertins et al.^106^ and adapted from Vogelstein et al.^107^.

### Mutational signature analysis

Mutation signatures were jointly inferred for 188 tumors using the R package “sigminer”. The sigminer approach (https://github.com/ShixiangWang/sigminer) was used to extract the underlying mutational signatures. The 96 mutation vectors (or contexts) generated by somatic SNVs based on six base substitutions (C > A, C > G, C > T, T > A, T > C, and T > G) within 16 possible combinations of neighboring bases for each substitution were used as input data to infer their contributions to the observed mutations. Sigminer using a nonnegative matrix factorization (NMF) approach was applied to decipher the 96 × 188 (i.e., mutational context-by-sample) matrix for the 30 known COSMIC cancer signatures (https://cancer.sanger.ac.uk/cosmic/signatures) and infer their exposure contributions.

### RNA-seq

RNA extraction RNA was extracted from tissues by using TIANGEN^®^ RNAprep Pure FFPE Kit (#DP439) according to the reagent protocols. For library preparation of RNA sequencing, a total amount of 500 ng RNA per sample was used as the input material for the RNA sample preparations. Sequencing libraries were generated using Ribo-off^®^ rRNA Depletion Kit (H/M/R) (Vazyme #N406) and VAHTS^®^ Universal V6 RNA-seq Library Prep Kit for Illumina (#N401-NR604) following the manufacturer’s recommendations and index codes were added to attribute sequences to each sample. The libraries were sequenced on an Illumina platform and 150 bp paired-end reads were generated.

### RNA-seq data analysis

RNA-seq raw data quality was assessed with the FastQC (v0.11.9) and the adapter was trimmed with Trim_Galore (version 0.6.6) before any data filtering criteria was applied. Reads were mapped onto the human reference genome (GRCh38.p13 assembly) by using STAR software (v2.7.7a). The mapped reads were assembled into transcripts or genes by using StringTie software (v2.1.4) and the genome annotation file (hg38_ucsc.annotated.gtf). For quantification purpose, the relative abundance of the transcript/gene was measured by a normalized metrics, FPKM (Fragments Per Kilobase of transcript per Million mapped reads). Transcripts with an FPKM score above one were retained, resulting in a total of 23,655 gene IDs. All known exons in the annotated file were 100% covered.

### Protein extraction and tryptic digestion

To prepare peptides for MS analysis, 10 μM thick slides from FFPE blocks were macro-dissected, deparaffinized with xylene, and washed with ethanol. The extracted tissues were then lysed in a buffer comprising 0.1 M Tris-HCl (pH 8.0), 0.1 M DTT, and 4% SDS at 99 °C for 30 min. The crude extract was then clarified via centrifugation at 16,000× *g* for 10 min, and the supernatant was loaded into a 10 kD Microcon filtration device (Millipore), centrifuged at 12,000× *g* for 20 min, and then washed twice with Urea lysis buffer (8 M Urea, 100 mM Tris-HCl, pH 8.0) and twice with 50 mM NH_4_HCO_3_. The samples were digested using trypsin at an enzyme to protein mass ratio of 1:25 overnight at 37 °C. Finally, the peptides were extracted and dried (SpeedVac, Eppendorf)^108^.

### Enrichment of phosphorylated peptides

For the phosphoproteomic analysis, peptides were extracted from the FFPE slides after trypsin digestion using the methods described above. The tryptic peptides were then enriched with High-Select™ Fe-NTA Phosphopeptide Enrichment Kit (Thermo Scientific cat. A32992), following the manufacturer’s recommendation. Briefly, peptides were suspended with binding/wash buffer (contained in the enrichment kit), mixed with the equilibrated resins, and incubated at 21–25 °C for 30 min. After incubation, the resins were washed thrice with binding/wash buffer and twice with water. The enriched peptides were eluted with elution buffer (contained in the enrichment kit), and dried in a SpeedVac.

### LC-MS/MS analysis

LC-MS/MS was performed on Easy-nLC liquid chromatography system (Thermo Scientific) coupled to an Orbitrap Fusion Lumos Tribrid platform with FAIMS (Thermo Fisher Scientific). The peptides were dissolved with 10 μL loading buffer (5% methanol and 0.2% formic acid), and 5 μL was loaded onto a 360 μm I.D. × 2 cm, C18 trap column at a maximum pressure 280 bar with 12 μL solvent A (0.1% formic acid in water). Peptides were separated on 150 μm I.D. × 30 cm column (C18, 1.9 μm, 120 Å, Dr. Maisch GmbH) with a linear 5%–35% Mobile Phase B (ACN and 0.1% formic acid) at 600 nL/min for 150 min. FAIMS separations were performed with the following settings: inner electrode temperature = 100 °C (except where noted), outer electrode temperature = 100 °C, FAIMS carrier gas flow = 2.3 L/min.

The dispersion voltage (DV) was set at −5000 V, and the compensation voltage was stepped into 40 V, 55 V and 70 V.

For MS scans, Orbitrap (OT) was utilized as detector, with resolution 120,000, scan range 300–1500 *m*/*z*, AGC target 3.0e5, Maximum Injection Time 50 ms, charge state 2–7, and data type Profile. For MS/MS scans, Ion Trap (IT) was utilized as detector, AGC target 1.0e4, Maximum Injection Time 80 ms, normalized collision energy of 30%). The dynamic exclusion time of previously obtained precursor ions was 45 s, cycle time = 1 s.

### Peptide and protein identification

MS raw files were processed with “Firmiana” (a one-stop proteomic cloud platform^109^ against the human RefSeq protein database (updated on 04-07-2013) in the National Center for Biotechnology Information. The maximum number of missed cleavages was set to two. A mass tolerance of 20 ppm for precursor and 0.5 Da for production was allowed. The fixed modification was carbamidomethyl (C), and the variable modifications were N-acetylation and oxidation of methionine. For the quality control of protein identification, a target-decoy-based strategy was applied to control the FDR of both the peptides and proteins to less than 1%. Percolator was used to obtain the probability value (*q* value), and to validate the FDR (measured by the decoy hits) of every peptide-spectrum match (PSM) lower than 1%. Thereafter, all the peptides with lengths shorter than seven amino acids were removed. The cutoff ion score for peptide identification was 20. All PSMs in all fractions were combined for protein quality control, which is a more stringent quality control strategy. The *q* values of both the target and decoy peptide sequences were dynamically increased until the corresponding protein FDR was less than 1% using the parsimony principle.

For the phosphoproteomic data, a label-free based identification analysis was performed by Proteome Discover (version 2.3). The maximum number of missed cleavages was set to 2. A mass tolerance of 20 ppm for precursor and 0.5 Da for production was allowed. The fixed modification was carbamidomethyl (C), and the variable modifications were oxidation (M), acetylation (protein N-term), and phospho (S/T/Y). The cutoff FDR, using a target-decoy strategy, was set at 1% for both the proteins and peptides.

### MS quantification of proteins and phosphoproteins

For the proteomic data, Firmiana was employed for protein quantification, and both the results and raw data from the mzXML file were loaded. Next, for each identified peptide, the extracted-ion chromatogram (XIC) was extracted by searching against the MS1 based on its identification information, and the abundance was estimated by calculating the area under the extracted XIC curve. For the protein abundance calculation, the non-redundant peptide list was used to assemble the proteins by following the parsimony principle. Thereafter, the protein abundance was estimated with a traditional label-free, intensity-based absolute quantification (iBAQ) algorithm, which divided the protein abundance (derived from intensities of the identified peptides) by the number of theoretically observable peptides^110,111^. The fraction of total (FOT), a relative quantification value that was defined as a protein’s iBAQ divided by the total iBAQ of all identified proteins in one experiment, was calculated as the normalized abundance of a particular protein in the experiments. Finally, the FOT was further multiplied by 1e6 for the ease of presentation, and NA values were replaced with 1e−5 to adjust extremely small values.

For the phosphoproteomic data, the intensities of the phosphopeptides were extracted from the Proteome Discover (version 2.3). For the phosphoprotein abundance calculation, the non-redundant phosphopeptide list was used to assemble the proteins by following the parsimony principle. Next, the phosphoprotein abundance was estimated by a traditional label-free, iBAQ algorithm, which divided the protein abundance (derived from the intensities of the identified peptides) by the number of theoretically observable peptides^108^. For phosphosite localization, the ptmRS^112^ was used to determine phosphosite confidence and phosphosite probability > 0.75 is considered as confident phosphosites.

### Quality control of the MS data

For the quality control of MS performance, the HEK293T cell (National Infrastructure Cell Line Resource) lysate was measured every 3 days as the quality control standard. The quality control standard was digested and analyzed using the same method and conditions as that of the 10 samples. A pair-wise Spearman’s correlation coefficient was calculated for all quality control runs in the statistical analysis environment R (version 4.0.0), and the results are shown in Supplementary Fig. S1d. The average correlation coefficient among the standards was 0.9, and the maximum and minimum values were 0.92 and 0.88, respectively. The result demonstrated the consistent stability of the MS platform.

### Data filtering and missing data imputation

Four pathological types of melanomas, nevi, cutaneous melanomas, acral melanomas, and mucosal melanomas, were included in this study. Before performing any downstream analysis, the proteins and phosphosites with more than 50% missing rates observed in at least one of the subtypes were filtered out. In total, 11,206 proteins and 25,318 phosphosites (belonging to 4922 phosphoproteins) were identified.

K-nearest neighbor (k-NN) imputation was applied to impute the missing values. The imputation method was implemented in the pamr package in R. Specifically, for proteins expressed in at least 50% of samples in each histological subtype, we performed KNN imputation separately on the data from each histological subtype using the “impute.knn” function from the “impute” R package^113,114^. After merging the data across histological subtypes and again replacing the missing data structure with the KNN imputation algorithm.

### Survival analysis

All the survival analysis presented in this manuscript (e.g., OS and PFS of the proteomic and phosphoproteomic sub-types, etc.), were based on Kaplan–Meier survival curves (log-rank test)^115^.

### Pathway enrichment analysis

Pathway enrichment analysis was performed by DAVID (https://david.ncifcrf.gov/) and ConsensusPathwayDB (http://cpdb.molgen.mpg.de/), and the significance of the pathway enrichment analysis was determined by Fisher’s exact test on the basis of KEGG pathways and categorical annotations, including the GO “biological process” term and Reactome (https://reactome.org/).

### Functional enrichment analysis of proteome data using GSVA/ssGSEA analysis

To further analyze biological characteristics of different samples, we performed single-sample gene set enrichment (ssGSEA/GSVA) analysis. Gene expression data of proteome across different samples were used to achieve enrichment scores over ontology gene sets (browse 14,998 gene sets) with at least 10 overlapping genes and the R/Bioconductor package GSVA. The significance of the pathway enrichment scores (PES) over different samples was estimated by linear model and moderated with the F-statistic using the R/Bioconductor package limma. The resulting significant PES among different samples were corrected by the Benjamin–Hochberg method, which used an adjusted *p* value cut-off of 0.05.

### mRNA–protein correlation

Spearman correlation coefficients and the corresponding *p* values of shared 4429 mRNA/protein pairs detected in all samples at both mRNA and protein levels were calculated across melanoma samples with RNA-Seq and MS data. In addition, the corresponding *p* values were adjusted by the Benjamini-Hochberg correction and a cut-off of 0.05 was determined as the significance of correlation pairs. In order to explore the biological functions of different expression correlations, pathway enrichment analysis was performed by DAVID.

### Investigation of proteins associated with SBS7a mutation signature

To identify proteins that altered in patients with SBS7a mutation signature, we followed published research^78,116^ and performed a regression analysis to compare the SBS7a^+^ group and SBS7a^–^ group, with the age of diagnosis, gender, and pathological stage as covariates. In total, 313 proteins were identified to be differentially expressed between SBS7a^+^ group and SBS7a^–^ group (Supplementary Table S1).

### Global heatmap

Two-way hierarchical clustering was applied to the global proteomic data of the samples and proteins to identify the global differential protein expression and protein coexpression patterns. Each gene expression value in the global proteomic expression matrix was transformed to a *z*-score across all the samples. For the sample-wise and protein-wise clustering, distance was set as “Euclidean distance”, and weight method was “complete”. The *z*-score-transformed matrix was clustered using the “pheatmap” (version 1.0.12) R package.

### Kinase activity prediction via PTM-SEA

Kinase activity scores were inferred from phosphorylation sites by employing PTM signature enrichment analysis (PTM-SEA) using the PTM signatures database (PTMsigDB) v1.9.0 (https://github.com/broadinstitute/ssGSEA2.0). Sequence windows flanking the phosphorylation site by 7 amino acids in both directions were used as unique site identifiers. Only fully localized phosphorylation sites as determined by Spectrum Mill software were taken into consideration. Phosphorylation sites on multiply phosphorylated peptides were resolved using the approach described in Krug et al.^117^ resulting in a total of 27,849 phosphorylation sites that were subjected to PTM-SEA analysis using the following parameters:

gene.set.database = “ptm.sig.db.all.flanking.human.v1.9.0.gmt”

sample.norm.type = “rank”

weight = 0.75

statistic = “area.under.RES”

output.score.type = ”NES”

nperm = 1000

global.fdr = TRUE

min.overlap = 5

correl.type = “z.score”

### TF activity inference

TF activities for tumors were computed using ssGSEA via the GSVA package^118^. TF targets obtained from DoRothEA (v1.6.0)^119^ were set as background.

### Consensus clustering analyses

We chose the top 1000 most varied proteins from the tumor tissues for subgrouping. K-means consensus clustering was applied to the selected proteins to generate subgroups. Consensus clustering was implemented on these differentially expressed proteins using the “ConsensusClusterPlus” R package^120^, and the following detailed settings were used: number of repetitions = 1000 bootstraps, pItem = 0.8 (resampling 80% of any sample), pFeature = 0.8 (resampling 80% of any protein), and k-means clustering with up to 10 clusters. The number of clusters was determined by three factors based on a previous paper^104^. We selected three clusters as the best solution for the consensus matrix since *k* = 3 provided the clearest separation among the clusters. Additionally, the consensus CDF and delta plots showed a significant increase in the area for *k* = 3 than that in *k* = 2, whereas a smaller increase was observed in the area for *k* = 3 compared with that in *k* = 4 or *k* = 5. Based on this, the melanoma proteomic data were clustered into three groups (Supplementary Fig. S3a, b).

### Estimation of stromal and immune scores

ESTIMATE^121^ and XCell^122^ were used to infer immune scores based on the transcriptome data.

### IHC

Formalin-fixed, paraffin-embedded tissue sections of 10 µM thickness were stained in batches for detecting MCM2, CDK4, RCOK2, CD8, CD163, and IL17D in a central laboratory at the Zhongshan Hospital according to standard automated protocols. Deparaffinization and rehydration were performed, followed by antigen retrieval and antibody staining. IHC was performed using the Leica BOND-MAX auto staining system (Roche). Antibody was introduced, followed by detection with a Bond Polymer Refine Detection DS9800 (Bond). Slides were imaged using an OLYMPUS BX43 microscope (OLYMPUS) and processed using a Scanscope (Leica).

### Macrophage polarization in melanoma

Microphage polarization signatures were constructed with ssGSEA^118^ using RNaseq measurements based on genes described in recent literature^122–124^. Specifically, the following gene sets were considered: Proinflammatory (M1) = (IL1B, TLR4, TNF, NOS2, APOE, CLEC7A, LGALS3, GPNMB, ITGAX, SPP1, CCL2, FABP5, CYBB); Anti-inflammatory (M2) = (COQ7, IL4, IL13, IL10, ARG1, TGFB1, SMAD3, HEXB, P2RY12, MERTK, ENTPD1, TMEM119, TGFBR1, CD163, CD206). M2-0.65*M1 difference was used for Supplementary Fig. S4c.

### Cell cycle analysis

Multi-Gene Proliferation Scores (MGPS) were calculated from the median-MAD normalized RNA-seq data as described previously^125^. Briefly, MGPS was calculated as the mean expression level of all cell cycle-regulated genes identified by Whitfield et al.^49^ in each sample. Apoptosis and E2F TG scores were the ssGSEA normalized enrichment scores from the corresponding MSigDB Hallmark gene sets calculated above (Pathway projection using ssGSEA).

### Identification of immune clusters based on cell type composition

The abundance of 64 different cell types in 75 primary melanomas was computed via xCell. For this analysis, the mRNA expression matrix, excluding > 30% missing values across all the samples, was utilized. Consensus clustering was performed based on cells only detected in at least 30% of patients (adjusted *p* < 0.01). This filtering resulted in 36 cell types. To identify sample groups with similar immune/stromal characteristics, consensus clustering was performed using the R packages ConsensusClusterPlus based on the normalized *Z*-score of these 36 xCell signatures selected above. Specifically, 80% of the original 75 samples were randomly subsampled without replacement and partitioned into three major clusters using the Patitioning Around Medoids (PAM) algorithm, which was repeated 200 times^120^.

### Multivariate COX regression analysis

The multivariate COX regression analysis was conducted, accounting for the baseline of our cohort, including, age, gender, clinical variables such as histology typing, pathological subtyping, tumor site, Clark level, ulcer, and our prognostic relevant findings, including the SBS7a^+^ mutational signature, *PRKDC* amplification status, MTHFD2, and TYM protein expression, as well as proteomic and immune classifiers. The results emphasized the findings in our study could serve as an independent predictive factor in the multivariable analysis after adjusting for clinical stage and covariates (Supplementary Table S2).

### PDC proteome

For the proteomic analysis of patient-derived cells (PDCs), Cells were lysed in lysis buffer (8 M Urea, 100 mM Tris Hydrochloride, pH 8.0) containing protease and phosphatase Inhibitors (Thermo Scientific) followed by 1 min of sonication (3 s on and 3 s off, amplitude 25%). The lysate was centrifuged at 14,000× *g* for 10 min and the supernatant was collected as whole tissue extract. Protein concentration was determined by Bradford protein assay. Extracts from each sample (500 μg protein) was reduced with 10 mM dithiothreitol at 56 °C for 30 min and alkylated with 10 mM iodoacetamide at room temperature (RT) in the dark for additional 30 min. Samples were then digested using the filter aided proteome preparation (FASP) method with trypsin. Briefly, samples were transferred into a 30 kD Microcon filter (Millipore) and centrifuged at 14,000× *g* for 20 min. The precipitate in the filter was washed twice by adding 300 μL washing buffer (8 M urea in 100 mM Tris, pH 8.0) into the filter and centrifuged at 14,000× *g* for 20 min. The precipitate was resuspended in 200 μL 100 mM NH_4_HCO_3_. Trypsin with a protein-to-enzyme ratio of 50:1 (w/w) was added into the filter. Proteins were digested at 37 °C for 16 h. After tryptic digestion, peptides were collected by centrifugation at 14,000× *g* for 20 min and dried in a vacuum concentrator (Thermo Scientific), and dried.

### Cell viability analysis

The inhibitory effects of Nedisertib (PRKDC inhibitor) and Palbociclib (CDK4 inhibitor) (purchase from Selleck Chemicals, Houston, TX, USA) on the viability of PDCs from melanoma patients were measured by the CCK-8 assay (Sigma Aldrich, USA) according to the protocol provided by the manufacturer. Briefly, cells were seeded in 96-well plates (Corning Incorporated, Corning, MA, USA) at a density of about 5 × 10^3^ cells/dish in 100 μL of culture media and grown at 37 °C for 24 h. Thereafter, they were treated with different concentrations of inhibitors for 48 h under normoxic or hypoxic conditions, respectively. Subsequently, 10 μL CCK-8 solution was added to each well and the plates were incubated at 37 °C for 0–4 h. The optical density of each well was determined at 450 nm with a microplate reader (Bio-Rad, Hercules, CA, USA). All experiments were independently repeated three times. The IC_50_ values of Nedisertib and Palbociclib in PDCs from melanoma patients were calculated using GraphPad Prism 8 software.

### Cell culture

Human malignant melanoma cells (A375 and HMCB) were obtained from ATCC and their authentication was confirmed by DNA fingerprinting with small tandem repeat (STR) profiling. A375 and HMCB cells were cultured in DMEM supplemented with 10% fetal bovine serum (FBS) (Biochrom), 100 units/mL penicillin (Invitrogen), and 100 mg/mL streptomycin (Invitrogen). Media for metabolite profiling experiments used dialyzed fetal bovine serum (FBS), prepared by dialyzing FBS for 72 h using SnakeSkin Dialysis Tubing, 3.5 K MWCO, 22 mm (Thermo Fisher) against a tenfold higher volume of phosphate-buffered saline (PBS) with a complete PBS exchange every 6 h.

### Overexpression of genes

Lenti-ORF clone of MTR was bought for origene (CAT＃: RC214275L4), full length human MTHFD2 or SHMT2 or TYMS was cloned into pLVX-IRES-Puro, the primers used were as follows:

MTHFD2-Forward (5’ to 3’):

GGATCTATTTCCGGTGAATTCATGAAACCAGCTTCAATTTCAGAGG

MTHFD2- Reverse (5’ to 3’):

GGGATCCGCGGCCGCTCTAGAATTAGTGGCTACCCCAAGCTCTT

SHMT2-Forward (5’ to 3’): GGATCTATTTCCGGTGAATTCATGCTGTACTTCTCTTTGTTTTGGG

SHMT2- Reverse (5’ to 3’):

GGGATCCGCGGCCGCTCTAGAATGCTCATCAAAACCAGGCATG

TYMS- Forward (5’ to 3’):

GGATCTATTTCCGGTGAATTCATGCCTGTGGCCGGCTCG

TYMS- Reverse (5’ to 3’):

GGGATCCGCGGCCGCTCTAGAAACAGCCATTTCCATTTTAATAGTTG

Retrovirus in supernatants was generated by using Lipofectamine 2000 transient transfection. A375 and HMCB cells were infected with retrovirus by spin infection (2250 rpm for 30 min) in polybrene. After 24 h, A375 cells were selected with 2 μg/mL puromycin.

### Cell proliferation assay

For cell growth assays, A375 and HMCB cells were plated in 96-well plate (2 × 10^3^ cells/well). CCK-8 solution (C0039, Beyotime Biotechnology) was added to the wells for 2 h and the absorbance was measured at 450 nm.

### Cell cycle detection

A375 and HMCB cells were synchronized in G1/S phase via the double thymidine block method. Briefly, A375 cells of 20%–25% density were cultured with 2 mM thymidine medium for 18 h, washed twice with PBS, and placed in fresh 10% FBS DMEM for 9 h. For the second block, cells were cultured with 2 mM thymidine medium for another 17 h. After release from the block, cells could be harvested for flow cytometric analysis every 2 h for up to 14 h for whole cell cycle detection.

### RNA interference

MTR knockdown was carried out using synthetic siRNA oligonucleotides synthesized by Genepharma. We employed two effective target sequences to exclude off-target effects. For each siRNA, a scrambled siRNA was used as a control. Transfections were performed by using RNAiMax (Invitrogen). The knockdown efficiency was verified by q-RT-PCR. Targets sequences of MTR knockdown are listed below:

MTR-KD-1: 5’- GGGATGAGATCAATGCCATTCTGCA-3’

MTR-KD-2: 5’- CAAGGCAGCCTTGTTTGCACTCCAA-3’

### Steady-state and labeling metabolite profiling

Cells were evenly seeded at 400,000 cells per well of a 6-well plate and allowed to attach for 24 h. Cells were pretreated with 10 μM compound or an equivalent volume of DMSO in DMEM for 1 h before labeling. For steady-state metabolite concentrations, cells were washed with PBS twice before pretreatment and treatment in DMEM lacking serine and glycine. For labeling experiments, [U-13C] serine replaced the unlabeled DMEM component. cells were treated with cold aqueous methanol solution (80% v/v) to quickly stop cell metabolism. Samples were then centrifuged for 15 min at 15,000× *g* and 4 °C, after which the supernatants were collected. The supernatants were then lyophilized and stored at −80 °C until analysis.

### LC-MS/MS analysis of metabolics

Dried samples were resuspended in 500 μL methanol/water (10:90 v/v) with 0.01 ng/mL Val-d8 and Phe-d8 used as internal extraction standards. The separated metabolites were acquired by performing high-performance liquid chromatography (HPLC) using an LC-20AB pump (Shimadzu, Kyoto, Japan) and a Luna NH2 column (P/N 00 B - 4378-B0; 5 μm, 50 × 2.0 mm; Phenomenex, Torrance, CA). The mobile phase comprised eluent A (0.77 g NH_4_OAc, 1.25 mL NH_4_OH, 25 mL ACN, and 300 µL acetic acid^62^ dissolved in 500 mL water) and eluent B (ACN). The elution program was as follows: 0.1 min, 85% B; 3 min, 30% B; 12 min, 2% B; 15 min, 2% B; and 16–28 min, 85% B. The flow rate of the pump was 0.3 mL min^−1^ and the mass spectrometer used was the Orbitrap Exactive (Thermo Scientific); the MS parameters were positive voltage of 4.5 kV and negative voltage of 3.5 kV. Metabolites were monitored using a polarity switching full-scan method and were identified by performing accurate mass measurements (± 10 ppm). Furthermore, validation was performed by concordance with the standard retention times within 15 s. The ions arising from TMP were monitored at 321–195, methionine ions were monitored at 150–103.8, dUMP ions were monitored at 307–195, SAM ions were monitored at 399–250, and SAH ions were monitored at 385–136. Metabolite peaks were identified and integrated using the Xcalibur v.2.2 software (Thermo Fisher Scientific) and normalized to internal standards and total cell volume. The m/z ratios for stable isotopically labeled metabolites were obtained by using IsoMETLIN and were corrected for natural abundance.

### Xenograft tumorigenesis assay

Four-week-old male BALB/c nude mice were purchased from the SLAC Company (Shanghai, China) and maintained in pathogen-free conditions. All animal experiments were approved by the animal care regulations of the Institutional Animal Care and Use Committee of Fudan University. All procedures were approved by IACUC, Fudan University. Ethical review approval number 2018JS0027 was obtained from the Department of experimental animal science, Fudan University. All animals were acclimated for 1 week before experiments. 5 × 10^6^ A375 cells (MTHFD2-overexpressing or MTHFD2/MTR-overexpressing or MTHFD2-overexpressing/MTR knock down) in 100 μL PBS were subcutaneously inoculated at the flanks of nude mice. After 2 weeks, the tumors were palpable, and the mice were pooled by tumor type and divided randomly to two groups, which were assigned blindly to vehicle or NCT-503 treatment. Each group contained 6 mice for a total of 48 mice in all groups. NCT-503 was prepared in a vehicle of 5% ethanol, 35% PEG300 (Sigma), and 60% of an aqueous 30% hydroxypropyl-β-cyclodextrin (Sigma) solution, and injected intraperitoneally once daily. Dose was adjusted to mouse weight, and the volume of injection did not exceed 150 μL. All mice were euthanized and tumors were harvested 6 weeks after inoculation. tumor volumes were calculated with this formula: volume = 0.52 × (width)^2^ × length.

### Western blotting

Cells were lysed in EBC lysis buffer (50 mM Tris HCl, pH 8.0, 120 mM NaCl, 0.5% Nonidet P-40) supplemented with protease inhibitors (Selleck Chemicals) and phosphatase inhibitors (Selleck Chemicals). Proteins were separated by 10% SDS-PAGE gel and blotted with indicated primary antibodies. Primary antibodies used for western blot analysis were as follows: anti-MTR (1:1000; 25896-1-AP; Proteintech), anti-BHMT (1:1000; 15965-1-AP; Proteintech), anti-MTHFD2 (1:500; #41377; CST). anti-SHMT1 (1:3000; #80715; CST), anti-TYMS (1:1000; 15047-1-AP; Proteintech), anti-TYMS (1:1000; 14513-1-AP; Proteintech) or anti-PGAM1 (1:1000; 16126-1-AP; Proteintech) were used. The western blot gel image was obtained with a Typhoon FLA 9500 scanner (GE Healthcare).

### EDU staining

Various groups of A375 and HMCB cells were cultured at the same concentration for growth and 20 μM EDU were added to the cell culture medium for 1 h. Cells were harvested and washed with PBS twice to remove the remaining medium. Paraformaldehyde (4%) was used to fix the cells at room temperature (RT); 0.5%Triton X-100 in PBS was added and incubated for 20 min at RT. The cocktail (PBS: 215 μL, 100 mM CuSO_4_: 10 μL, 2 mM Azide: 0.6 μL, 1 M Sodium Ascorbate: 25 μL) was added for 30 min at room temperature in the dark. DAPI was subsequently added, for nuclear staining. Results were acquired in flow cytometer or cells were observed under a fluorescence microscope.

### ELISPOT assay

The ELISPOT plate was processed according to the manufacturer’s instructions. Briefly, ELIIP plates (Abcam, Cat# ab62899) were pretreated with 100 mL/well of PBS for 10 min. Then, we dispense into wells 100 mL of cell suspension containing nearly 10^5^ cells. Cover the plate with a standard 96-well plate plastic lid and incubate cells at 37 °C in a CO_2_ incubator. After 20 h of co-culture, the ELISPOT plates were washed with 100 mL of PBS-0.1% tween 20 (PBS-T) in wells, and then incubated for 1 h at room temperature (RT) with 100 mL/well of anti-human IFNγ detection antibody solution. The plate was then washed 3 times with PBS-T, followed by a 1 h incubation with 100 mL/well of Streptavidin-Alkaline phosphatase. The plate was then washed 3× with PBS followed by development with 100 mL/well of ready-to-use BCIP/NBT buffer. The reaction was stopped by rinsing thoroughly with cold tap water. ELISPOT plates were scanned and counted using an ImmunoSpot plate reader and associated software (Cellular Technologies, Ltd).

### Limitations

In this study, to ensure the clinical relevance of our research, samples from patients with prognostic information were enrolled as a priority. Thus, some samples collected were too tiny to be segmented into four parts for multi-omics analysis. For those samples, only one layer of omics analysis could be performed, and since proteins were the final functional regulators, the proteomic analysis was performed.

## Supplementary information

Supplementary Information

Supplementary Table S1

Supplementary Table S2

Supplementary Table S3

Supplementary Table S4

## Data Availability

The data that support the findings of this study are available within the paper and its Supplementary Information. The proteome raw files can be obtained from the ProteomeXchange Consortium (https://proteomecentral.proteomexchange.org) with the dataset identifier [PXD052502]. All WES raw data, RNAseq raw data and phosphoproteome raw data have been deposited to NODE (https://www.biosino.org/node/) and can be accessed with the project ID: OEP002210 (WES), OEP002201 (RNAseq), and OEP001888 (phosphoproteome). The raw files of Annotated gene sets were collected from GO (https://geneontology.org/docs/download-go-annotations/). For molecular signatures database, KEGG database and Reactome database, we got access to them by the R package: msigdbr (version 7.5.1). The public data for validation were downloaded from supplementary files of published articles (10.1371/journal.pone.0010770, 10.1016/j.cell.2015.05.044, 10.1016/j.cell.2019.08.012, 10.1158/1078-0432). The information of kinase substrate relationships was available in PhosphoSite [https://www.phosphosite.org/homeAction.action], Phospho. ELM [http://phospho.elm.eu.org/dataset.html], and PhosphoPOINT [http://kinase.bioinformatics.tw/]. Software and publicly available resources used in this study were described in the Methods section. The remaining data are available within the article, Supplementary Information, or Source data file. Source data are provided with this paper.
