# Author Correction: Proteogenomic insights into the biology and treatment of pan-melanoma

**DOI:** 10.1038/s41421-025-00846-5

**Published:** 2025-11-18

**Authors:** Hang Xiang, Rongkui Luo, Yunzhi Wang, Bing Yang, Sha Xu, Wen Huang, Shaoshuai Tang, Rundong Fang, Lingli Chen, Na Zhu, Zixiang Yu, Sujie Akesu, Chuanyuan Wei, Chen Xu, Yuhong Zhou, Jianying Gu, Jianyuan Zhao, Yingyong Hou, Chen Ding

**Affiliations:** 1 State Key Laboratory of Genetic Engineering, School of Life Sciences, Human Phenome Institute, Department of Plastic and Reconstructive Surgery, Zhongshan Hospital, Fudan University, Shanghai, China; 2 Department of Pathology, Zhongshan Hospital, Fudan University, Shanghai, China; 3 State Key Laboratory of Oncology in South China, Guangdong Provincial Clinical Research Center for Cancer, Sun Yat-sen University Cancer Center, Guangzhou, Guangdong China; 4 Department of Plastic and Reconstructive Surgery, Zhongshan Hospital, Fudan University, Shanghai, China; 5 Department of Medical Oncology, Zhongshan Hospital, Fudan University, Shanghai, China; 6 Department of Plastic and Reconstructive Surgery, Zhongshan Hospital (Xiamen), Fudan University, Shanghai, China; 7 Institute for Developmental and Regenerative Cardiovascular Medicine, MOE-Shanghai Key Laboratory of Children’s Environmental Health, Xinhua Hospital, Shanghai Jiao Tong University School of Medicine, Shanghai, China

**Keywords:** Melanoma, Proteomics

Correction to: *Cell Discovery* 10.1038/s41421-024-00688-7, published online 23 July 2024

In this article, we identified an oversight in the immunohistochemistry (IHC) image in the bottom left corner in Fig. 4m, where the wrong IHC image was accidentally used. In Supplementary Fig. S2h, we identified an oversight, where the far left IHC image in MCM2_S27 (+) Tumor panel was mistakenly duplicated in the far left IHC image in CDK4_T172 (+) Tumor panel; in addition, the other two IHC images of MCM2_S27 (+) Tumor panel were incorrectly used. Also, we identified an oversight in Fig. 5k, where the representative images of OE-Ctrl-A375 cells and ROCK2-KD-A375 cells in Fig. 5h were mistakenly duplicated in the representative images of sh-Ctrl cells and sh-HMGB1 cells in Fig. 5k.

Although these inadvertent errors do not alter any of the reported results or conclusions, we nevertheless wish to uphold the strictest standards of publication integrity. The figure and the relevant Supplementary figures have now been carefully corrected. We sincerely apologize for any inconvenience this may have caused.

The original relevant figures and Supplementary figure and the corrected version are attached below.

Incorrect Figure 4

**Fig. 4:**
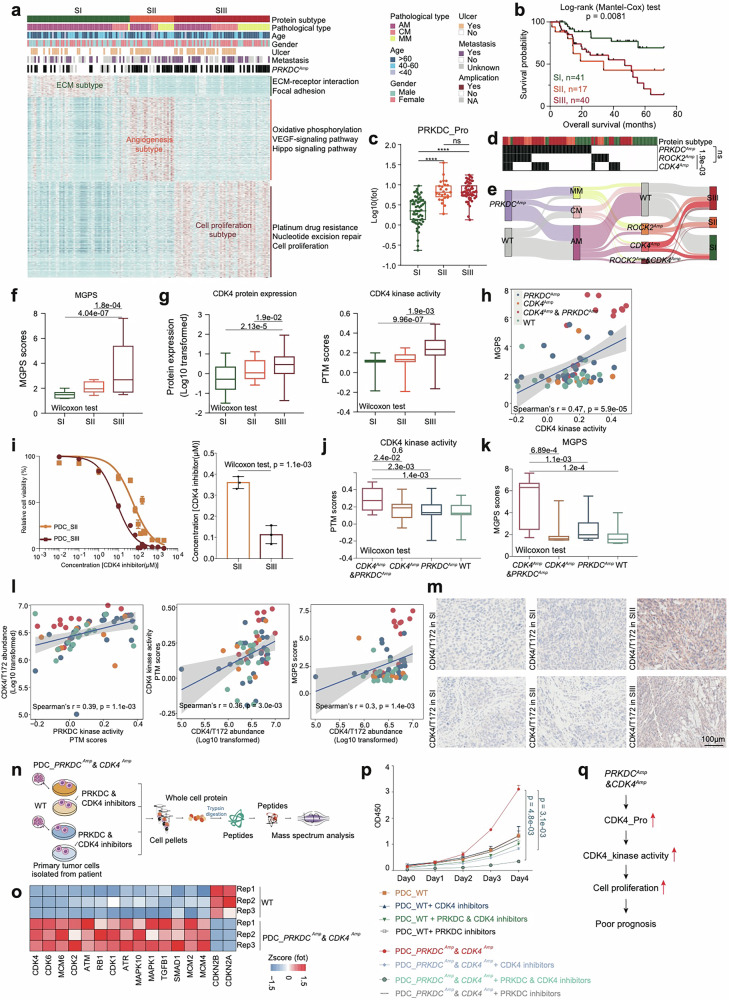
Proteomic subtypes of primary melanomas. a Heatmap illustrated clinical information, and frequency of *PRKDC* amplicons in 137 melanoma patients. The remaining section illustrates global proteomic features upregulated in the three proteomic subtypes. The pathways enriched by proteins elevated in corresponding subgroups are labeled on the right. **b** The association of three proteomic subtypes with clinical outcomes in melanoma patients (SI: n = 41; SII: n = 17; SIII: n = 40) (*p* value based on the log-rank test). **c** The boxplot showed the protein expression of PRKDC in the three proteomic subtypes (n = 137) (Wilcoxon rank test, ****p < 0.0001). **d** Heatmap illustrated the amplification frequency of *PRKDC*, *CDK4*, *ROCK2* in the three proteomic subtypes (Fisher’s exact test). **e** Sankey plot showed the amplification frequency of *PRKDC*, *CDK4*, and *ROCK2* in the three pathological subtypes and three proteomic subtypes of melanomas. **f** The boxplot showed the MGPS score in the three proteomic subtypes (n = 137) (Wilcoxon rank test). **g** The boxplot showed the protein expression and kinase activity of CDK4 in the three proteomic subtypes (n = 137) (Wilcoxon rank test). **h** Spearman-rank correlation of the CDK4’s kinase activity and MGPS score in melanomas (n = 96). **i** Dose–response curves of CDK4 inhibitor were determined on day 2 after inhibitors adding in PDCs from melanoma patients of SII and SIII proteomic subtypes. The data represent the mean values ± SD (n = 3) (left); IC_50_ values of CDK4 inhibitor were determined on day 2 after inhibitors adding. The data represent the mean values ± SD (n = 3) (right). **j** The boxplot showed the PTM score of CDK4 in patients harboring *CDK4* amplicons & *PRKDC* amplicons, or only *PRKDC* amplicons, or only *CDK4* amplicons and WT samples in our cohort (n = 96) (Wilcoxon rank test). **k** The boxplot showed the MGPS score in patients harboring *CDK4* amplicons & *PRKDC* amplicons, or only *PRKDC* amplicons, or only *CDK4* amplicons and WT samples in our cohort (n = 124) (Wilcoxon rank test). **l** Spearman-rank correlation of the PRKDC’s kinase activity and CDK4/T172’s abundance in melanomas (left); Spearman-rank correlation of the CDK4/T172’s abundance and CDK4’s kinase activity in melanomas (middle); Spearman-rank correlation of the CDK4/T172’s abundance and MGPS score in melanomas (right). **m** Immunohistochemistry of CDK4/T172 in SII and SIII proteomic subtype samples, scale bar = 100 μm. **n** The workflow showed the sample collection for mass spectrum analysis. **o** Heatmap illustrated the protein expression of CDK4, CDK6, et al. participating in cell cycle were upregulated in the PDCs from melanoma patients harboring *CDK4* amplicons & *PRKDC* amplicons. **p** Proliferation of the PDCs from melanoma patients with or without *PRKDC* amplification and *CDK4* amplification based on the use of PRKDC inhibitor and CDK4 inhibitor, or only CDK4 inhibitor, or only PRKDC inhibitor, or control (two-way ANOVA followed by Tukey’s multiple comparison test). The data are presented as mean ± SEM. **q** Illustration of the activation of PRKDC–CDK4 signaling pathway combined with cell proliferation led to poor prognosis in melanomas.

Correct Figure 4

**Fig. 4:**
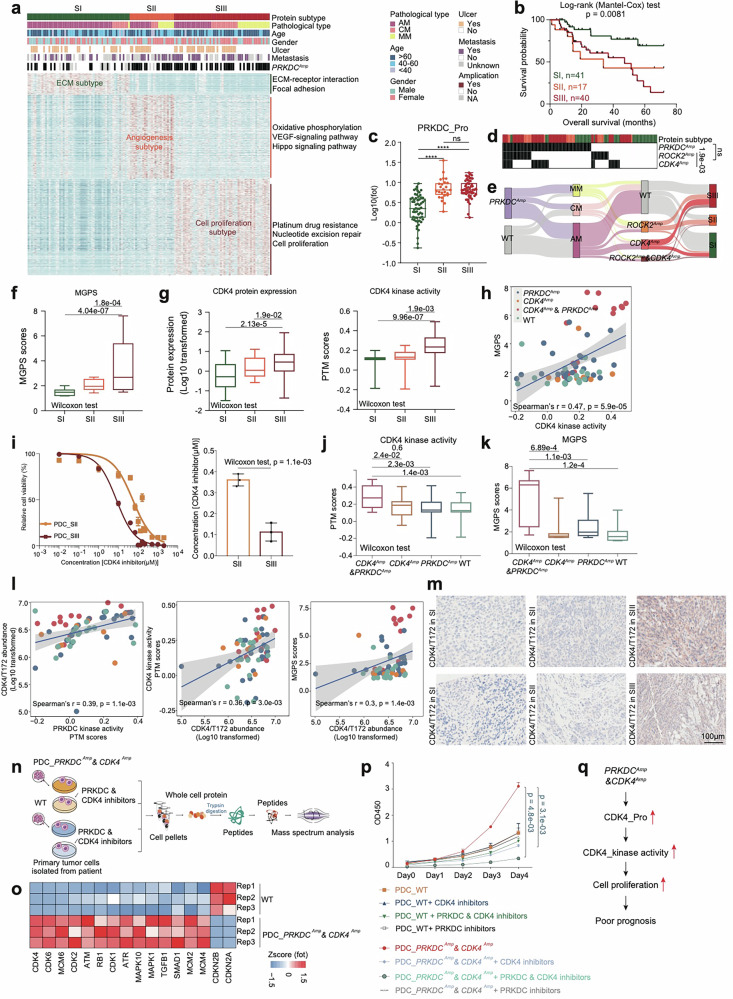
Proteomic subtypes of primary melanomas. a Heatmap illustrated clinical information, and frequency of *PRKDC* amplicons in 137 melanoma patients. The remaining section illustrates global proteomic features upregulated in the three proteomic subtypes. The pathways enriched by proteins elevated in corresponding subgroups are labeled on the right. **b** The association of three proteomic subtypes with clinical outcomes in melanoma patients (SI: n = 41; SII: n = 17; SIII: n = 40) (*p* value based on the log-rank test). **c** The boxplot showed the protein expression of PRKDC in the three proteomic subtypes (n = 137) (Wilcoxon rank test, ****p < 0.0001). **d** Heatmap illustrated the amplification frequency of *PRKDC*, *CDK4*, *ROCK2* in the three proteomic subtypes (Fisher’s exact test). **e** Sankey plot showed the amplification frequency of *PRKDC*, *CDK4*, and *ROCK2* in the three pathological subtypes and three proteomic subtypes of melanomas. **f** The boxplot showed the MGPS score in the three proteomic subtypes (n = 137) (Wilcoxon rank test). **g** The boxplot showed the protein expression and kinase activity of CDK4 in the three proteomic subtypes (n = 137) (Wilcoxon rank test). **h** Spearman-rank correlation of the CDK4’s kinase activity and MGPS score in melanomas (n = 96). **i** Dose–response curves of CDK4 inhibitor were determined on day 2 after inhibitors adding in PDCs from melanoma patients of SII and SIII proteomic subtypes. The data represent the mean values ± SD (n = 3) (left); IC_50_ values of CDK4 inhibitor were determined on day 2 after inhibitors adding. The data represent the mean values ± SD (n = 3) (right). **j** The boxplot showed the PTM score of CDK4 in patients harboring *CDK4* amplicons & *PRKDC* amplicons, or only *PRKDC* amplicons, or only *CDK4* amplicons and WT samples in our cohort (n = 96) (Wilcoxon rank test). **k** The boxplot showed the MGPS score in patients harboring *CDK4* amplicons & *PRKDC* amplicons, or only *PRKDC* amplicons, or only *CDK4* amplicons and WT samples in our cohort (n = 124) (Wilcoxon rank test). **l** Spearman-rank correlation of the PRKDC’s kinase activity and CDK4/T172’s abundance in melanomas (left); Spearman-rank correlation of the CDK4/T172’s abundance and CDK4’s kinase activity in melanomas (middle); Spearman-rank correlation of the CDK4/T172’s abundance and MGPS score in melanomas (right). **m** Immunohistochemistry of CDK4/T172 in SII and SIII proteomic subtype samples, scale bar = 100 μm. **n** The workflow showed the sample collection for mass spectrum analysis. **o** Heatmap illustrated the protein expression of CDK4, CDK6, et al. participating in cell cycle were upregulated in the PDCs from melanoma patients harboring *CDK4* amplicons & *PRKDC* amplicons. **p** Proliferation of the PDCs from melanoma patients with or without *PRKDC* amplification and *CDK4* amplification based on the use of PRKDC inhibitor and CDK4 inhibitor, or only CDK4 inhibitor, or only PRKDC inhibitor, or control (two-way ANOVA followed by Tukey’s multiple comparison test). The data are presented as mean ± SEM. **q** Illustration of the activation of PRKDC–CDK4 signaling pathway combined with cell proliferation led to poor prognosis in melanomas.

Incorrect Figure 5

**Fig. 5:**
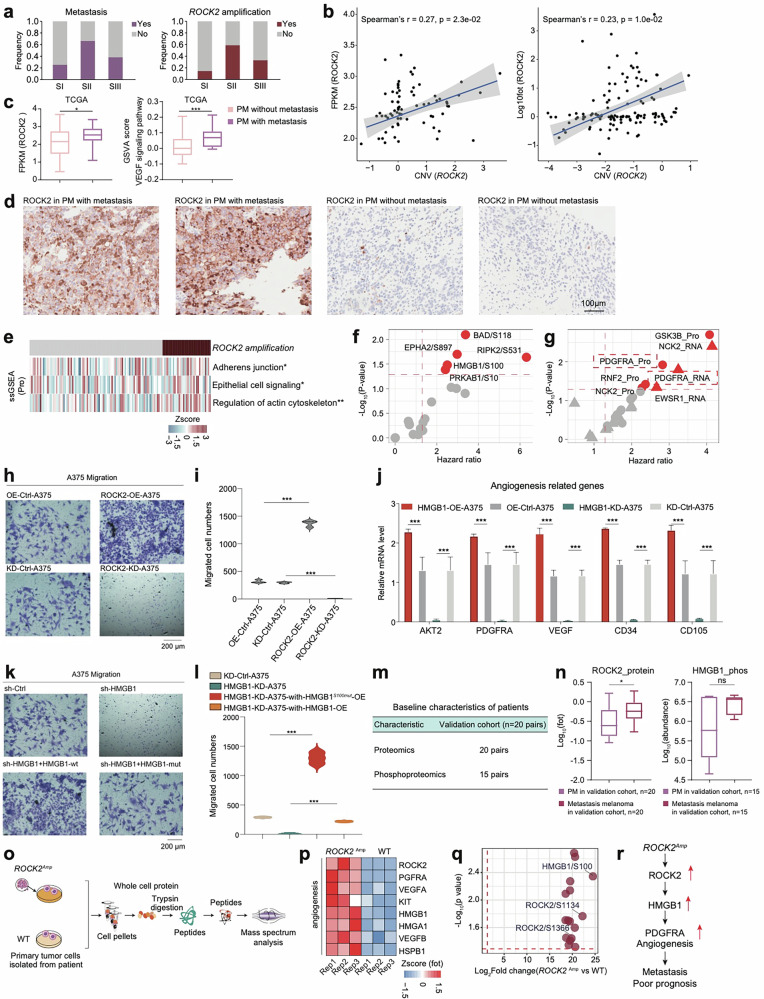
***ROCK2*** amplification promoting the metastasis in melanomas. **a** The histogram showed the frequency of metastasis (n = 79) and *ROCK2* amplification (n = 124). **b** Spearman-rank correlation of the *ROCK2*’s copy number and *ROCK2*’s mRNA expression in melanomas (n = 73) (left); Spearman-rank correlation of the *ROCK2*’s copy number and *ROCK2*’s protein expression in melanomas (n = 124) (right). **c** The boxplot showed the mRNA expression of ROCK2 and the GSVA score of VEGF signaling pathway in primary melanomas with or without metastasis in TCGA cohort (n = 267). **d** Immunohistochemistry of ROCK2 in primary melanomas with or without metastasis, scale bar = 100 μm. **e** The heatmap depicted the pathways significantly elevated in samples harboring ROCK2 amplificon (*p < 0.05, **p < 0.01, ***p < 0.001, Wilcoxon rank test). **f** The volcano plot showed the abundance of the phosphosites predictive of OS in melanomas. **g** The volcano plot showed the expression of HMGB1’s TGs predictive of OS in melanomas. **h** The effects of ROCK2 on the migration of A375 cells were confirmed by transwell. **i** The violin plots (right panel) indicated counts of migrated A375 cells under different treatments. **j** The boxplot indicated the expression level angiogenesis-related genes across OE-Control-A375, OE-HMGB1-A375, KD-Control-A375, and HMGB1-KD-A375. **k** The effects of HMGB1 on the migration of A375 cells were confirmed by transwell. **l** The violin plots (right panel) indicated counts of migrated A375 cells under different treatments. **m** The table showed the baseline characteristics of patients in the validation cohort1. **n** The boxplot showed the protein expression of ROCK2 and the phosphorylate (n = 20) ion abundance of HMGB1 in paired primary melanomas and paired metastasis melanomas in validation corhot1 (*p < 0.05, ****p < 0.0001, Wilcoxon rank test). **o** The workflow showed the sample collection for mass spectrum analysis. **p** Heatmap illustrated the protein expression of ROCK2, VEGFRA, HMGB1, et al. participating in angiogenesis were upregulated in the PDCs from melanoma patients harboring *ROCK2* amplicons. **q** The volcano plot showed the significantly upregulated phosphorylation in the melanoma patients harboring *ROCK2* amplicons. **r** The systematic diagram summarized cascading regulatory role of ROCK2 on angiogenesis, and promoting melanoma metastasis through HMGB1.

Correct Figure 5

**Fig. 5:**
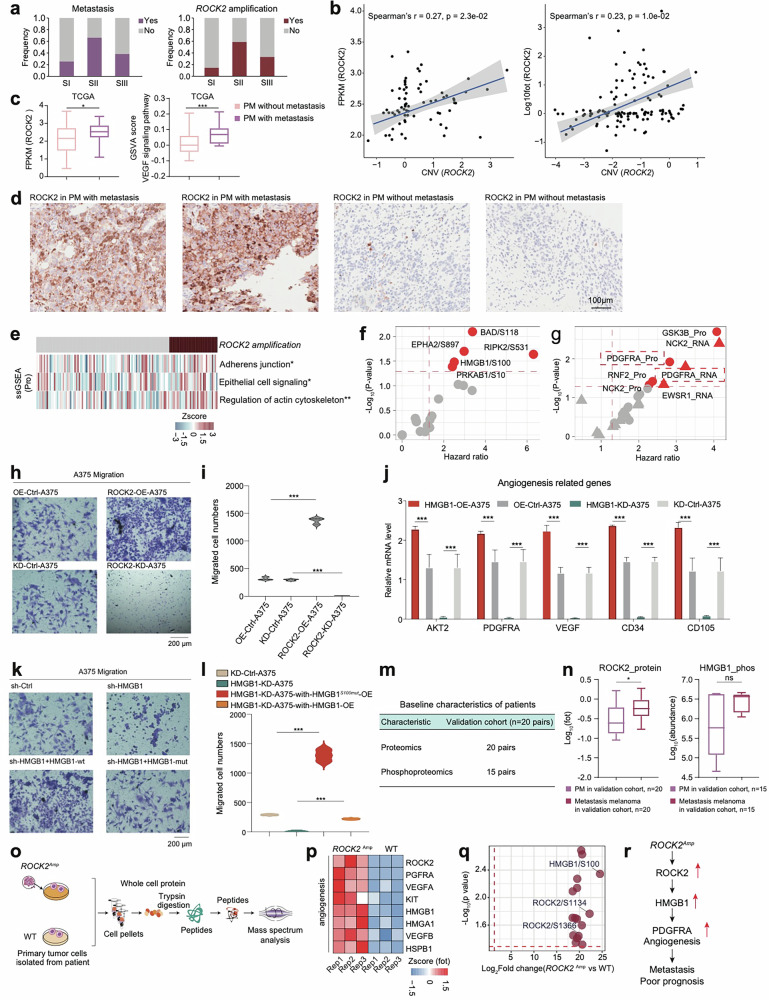
*ROCK2* amplification promoting the metastasis in melanomas. a The histogram showed the frequency of metastasis (n = 79) and *ROCK2* amplification (n = 124). **b** Spearman-rank correlation of the *ROCK2*’s copy number and *ROCK2*’s mRNA expression in melanomas (n = 73) (left); Spearman-rank correlation of the *ROCK2*’s copy number and *ROCK2*’s protein expression in melanomas (n = 124) (right). **c** The boxplot showed the mRNA expression of ROCK2 and the GSVA score of VEGF signaling pathway in primary melanomas with or without metastasis in TCGA cohort (n = 267). **d** Immunohistochemistry of ROCK2 in primary melanomas with or without metastasis, scale bar = 100 μm. **e** The heatmap depicted the pathways significantly elevated in samples harboring ROCK2 amplificon (*p < 0.05, **p < 0.01, ***p < 0.001, Wilcoxon rank test). **f** The volcano plot showed the abundance of the phosphosites predictive of OS in melanomas. **g** The volcano plot showed the expression of HMGB1’s TGs predictive of OS in melanomas. **h** The effects of ROCK2 on the migration of A375 cells were confirmed by transwell. **i** The violin plots (right panel) indicated counts of migrated A375 cells under different treatments. **j** The boxplot indicated the expression level angiogenesis-related genes across OE-Control-A375, OE-HMGB1-A375, KD-Control-A375, and HMGB1-KD-A375. **k** The effects of HMGB1 on the migration of A375 cells were confirmed by transwell. **l** The violin plots (right panel) indicated counts of migrated A375 cells under different treatments. **m** The table showed the baseline characteristics of patients in the validation cohort1. **n** The boxplot showed the protein expression of ROCK2 and the phosphorylate (n = 20) ion abundance of HMGB1 in paired primary melanomas and paired metastasis melanomas in validation corhot1 (*p < 0.05, ****p < 0.0001, Wilcoxon rank test). **o** The workflow showed the sample collection for mass spectrum analysis. **p** Heatmap illustrated the protein expression of ROCK2, VEGFRA, HMGB1, et al. participating in angiogenesis were upregulated in the PDCs from melanoma patients harboring *ROCK2* amplicons. **q** The volcano plot showed the significantly upregulated phosphorylation in the melanoma patients harboring *ROCK2* amplicons. **r** The systematic diagram summarized cascading regulatory role of ROCK2 on angiogenesis, and promoting melanoma metastasis through HMGB1.

Correct Supplementary Figure 2
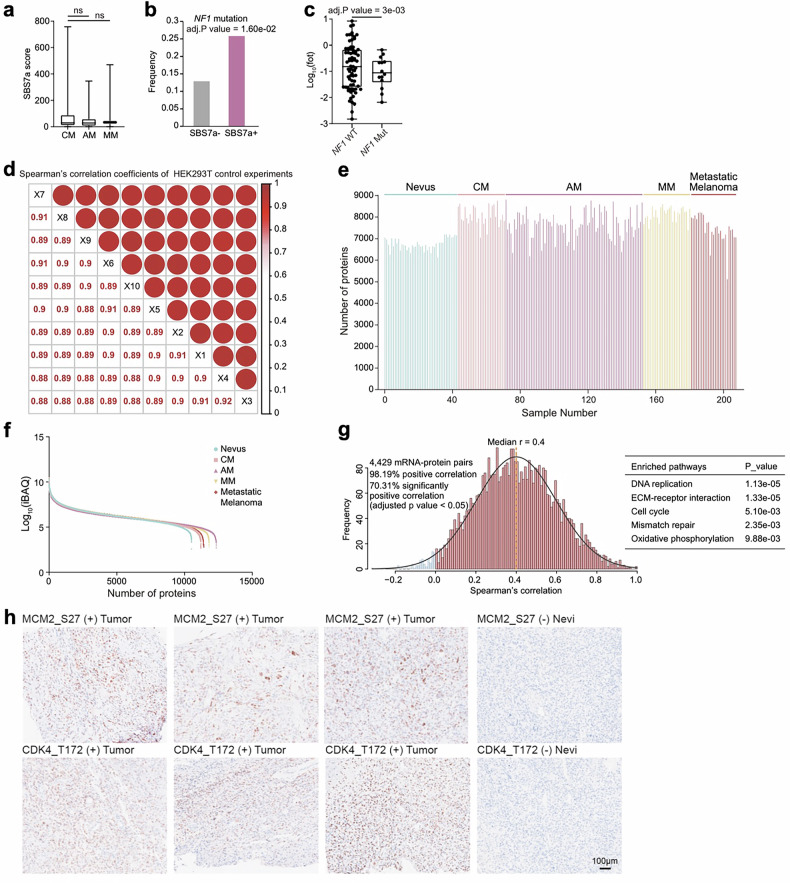


**Figure S2**. Quality Control, and Comparison of Somatic Mutation Profiles between Four Types of Melanomas, Related to Figure 1.

- The boxplot showed SBS7a signature score in CM (n = 25), AM (n = 71), and MM (n = 28) (Wilcoxon rank test).
- The histogram showed the frequency of *NF1* mutation in patients with or without SBS7a signature (n = 124) (Fisher’s exact test).
- The boxplot showed the protein expression of NF1 in patients harboring *NF1* mutation and WT samples in our cohort (n = 124) (Wilcoxon rank test).
- The quantification repeatability of HEK293T control samples showing the robust and accurate proteome platform (Pearson’s correlation coefficients, 0.88-0.92).
- Number of proteins identified in melanoma patients.
- Dynamic range of Nevus (n = 43), CM (n = 28), AM (n = 81), MM (n = 28) and MCM (n = 27) samples.
- Correlations between mRNA and protein abundance in 4,429 mRNA-protein pairs detected in all samples.
- IHC staining CDK4 at T172, and MCM2 at S27 in melanoma tumor tissues and nevi. FFPE sections were stained for phosphorylation of CDK4 at T172 and MCM2 at S27. The scale bar indicates 100 μm.

