# Supplementary material for: Proteogenomic insights into the biology and treatment of pan-melanoma
Source: Cell Discov. 2024 Jul 23;10:78. doi: 10.1038/s41421-024-00688-7 (PMC11263678; doi:10.1038/s41421-024-00688-7)

Figure S1. Single Base Substitutions (SBS) Mutation Signatures of Melanomas, Related to Figure 1.

A. The relative mutations mutation frequencies of 96 tri-nucleotide mutation patterns are plotted with SBS30, SBS1, SBS18, and SBS7a mutation patterns in our cohort (n = 124) using three additional tools "Maftools", "MutationalPatterns", and "SigProfilerExtractor".

B. The heatmap indicated the correlation ecoefficiency among different mutational signature approaches.

C. Kaplan-Meier curves for OS based on SBS7a enrichment scores (n = 92).

D. The relative mutations mutation frequencies of 96 tri-nucleotide mutation patterns are plotted with SBS30, SBS1, SBS18, and SBS7a mutation patterns in TCGA cohort (n = 575).

E. The forest plot indicated the 95% CI of hazard ratio of SBS7a enrichment scores, in both FUDAN cohort (n = 92) and TCGA cohort (n = 575).

Figure S2. Quality Control, and Comparison of Somatic Mutation Profiles between Four Types of Melanomas, Related to Figure 1.

A. The boxplot showed SBS7a signature score in CM (n = 25), AM (n = 71), and MM (n = 28) (Wilcoxon rank test).

B. The histogram showed the frequency of *NF1* mutation in patients with or without SBS7a signature (n = 124) (Fisher's exact test).

C. The boxplot showed the protein expression of NF1 in patients harboring *NF1* mutation and WT samples in our cohort (n = 124) (Wilcoxon rank test).

D. The quantification repeatability of HEK293T control samples showing the robust and accurate proteome platform (Pearson's correlation coefficients, 0.88-0.92).

E. Number of proteins identified in melanoma patients.

F. Dynamic range of Nevus (n = 43), CM (n = 28), AM (n = 81), MM (n = 28) and MCM (n = 27) samples.

G. Correlations between mRNA and protein abundance in 4,429 mRNA-protein pairs detected in all samples.

H. IHC staining CDK4 at T172, and MCM2 at S27 in melanoma tumor tissues and nevi. FFPE sections were stained for phosphorylation of CDK4 at T172 and MCM2 at S27. The scale bar indicates 100 μm.

Figure S3. *PRKDC* Amplification Associated with Poor Prognosis in Melanomas, Related to Figure 2.

A. The boxplot indicated the mRNA expression of PRKDC between patients harbored *PRKDC* amplifications or not in TCGA cohort.

B. The bar plot indicated the percentages of *PRKDC* amplifications across diverse histological types in different cohorts.

C. The Kaplan-Meier curves for overall survival based on patients' *PRKDC* copy number alterations

D. Kaplan-Meier curves for overall survival based on patients grouped by genomic alterations in our cohort.

E. The relationship between the protein expression of MTSS1 and GSVA scores of GO process actin cytoskeleton organization.

F. The interactions among proteins enriched in actin cytoskeleton organization. Proteins were color-coded based on their correlation with MTSS1.

G. The volcano plot indicates the relationship between drugs' sensitivity and protein expression of PRKDC.

H. The boxplot indicates the protein expression of PRKDC across cell lines with different PRKDC expression levels.

I. Dose–response curves of 5-FU were determined on day 2 after inhibitors were added to cell lines. The data represent the mean values ± SD (n = 3) (left); The violin plot shows the IC_{50} scores. The data represent the mean values ± SD (n = 3) (right).

J-K The proliferation of the cell lines with different PRKDC expression levels, under 5-FU treatment (K) or not (J).

Figure S4. *PRKDC* Amplification Contributed to Expression Alterations of Proteins in Folate Metabolism, Related to Figure 3.

A. The workflow showed the sample collection for mass spectrum analysis (control HMCB cells, scramble shRNA control HMCB cells, PRKDC-OE HMCB cells, and PRKDC-KD HMCB cells).

B. The violin plots indicated the expression patterns of PRKDC across cells with different treatments.

C. The violin plots indicated the expression patterns of MXD3/S57 across cells with different treatments.

D. The regulatory role of MXD3 on MTHFD2 and TYMS.

E. Immunoblot validation of MTR, MTHFD2, TYMS, and SHMT2 expression in WT and *PRKDC* amplification melanoma tissues.

F. The heatmap indicates the expression of proteins that participated in folate metabolism.

G. Summary of folate metabolism pathway.

Figure S5. Decreased MTR Facilitates One-carbon Utilization in DNA Synthesis, Related to Figure 3.

A. Proliferation of the indicated A375 cells when MTHFD2/TYMS or an empty vector was overexpressed based on the use of MTR knockdown or control (two-way ANOVA followed by Tukey's multiple comparison test). Proliferation of A375 cells after MTR overexpression based on MTHFD2/TYMS overexpression (two-way ANOVA followed by Tukey's multiple comparisons test). The data are presented as mean ± SEM, *p < 0.05; **p < 0.01; ***p < 0.001.

B. The comparison of proliferation rates of PRKDC-OE-HMCB cells or HMCB when TYMS or MTHFD2 was overexpressed. The data are presented as mean ± SEM.

C. Metabolism of indicated A375 cells after MTR KO based on MTHFD2 or TYMS overexpression (t test). Data are represented as mean ± SEM, *p < 0.05; **p < 0.01; ***p < 0.001.

D. One-carbon metabolism of indicated A375 cells after MTR KO on MTHFD2 or TYMS overexpression (t test). Data are represented as mean ± SEM, *p < 0.05; **p < 0.01; ***p < 0.001.

E. DNA synthesis of indicated A375 cells after MTR KO on MTHFD2 or TYMS overexpression (t test) (left). Amount of G1 phage of indicated A375 cells after MTR KO on MTHFD2 or TYMS overexpression (t test) (right). Data are represented as mean ± SEM, *p < 0.05; **p < 0.01; ***p < 0.001.

Figure S6. Consensus Clustering Analysis Conducted in Melanomas, Related to Figure 4.

A-B. Heatmap of consensus cluster plus, cophenetic correlation coefficient and average silhouette width plots. The input is the quantile-normalized iBAQ intensity matrix of the top 1000 most variant proteins across 137 tumor samples. Based on a visual inspection of the hierarchical clustering and the profiles of the cophenetic correlation coefficient and average silhouette width for solutions with 2 to 5 clusters, we considered K = 3 to be the preferred solution (as indicated by black triangles) and used this scheme to arrange the samples shown in Figure4 and Table S4. (yielding the three clusters highlighted in green,

yellow and red).

C. Proportions of gender, age, *PRKDC* amplification, and metastasis distribute over the three subgroups.

D. Heatmap depicting the expression patterns of molecular signatures across different subgroups in Kabbarah et al.'s cohort. The heatmap depicts the relative abundance of signature molecules.

E. Heatmap depicting the expression patterns of molecular signatures across different subgroups in TCGA cohort. The heatmap depicts the relative abundance of signature molecules

F. The association of three transcriptomic subtypes with clinical outcomes in 100 primary melanoma patients from the TCGA cohort (p-value based on the log-rank test).

Figure S7. *ROCK2* Amplification Associated with Metastasis in Melanomas, Related to Figure 5.

A. The heatmap showed the protein expression of kinases (ROCK2), and abundance of phosphosites enriched in angiogenesis. The Spearman's correlation coefficients between ROCK2's protein expression and abundance of phosphosites are calculated and display on the right panel, with p values displayed in log10 scale.

B. The heatmap showed the abundance of the phosphosite HMGB1/S100 (TF), and the expression pattern of the target gene (TG) of HMGB1. The Spearman's correlation coefficients between the abundance of phosphosites HMGB1/S100 and expression of TGs are calculated and display on the left panel, with p values displayed in log10 scale.

C. The boxplot indicates the expression level ROCK2 across OE-Control-A375, OE-ROCK2-A375, KD-Control-A375, and ROCK2-KD-A375.

D. The boxplot indicates the phosphorylation of HMGB1 at S100 across OE-Control-A375, OE-ROCK2-A375, KD-Control-A375, and ROCK2-KD-A375.

Figure S8. Immune-Based Subtyping of Melanomas, Related to Figure 6.

A-B. Heatmap of consensus cluster plus, cophenetic correlation coefficient and average silhouette width plots. The input is the xCell score matrix. Based on a visual inspection of the hierarchical clustering and the profiles of the cophenetic correlation coefficient and average silhouette width for solutions with 2 to 5 clusters, we considered K = 3 to be the preferred solution (as indicated by black triangles) and used this scheme to arrange the samples shown in Figure6 and Table S6. (yielding the three clusters highlighted in green, orange and purple).

C. Plot showed the PRKDC expression across the three immune subtypes (n = 75).

D. Spearman-rank correlation of the PRKDC expression and xCell score of CD8+ T-cells and CD274 expression in our discovery cohort.

E. The boxplot showed the macrophage polarization scores in the three immune clusters (n = 75).

F. The boxplot showed the protein expression of CCL2, CCL14, CCL15, and CCL22 in the three immune clusters (n = 75).

G. Immunohistochemistry of CD163 in the three immune clusters, scale bar = 100 μm.

H. Immunohistochemistry of IL17D in the three immune clusters, scale bar = 100 μm.

Figure S9. The Refined Subtype including the Information of both the Immune and Proteomic Subtype and Correlated with OS.

A. Kaplan-Meier curves for overall survival (OS) among five subtypes (p-value based on the log-rank test).

B. Heatmap illustrating frequencies of *PRKDC* amplification, *CDK4* amplification, and *ROCK2* amplification, and xCell immune signatures (n = 75).

C. Plots showed frequencies of *PRKDC* amplification, *CDK4* amplification, and *ROCK2* amplification.

D. Plots showed protein expression of PRKDC, CDK4, and ROCK2 among five subtypes.

E. Heatmap illustrating xCell score of CD8+ T-cells and Tgd cells, and mRNA expression of CD8A, HLA-A, HLA-B, HLA-C, CD274 and IL17D.

F. The table represented Gene Ontology bioprocesses that were significantly altered in HC4 and HC5.

G. The bar plots showed GO terms enriched by phosphoproteins which showed diverse expression patterns in HC4 and HC5.

H. Kaplan-Meier curves for overall survival (OS) based on the kinase activity of AKT3 (p-value based on the log-rank test).

I. Spearman-rank correlation of the AKT3's kinase activity and xCell score of Tgd cells and the ssGSEA score of cell cycle.

J. Heatmap illustrated the protein expression of cell cycle related proteins in HC4 and HC5.

Figure S10. Summary of the findings generated in this study.

A. Summary of the findings generated in this study.

Supplementary Figure 1

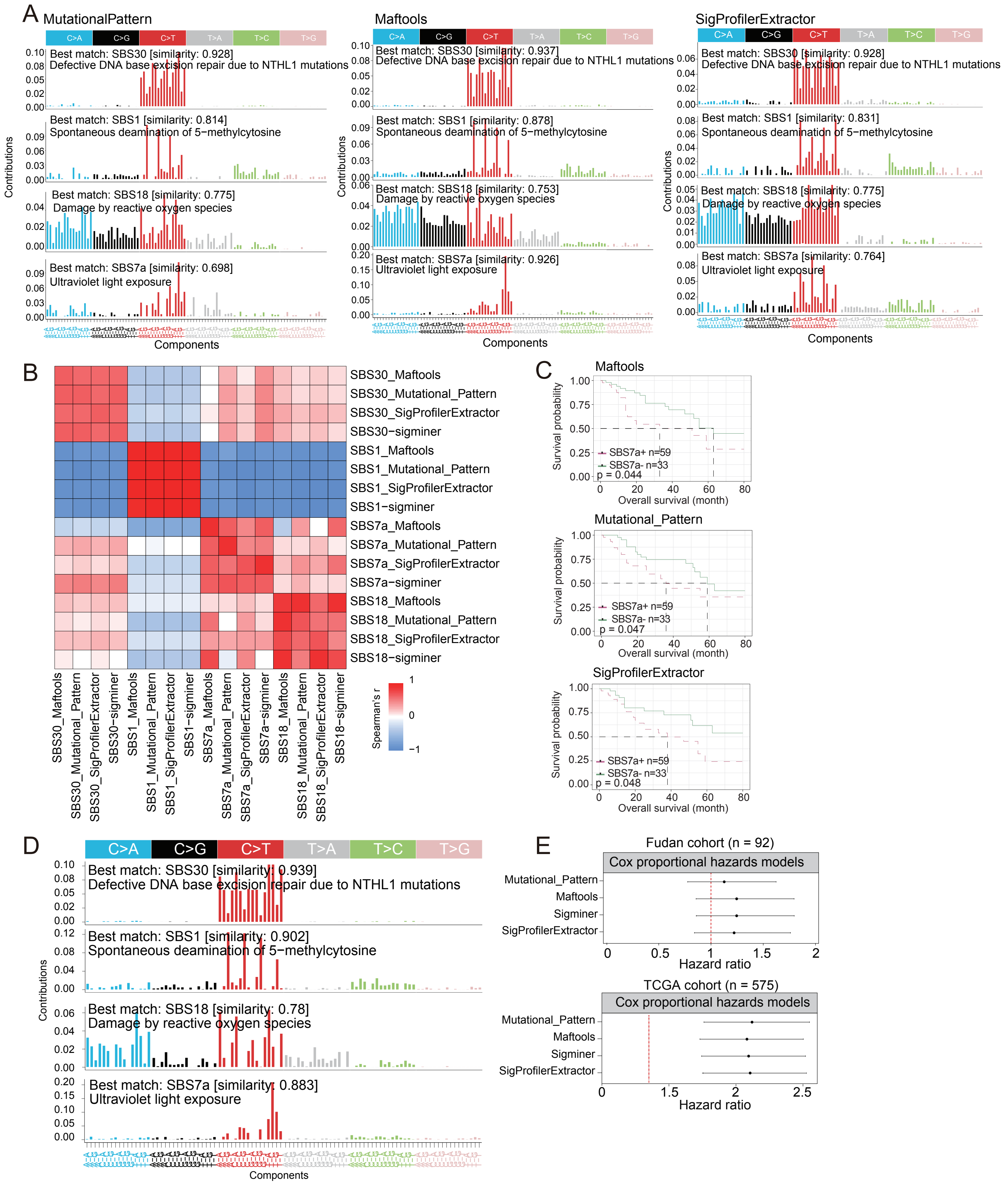

Supplementary Figure 2

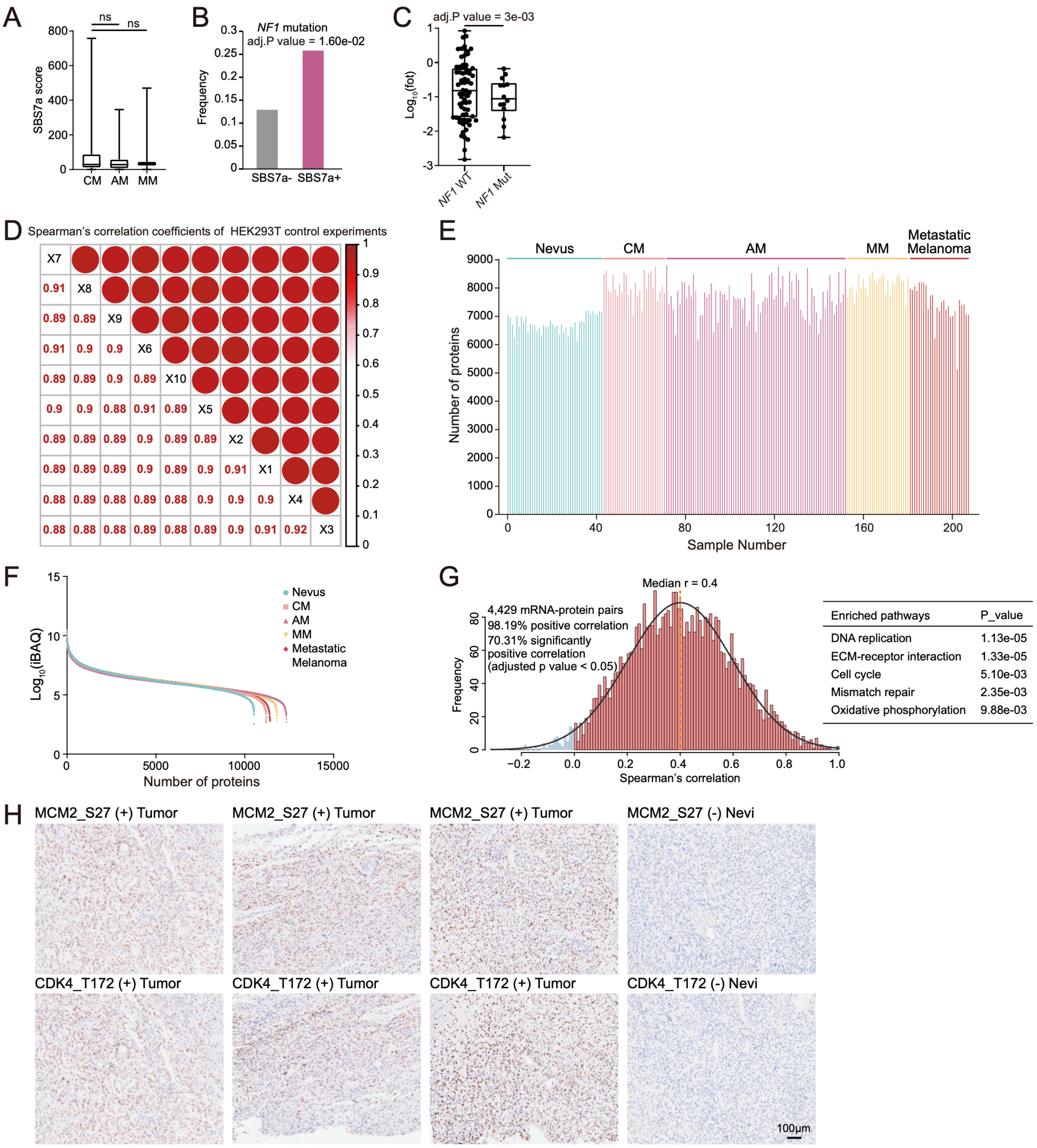

Supplementary Figure 3

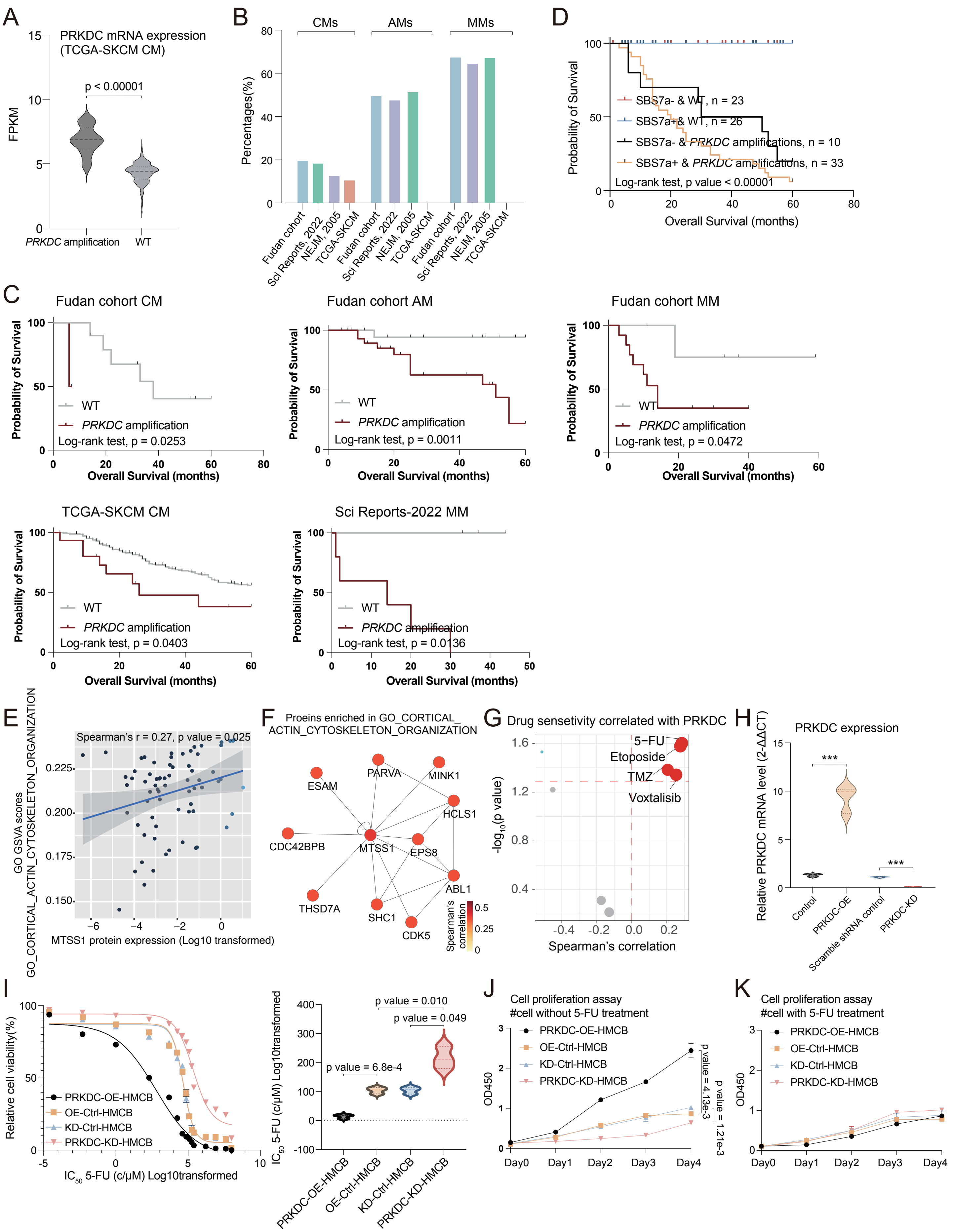

Supplementary Figure 4

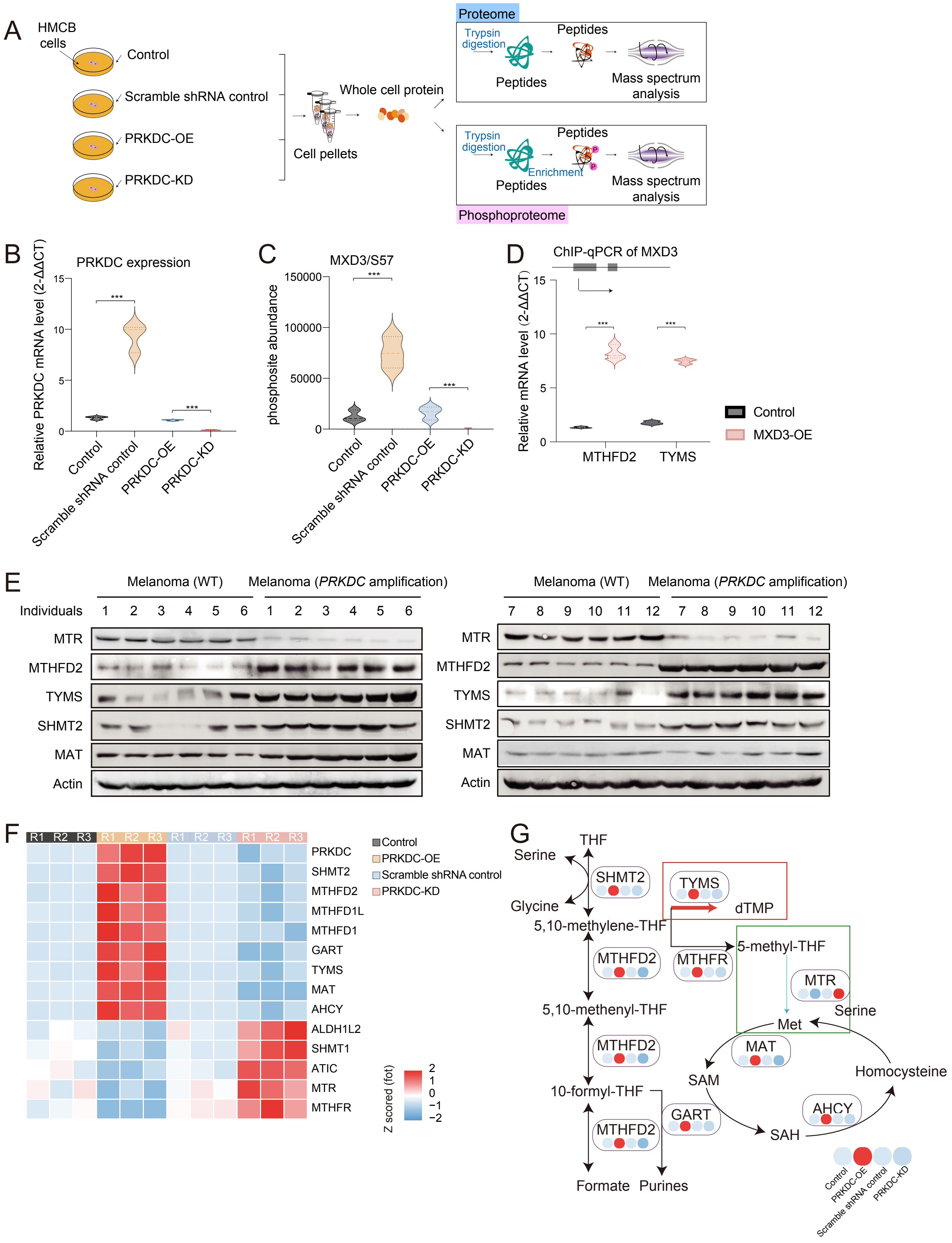

Supplementary Figure 5

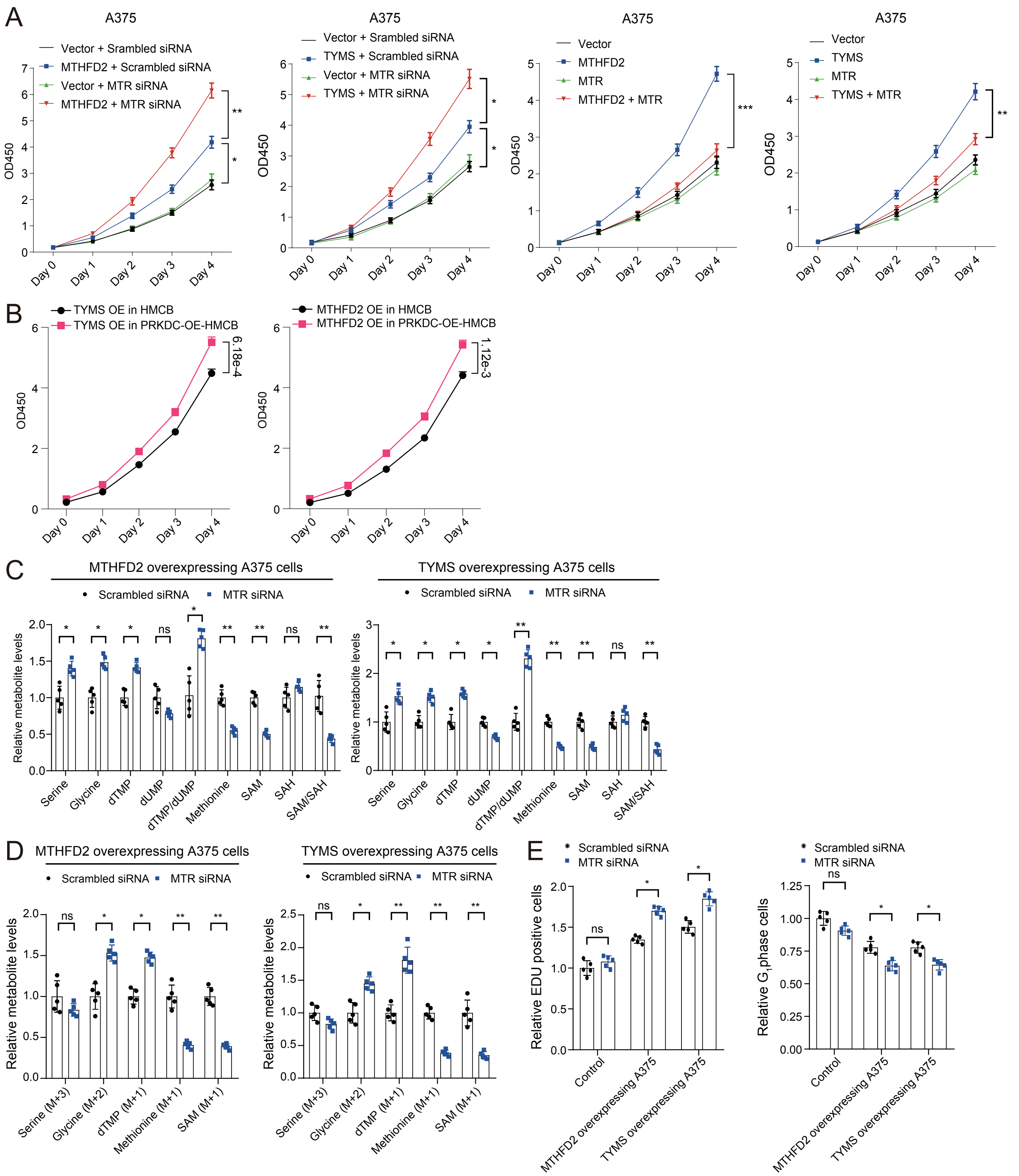

Supplementary Figure 6

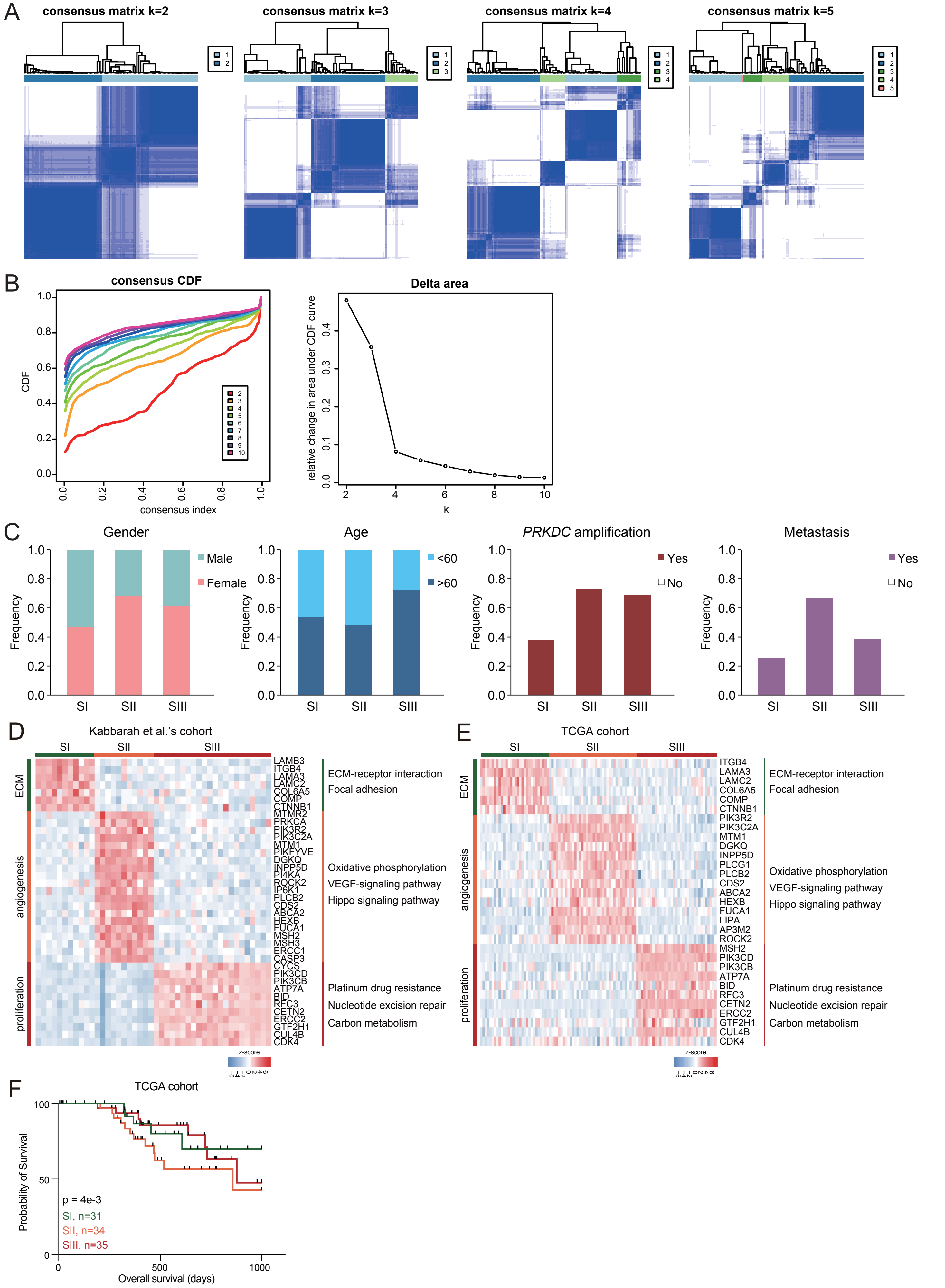

Supplementary Figure 7

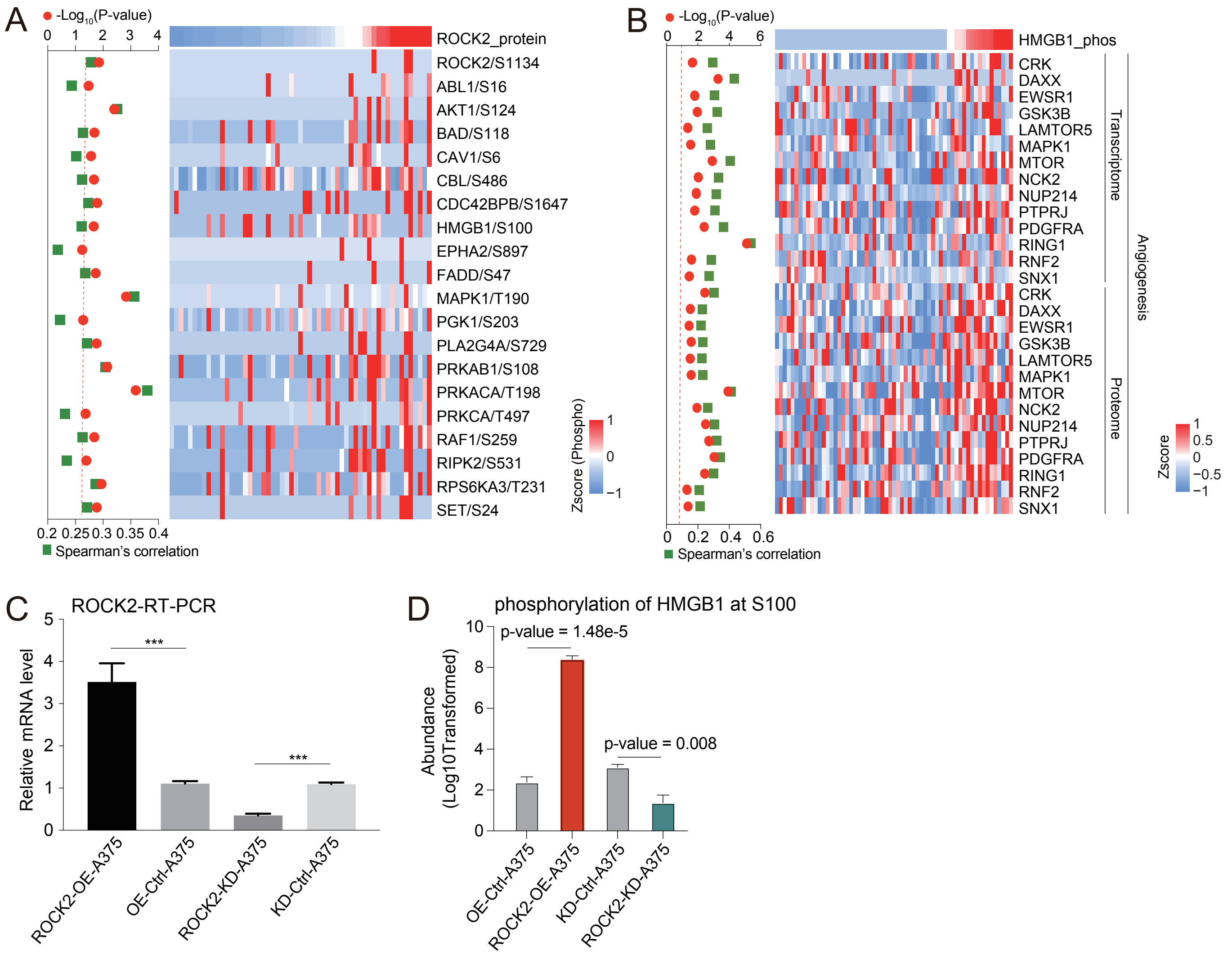

Supplementary Figure 8

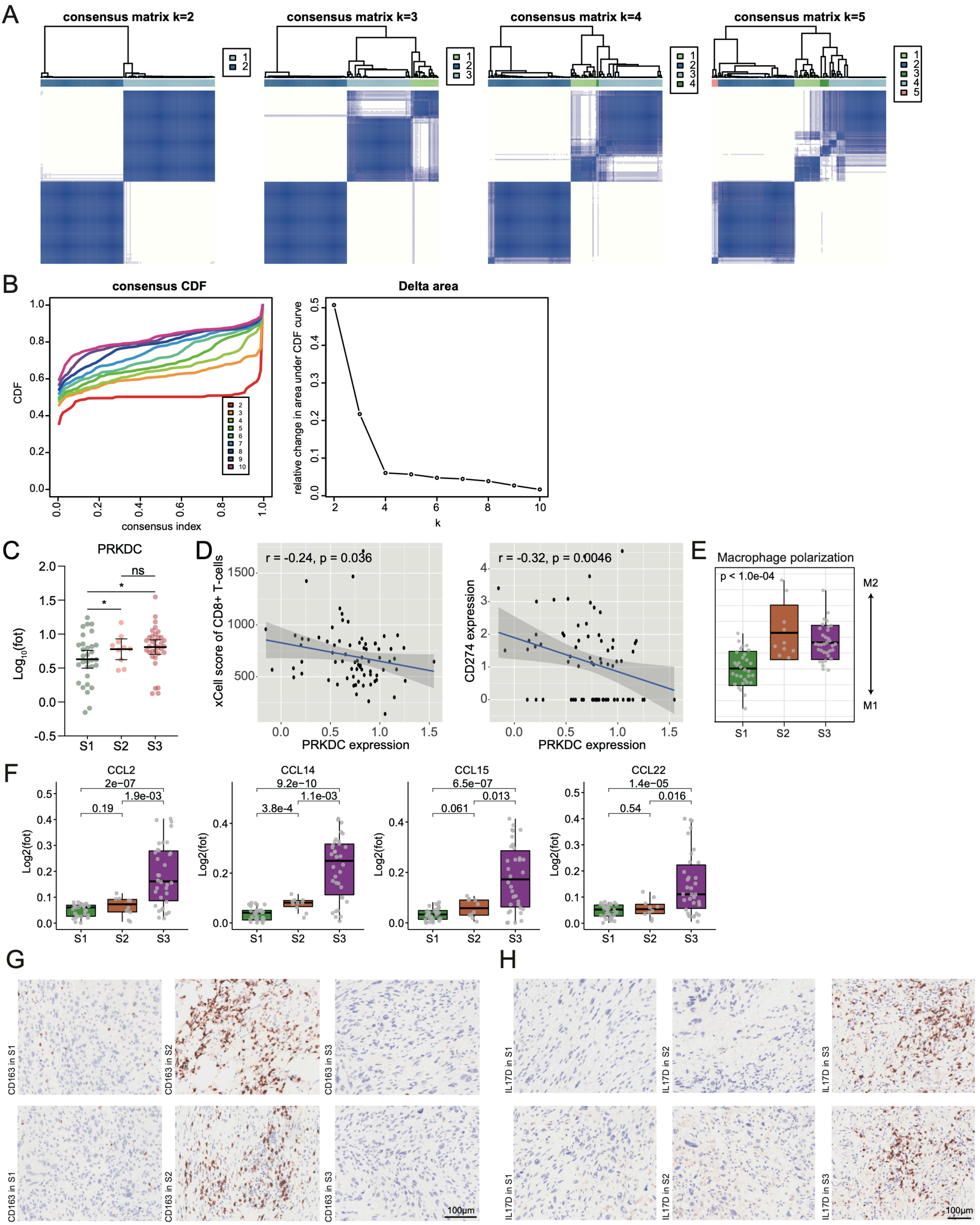

Supplementary Figure 9

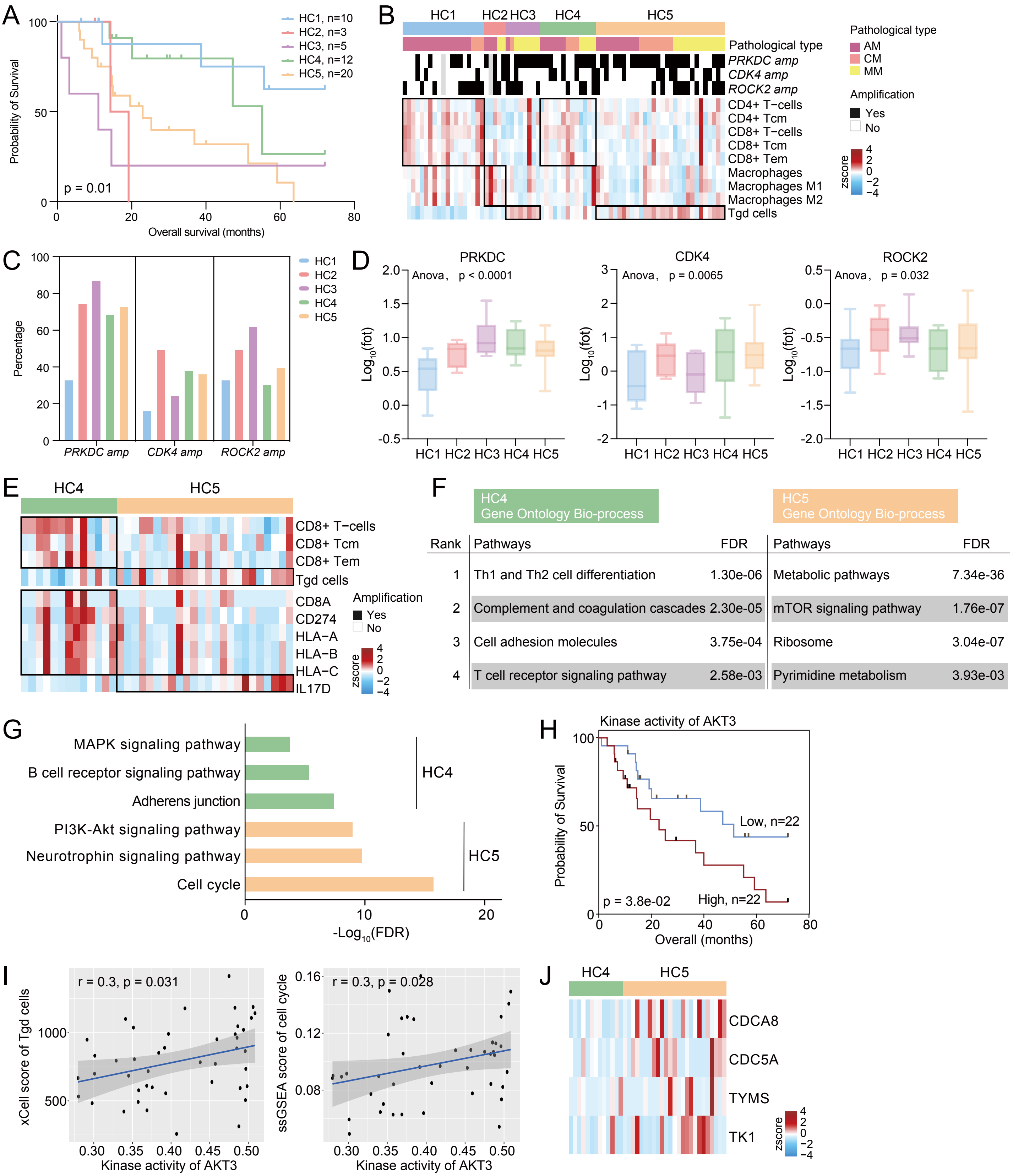

Supplementary Figure 10

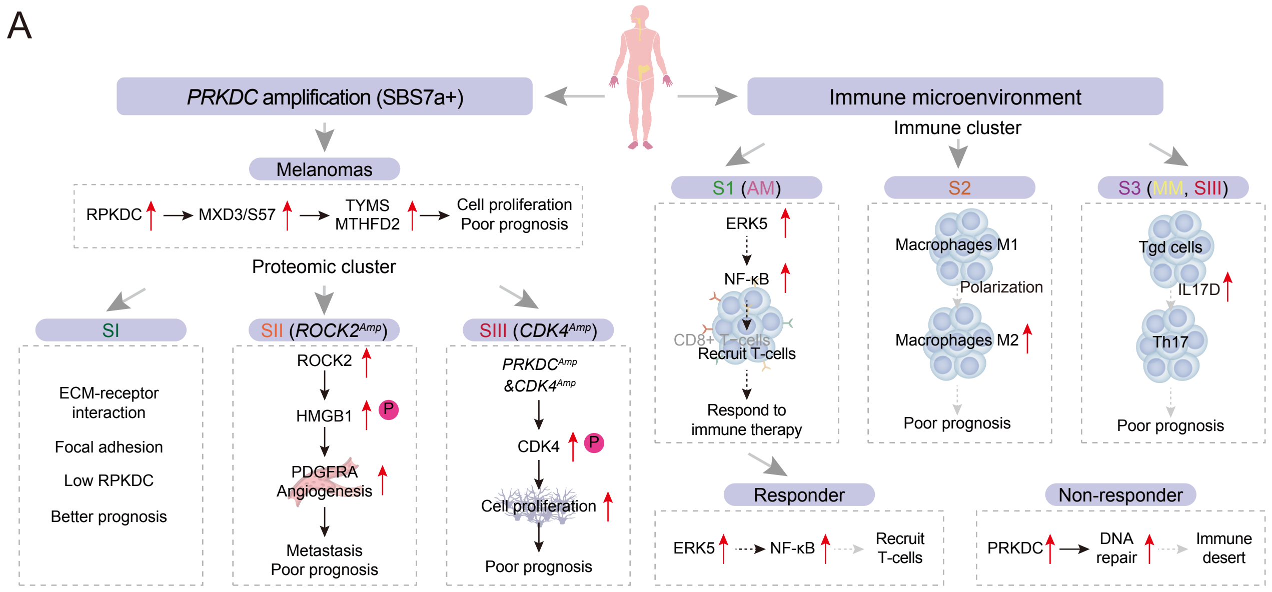

Supplement: Supplementary file 1 — Supplementary Information [file 41421_2024_688_MOESM1_ESM.pdf]
